# Pharmacological Ascorbate Restrains Epithelial–Mesenchymal Transition and Invasion in Glioblastoma Cells via Extracellular H_2_O_2_ Generation

**DOI:** 10.3390/ijms27114964

**Published:** 2026-05-30

**Authors:** Onsurang Wattanathamsan, Naphat Chantaravisoot, Rungnapa Bootsri, Nuttiya Kalpongnukul, Napatsakon Youngsanbhu, Claudia R. Oliva, Corinne E. Griguer, Visarut Buranasudja

**Affiliations:** 1 Department of Pharmacology and Physiology, Faculty of Pharmaceutical Sciences, Chulalongkorn University, Bangkok 10330, Thailand; onsurang.w@chula.ac.th; 2 Department of Biochemistry, Faculty of Medicine, Chulalongkorn University, Bangkok 10330, Thailand; naphat.c@chula.ac.th (N.C.); klairung.napa@gmail.com (R.B.); immnapatsakon000@gmail.com (N.Y.); 3 Center of Excellence in Systems Microbiology, Faculty of Medicine, Chulalongkorn University, Bangkok 10330, Thailand; 4 Center of Excellence in Systems Biology, Faculty of Medicine, Chulalongkorn University, Bangkok 10330, Thailand; nuttiya.nkal@gmail.com; 5 Free Radical and Radiation Biology Program, Department of Radiation Oncology, The University of Iowa, Iowa City, IA 52242, USA; claudia-oliva@uiowa.edu (C.R.O.); corinne-griguer@uiowa.edu (C.E.G.); 6 Center of Excellence in Natural Products for Ageing and Chronic Diseases, Faculty of Pharmaceutical Sciences, Chulalongkorn University, Bangkok 10330, Thailand

**Keywords:** pharmacological ascorbate, vitamin C, glioblastoma, oxidative stress, pro-oxidant, epithelial–mesenchymal transition, migration, invasion, hydrogen peroxide, cancer therapy

## Abstract

Glioblastoma (GBM) is highly invasive, and diffuse tumor cell migration into surrounding brain tissue remains a major obstacle to durable therapeutic control. Pharmacological ascorbate (P-AscH^−^) exhibits anticancer activity through pro-oxidant mechanisms; however, its effects on GBM motility and invasion remain incompletely defined. Transcriptomic analyses revealed a strong association between glioma aggressiveness and gene programs governing migration and invasion. Here, we demonstrate that P-AscH^−^ markedly suppresses migration and invasion of GBM cells. These phenotypic effects are accompanied by coordinated repression of epithelial–mesenchymal transition (EMT) programs, characterized by reduced expression of mesenchymal markers (ZEB1, N-cadherin, Vimentin, Slug, and Twist1) and induction of the epithelial marker Claudin-1 at both transcriptional and protein levels. In parallel, P-AscH^−^ significantly downregulates invasion-associated matrix metalloproteinases MMP2 and MMP9 at the mRNA level. Mechanistically, catalase rescue experiments establish extracellular hydrogen peroxide as an essential mediator of P-AscH^−^-induced inhibition of GBM motility and EMT-associated gene and protein expression. In addition, P-AscH^−^ attenuates mTOR signaling, and combination with a dual mTORC1/2 inhibitor further reinforces suppression of migratory behavior and mesenchymal programs. Importantly, these phenotypic and molecular effects are conserved in a patient-derived glioblastoma model, underscoring translational relevance. Collectively, these findings identify extracellular hydrogen peroxide-driven redox signaling as a key mechanism by which pharmacological ascorbate suppresses EMT and invasive programs in GBM, providing mechanistic support for ongoing clinical evaluation and highlighting its potential utility as an invasion-targeted therapeutic strategy in GBM and other highly plastic malignancies.

## 1. Introduction

Glioblastoma (GBM) is the most malignant and common form of primary brain tumor in adults, accounting for nearly half of all malignant gliomas. Despite aggressive multimodal therapy including maximal surgical resection, radiotherapy, and temozolomide (TMZ) chemotherapy, the prognosis for GBM patients remains dismal, with a median overall survival of only 12–15 months and fewer than 5% of patients surviving beyond five years after diagnosis [1,2,3]. This poor clinical outcome is largely attributed to the tumor’s highly invasive and therapy-resistant nature, which enables rapid recurrence even after extensive treatment [4]. Current therapeutic options primarily delay disease progression rather than providing a durable cure [4]. Hence, the development of novel strategies that can effectively suppress GBM invasiveness and overcome resistance mechanisms is urgently needed to improve patient outcomes and quality of life.

One of the hallmarks of GBM aggressiveness lies in its remarkable cellular plasticity and capacity for invasion [5]. Processes resembling epithelial–mesenchymal transition (EMT), although classically described in epithelial cancers, have been increasingly implicated in glioma biology [6,7]. Through partial EMT-like reprogramming, glioma cells acquire enhanced motility, invasiveness, and resistance to apoptosis, contributing to diffuse tumor infiltration into surrounding brain parenchyma [8,9,10,11]. Key transcription factors such as Snail, Slug, Twist, and ZEB1 together with mesenchymal markers such as N-cadherin and Vimentin drive this mesenchymal phenotype by repressing junctional proteins and promoting cytoskeletal remodeling [12,13]. Importantly, activation of oncogenic signaling cascades such as PI3K/Akt/mTOR further amplifies EMT and invasion programs in GBM, linking intracellular signaling dynamics to extracellular matrix degradation and metastasis-like spread within the brain [14,15,16]. Therefore, targeting EMT and motility-related pathways represents a promising approach to attenuate glioblastoma progression and sensitize tumors to conventional therapies.

Pharmacological ascorbate (P-AscH^−^), defined as millimolar plasma concentrations of ascorbate achievable only through intravenous administration, has re-emerged as a promising redox-based anticancer therapeutic with distinctive mechanistic versatility. At these supraphysiologic levels, ascorbate functions predominantly as a pro-oxidant, driving the extracellular generation of hydrogen peroxide (H_2_O_2_) and imposing oxidative stress on tumor cells [17]. The relative selectivity of P-AscH^−^ toward malignant cells is thought to arise from intrinsic redox vulnerabilities of tumors, including diminished antioxidant defenses, most notably reduced catalase activity [18], and elevated labile iron pools [19], compared with normal tissues. Consistent antitumor activity has been reported across multiple malignancies, including pancreatic cancer [20,21], non-small cell lung cancer [19,22], endometrial cancer [23], and glioblastoma [19,24], where pharmacological ascorbate suppresses tumor growth primarily through its pro-oxidant mechanisms. Beyond direct oxidative cytotoxicity, accumulating evidence indicates that P-AscH^−^ also functions as a redox modulator capable of reprogramming key survival and stress-response pathways, including PI3K/Akt/mTOR signaling [23,24,25]. Collectively, the convergence of direct oxidative injury with secondary signaling modulation underscores the growing interest in P-AscH^−^ as a mechanistically multifaceted and clinically adaptable anticancer strategy.

In the context of GBM, preclinical studies have demonstrated that P-AscH^−^ effectively suppresses tumor growth in vitro and in vivo [19,24] and early clinical evidence supports its potential utility as an adjuvant therapeutic strategy [26]. However, despite these advances, the role of P-AscH^−^ in regulating GBM invasion and epithelial–mesenchymal transition (EMT)-critical drivers of tumor aggressiveness, infiltration, and therapeutic resistance-remains poorly defined. In this present study, we investigated the effects of P-AscH^−^ on EMT, migration, and invasion using both established and patient-derived GBM models. We demonstrate that P-AscH^−^ markedly impairs GBM cell motility through extracellular H_2_O_2_ generation, leading to suppression of Akt/mTOR signaling and coordinated downregulation of EMT-associated markers at both transcriptional and protein levels. Moreover, co-treatment with the dual mTORC1/2 inhibitor AZD8055 produced an enhancement in inhibition of mesenchymal features, as well as migratory and invasive capacities, suggesting that P-AscH^−^ can potentiate the antitumor efficacy of mTOR-targeted therapy. Collectively, our findings identify redox-driven modulation of EMT and invasive behavior as a previously underappreciated mechanism of P-AscH^−^ action in GBM and provide a mechanistic rationale for integrating P-AscH^−^ with pathway-directed therapies to counteract GBM progression.

## 2. Results

### 2.1. Comparative Transcriptomic Profiling Identifies EMT Enrichment in Highly Invasive GBM Models

**Figure 1:**
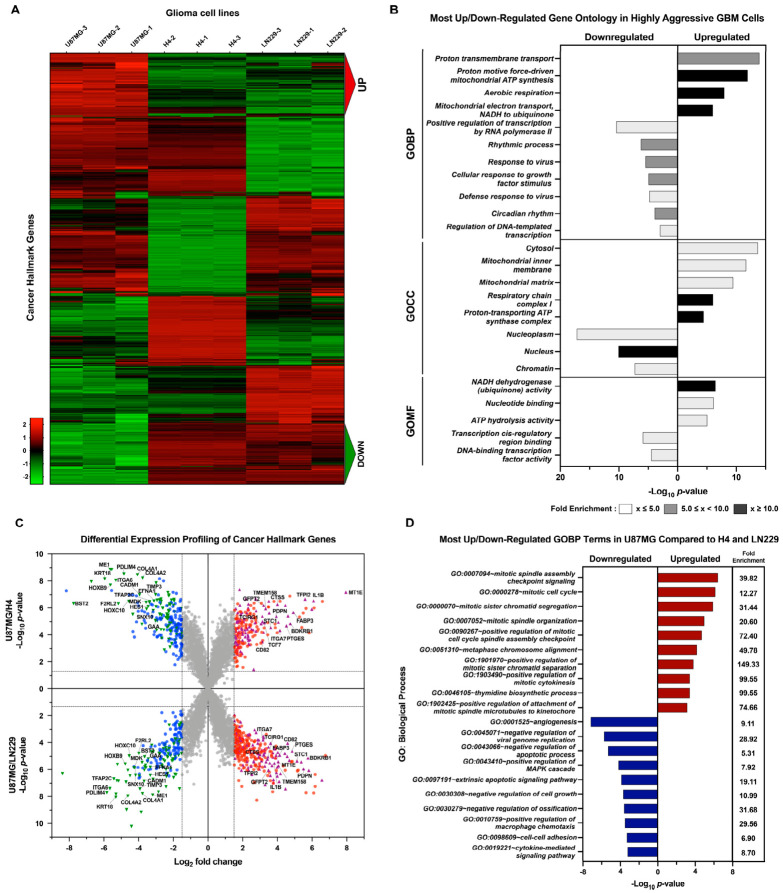
Transcriptomic profiling of cancer hallmark gene expression in glioma cell lines. (**A**) Hierarchically clustered heatmap showing the expression of cancer hallmark genes across three glioma cell lines (H4, LN229, and U87MG). RNA-seq data from three biological replicates per cell line were subjected to hierarchical clustering using Clustergrammer, and heatmaps were visualized in GraphPad Prism. (**B**) Gene Ontology (GO) enrichment analysis of genes differentially expressed across the three cell lines. Enriched terms are grouped by biological process (GOBP), cellular component (GOCC), and molecular function (GOMF). Genes most strongly upregulated or downregulated in U87MG relative to H4 and LN229 are indicated in Figure 1A (UP, red; DOWN, green). Bars represent –log_10_(*p*-value), and colors indicate fold enrichment. (**C**) Double volcano plots showing differential expression of cancer hallmark genes comparing U87MG vs. H4 (top) and U87MG vs. LN229 (bottom). Significantly upregulated genes (log_2_FC > 1.5, *p* < 0.05) are shown in red, while significantly downregulated genes (log_2_FC < –1.5, *p* < 0.05) are shown in blue. Genes commonly upregulated or downregulated in both comparisons are indicated by purple upward triangles and green downward triangles, respectively. (**D**) GOBP enrichment analysis of genes commonly up- or downregulated in U87MG relative to both H4 and LN229 (|log_2_FC| > 1.5, *p* < 0.05). Top enriched terms passing false discovery rate correction (FDR < 0.05) are shown. Bar length represents –log_10_(*p*-value), and color intensity indicates fold enrichment.

Glioblastoma (GBM) is among the most aggressive and highly invasive primary brain tumors, characterized by diffuse infiltration into surrounding brain parenchyma [4]. To delineate the transcriptional landscape associated with glioblastoma aggressiveness and invasion, we performed unbiased bulk RNA sequencing across brain tumor cell lines representing distinct malignant phenotypes: H4 (low-grade glioma model), and LN229 and U87MG (high-grade glioblastoma models). Unsupervised hierarchical clustering and principal component analysis of normalized RNA-seq data (log_2_[FPKM] + 1) revealed clear separation among the three cell lines, with increased transcriptional heterogeneity observed in the more invasive models (Figure 1A and Figure S8A). These global patterns suggested that U87MG harbors molecular features most strongly associated with aggressive behavior, leading us to perform subsequent comparative analyses involving this cell line.

Differential expression and Gene Ontology (GO) analyses revealed that genes upregulated in U87MG were predominantly associated with mitochondrial function and energy metabolism, whereas downregulated processes were enriched for transcriptional regulation, growth factor responsiveness, and antiviral signaling (Figure 1B). Consistent with these findings, mitochondrial compartments and enzymatic activities were enriched among upregulated genes, while nuclear and chromatin-associated components and transcription factor activity were suppressed. KEGG pathway analysis further identified oxidative phosphorylation and reactive oxygen species-related pathways as prominently enriched in U87MG (Figure S8D).

Pairwise comparisons of U87MG versus H4 and LN229 identified a shared set of significantly altered genes uniquely associated with the most aggressive phenotype. U87MG exhibited strong upregulation of genes linked to invasion and malignancy, including *MT1E*, *PDPN*, *BDKRB1*, *ITGA7*, *PTGES*, *TMEM158*, and *TFPI2*, alongside downregulation of structural and extracellular matrix-associated genes such as *KRT18*, *COL4A1*, *COL4A2*, *TIMP3*, and *PDLIM4* (Figure 1C). Functional enrichment of these differentially expressed genes revealed activation of microtubule-based processes related to cell division and motility, accompanied by suppression of angiogenesis regulation, apoptotic control, and cell–cell adhesion (Figure 1D and Figure S8E), consistent with a highly migratory and invasive phenotype.

Direct comparison between the two GBM models, U87MG and LN229, further demonstrated that U87MG uniquely upregulated biological processes associated with mitotic spindle dynamics, DNA replication and repair, cytokinesis, angiogenesis, and epithelial-to-mesenchymal transition (EMT). In contrast, U87MG showed marked downregulation of pathways involved in immune responsiveness, growth suppression, cell adhesion, canonical Wnt signaling, and MAPK and NF-κB signaling, indicating broader acquisition of cancer hallmark capabilities relative to LN229 (Figure S9A,B).

**Figure 2:**
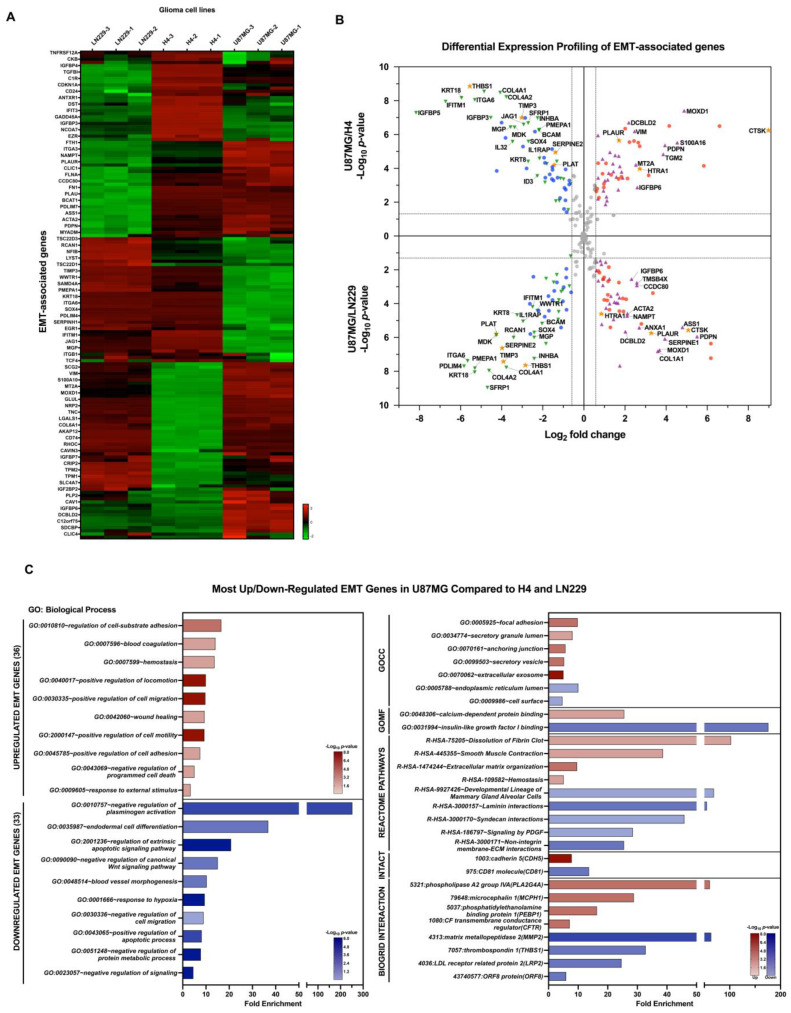
Transcriptomic analysis of epithelial–mesenchymal transition (EMT) gene signatures in glioma cell lines. (**A**) Hierarchically clustered heatmap showing expression of 152 EMT-associated genes across H4, LN229, and U87MG cell lines. RNA-seq data from three biological replicates per cell line were clustered using Clustergrammer and visualized in GraphPad Prism. (**B**) Double volcano plots comparing EMT gene expression between U87MG and H4 (top) and between U87MG and LN229 (bottom). Significantly upregulated genes (log_2_FC > 1.5, *p* < 0.05) are shown in red and downregulated genes (log_2_FC < –1.5, *p* < 0.05) in blue. Genes consistently altered in both comparisons are indicated by purple upward triangles (upregulated) and green downward triangles (downregulated). Orange stars highlight key EMT-associated biomarkers linked to invasion and metastatic capabilities. (**C**) Functional enrichment analysis of commonly altered EMT-associated genes (|log_2_FC| > 1.5, *p* < 0.05). Enriched terms from GO (GOBP, GOCC, GOMF), Reactome, IntAct, and BioGRID databases passing FDR correction (FDR < 0.05) are shown. Bar length represents fold enrichment, and color intensity corresponds to –log_10_(*p*-value).

Given the prominent enrichment of EMT-related pathways, we performed targeted analysis of a curated panel of 152 EMT-associated genes. Hierarchical clustering revealed a distinct EMT transcriptional signature in U87MG compared with H4 and LN229 (Figure 2A). Differential expression analysis identified consistent EMT-related gene changes uniquely associated with U87MG (Figure 2B). Functional enrichment demonstrated activation of processes promoting cell–substrate adhesion, locomotion, wound healing, and motility, alongside strong suppression of negative regulators of plasminogen activation, hypoxic response, apoptotic signaling, and differentiation pathways. Collectively, these findings indicate that U87MG cells exhibit a pronounced EMT-driven transcriptional program consistent with enhanced invasive potential.

Based on this comprehensive transcriptomic characterization, U87MG was selected as an experimental model for subsequent mechanistic studies examining the effects of pharmacological ascorbate (P-AscH^−^) on EMT and invasive behavior in GBM. To ensure that observed effects were not restricted to a single genetic background, key experiments were replicated in U251 cells, a genetically and transcriptionally distinct GBM model widely used in studies of EMT, migration, and invasion [27,28,29,30,31,32,33,34]. Inclusion of this additional model strengthens the robustness of our findings and enhances translational relevance by demonstrating that the mechanistic effects of P-AscH^−^ extend across multiple GBM systems rather than being confined to a single aggressive subtype.

### 2.2. Identification of Sub-Cytotoxic Pharmacological Ascorbate Conditions for Migration and Invasion Studies

**Figure 3:**
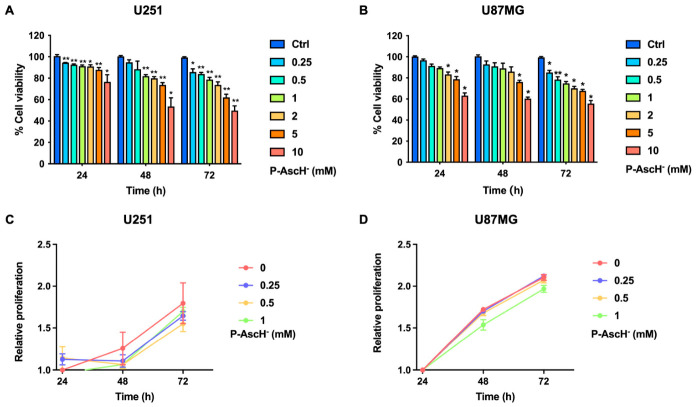
Identification of sub-cytotoxic pharmacological ascorbate conditions for migration and invasion studies. (**A**,**B**) Pharmacological ascorbate (P-AscH^−^) decreases the viability of U251 and U87MG glioblastoma cells in a dose- and time-dependent manner. Cells were exposed to P-AscH^−^ (0.25–10 mM) for 1 h, and cell viability was assessed at the indicated time points using the MTT assay. Concentrations below 2 mM were identified as sub-cytotoxic under these experimental conditions. (**C**,**D**) Sub-cytotoxic concentrations of P-AscH^−^ do not significantly affect short-term proliferative capacity in U251 and U87MG cells. Cells were treated with P-AscH^−^ (0.25–1 mM) for 1 h, and proliferation was monitored over time by MTT assay. These sub-cytotoxic concentrations were therefore selected for subsequent migration and invasion assays to minimize confounding effects from cytotoxicity or growth inhibition. Data are presented as mean ± SEM from three independent biological experiments, each performed with four technical replicates. Ctrl, untreated control. *, *p* < 0.05; **, *p* < 0.01 vs. Ctrl.

To establish sub-cytotoxic exposure conditions suitable for subsequent migration and invasion analyses, we first examined the effects of pharmacological ascorbate (P-AscH^−^) on glioblastoma (GBM) cell viability and proliferation. U251 and U87MG cells were exposed to increasing concentrations of P-AscH^−^ (0.25–10 mM) for 1 h, followed by assessment of cell viability using the MTT assay at multiple time points after treatment (24–72 h). P-AscH^−^ treatment resulted in a significant, dose- and time-dependent reduction in cell viability in both GBM cell lines compared with untreated controls (Figure 3A,B). Concentrations below 2 mM were classified as sub-cytotoxic, as cell viability remained above 70% across all assessed time points [35,36,37,38,39]. To determine whether these sub-cytotoxic concentrations influenced proliferative capacity, proliferation assays were performed, revealing that P-AscH^−^ at concentrations below 2 mM did not significantly affect GBM cell proliferation (Figure 3C,D). Based on these findings, subsequent migration and invasion experiments were conducted using P-AscH^−^ concentrations below 2 mM to minimize confounding effects from cytotoxicity or growth inhibition.

### 2.3. Pharmacological Ascorbate Attenuates Migration and Invasion of Glioblastoma Cells

**Figure 4:**
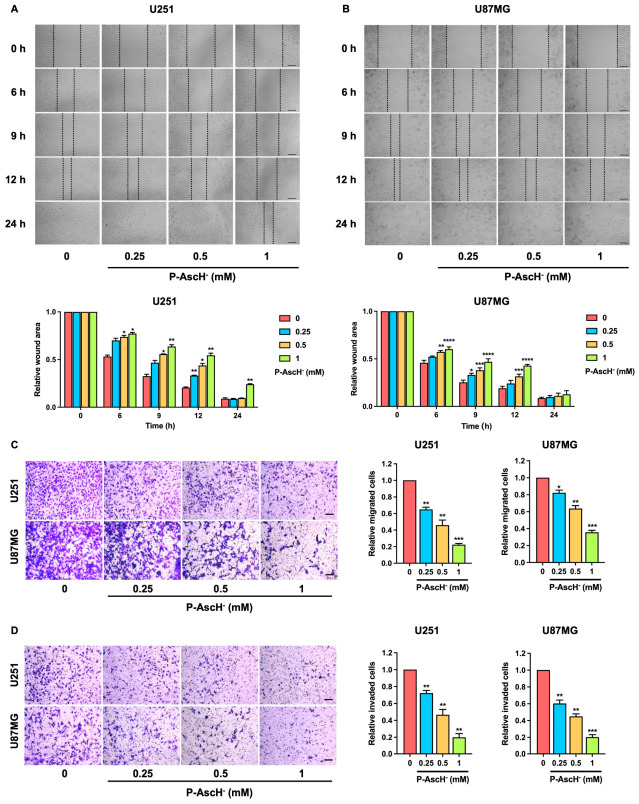
Pharmacological ascorbate suppresses migration and invasion of glioblastoma cells. (**A**,**B**) P-AscH^−^ inhibits collective migration of glioblastoma cells. U251 and U87MG cells were subjected to scratch wounding and treated with sub-cytotoxic concentrations of P-AscH^−^ (0.25–1 mM, 1 h). Wound images were acquired at the indicated time points, and wound closure was quantified as the remaining wound area normalized to the initial wound area at 0 h. Microscopic images represent three independent wound-healing experiments. (**C**,**D**) P-AscH^−^ suppresses both migratory and invasive capacities of glioblastoma cells. U251 and U87MG cells were treated with P-AscH^−^ (0.25–1 mM, 1 h), followed by assessment of migration and invasion using Transwell-based assays. Migration was evaluated using uncoated inserts (**C**), whereas invasion was assessed using Matrigel^®^-coated inserts (**D**). Representative microscopic images are shown from three independent experiments. Migrated and invaded cells were quantified and expressed relative to untreated controls. Data are presented as mean ± SEM. Ctrl, untreated control. Scale bar = 50 μm. *, *p* < 0.05; **, *p* < 0.01; ***, *p* < 0.001; ****, *p* < 0.0001 vs. Ctrl.

Given that cell motility and invasiveness are central determinants of GBM aggressiveness [4], we next examined whether P-AscH^−^ modulates migratory and invasive behavior in GBM cells. Using wound-healing assays, P-AscH^−^ treatment significantly impaired collective cell migration in both U251 and U87MG cells in a concentration-dependent manner (Figure 4A,B). These anti-migratory effects were independently validated using transwell migration assays, which yielded results consistent with the wound-healing analysis. P-AscH^−^ markedly suppressed migratory activity in both GBM cell lines in a dose-dependent fashion (Figure 4C). At 1 mM, P-AscH^−^ reduced migration by approximately 80% in U251 cells and 65% in U87MG cells relative to untreated controls.

We next assessed whether the inhibitory effects of P-AscH^−^ extended to invasive behavior using Matrigel^®^-coated transwell assays (Corning, NY, USA). In line with its anti-migratory activity, P-AscH^−^ markedly reduced the number of invading cells in both U251 and U87MG lines (Figure 4D). At 1 mM, invasive capacity was decreased by approximately 80% in both U251 and U87MG cells relative to untreated controls. Collectively, these findings demonstrate that pharmacological ascorbate robustly suppresses both migration and invasion in GBM cells, supporting its potential role in limiting aggressive and invasive tumor phenotypes.

### 2.4. Pharmacological Ascorbate Impairs EMT-Associated Matrix Remodeling in Glioblastoma

**Figure 5:**
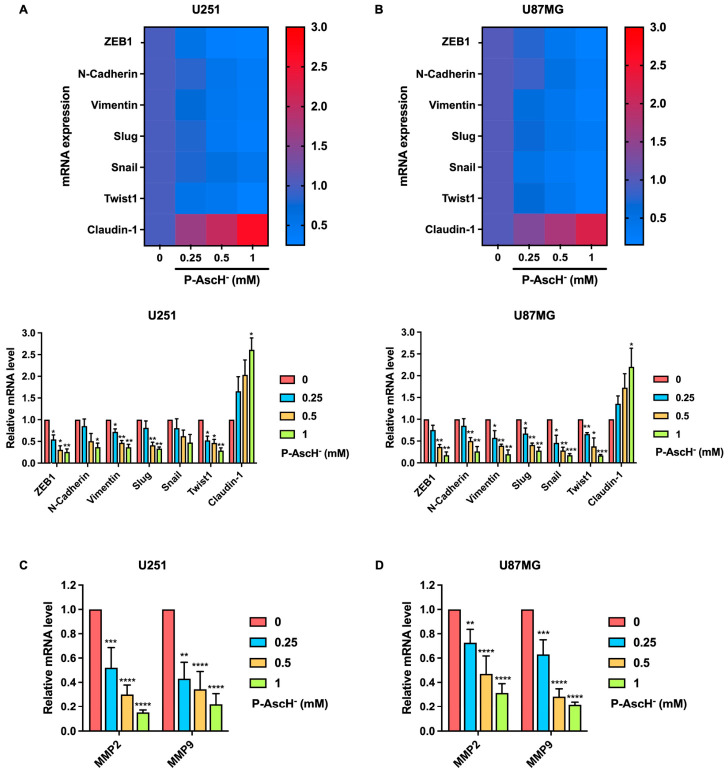
Pharmacological ascorbate suppresses mesenchymal gene expression and invasion-associated MMP transcripts in glioblastoma cells. (**A**,**B**) P-AscH^−^ represses mesenchymal marker expression (*N-cadherin*, *Vimentin*, *Slug*, *Snail*, *Twist*, *and ZEB1*) while inducing epithelial marker *Claudin-1* expression in glioblastoma cells. U251 and U87MG cells were treated with P-AscH^−^ (0.25–1 mM) for 1 h, followed by RNA extraction 6 h after treatment. Transcript levels of epithelial–mesenchymal transition (EMT)-associated genes were quantified by qRT-PCR. (**C**,**D**) P-AscH^−^ reduces expression of invasion-associated matrix metalloproteinase transcripts, *MMP2* and *MMP9*, under the same experimental protocols. Data are presented as mean ± SEM (*n* = 3 independent biological experiments, each with three technical replicates). Ctrl, untreated control. *, *p* < 0.05; **, *p* < 0.01; ***, *p* < 0.001; ****, *p* < 0.0001 vs. Ctrl.

Because GBM cell migration and invasion are closely regulated by epithelial–mesenchymal transition (EMT) [13], we next examined whether P-AscH^−^ alters EMT-associated gene expression. EMT is a central transcriptional program that promotes mesenchymal transformation, enhances cellular motility, and facilitates glioblastoma invasiveness [6]. Accordingly, we assessed the expression of canonical EMT-related genes following P-AscH^−^ exposure. As shown in Figure 5A, treatment of U251 cells with P-AscH^−^ resulted in a marked downregulation of multiple mesenchymal markers, including *ZEB1*, *N-cadherin*, *Vimentin*, *Slug*, and *Twist1*, with the most pronounced suppression observed at 1 mM. In parallel, expression of the epithelial marker *Claudin-1* was significantly increased, indicating a partial shift toward an epithelial-like transcriptional state. A similar pattern was observed in U87MG cells (Figure 5B), where P-AscH^−^ suppressed mesenchymal gene expression in a dose-dependent manner. Notably, *Claudin-1* induction reached statistical significance at 1 mM P-AscH^−^, while lower concentrations (0.25 and 0.5 mM) did not achieve statistical significance but exhibited consistent upward trends. Collectively, these data indicate that pharmacological ascorbate preferentially represses mesenchymal drivers while promoting epithelial marker expression, consistent with its inhibitory effects on glioblastoma cell migration and invasion (Figure 4).

Extracellular matrix remodeling is a critical downstream component of EMT and a defining feature of glioblastoma invasion [40]. We therefore examined whether P-AscH^−^ modulates the expression of matrix metalloproteinases (MMPs), which mediate ECM degradation during tumor dissemination. Among these, MMP2 and MMP9 are well-established mediators of basement membrane degradation and ECM turnover that facilitate invasion of glioma cells [41,42]. Consistent with the observed suppression of EMT-associated gene expression, P-AscH^−^ treatment significantly reduced *MMP2* and *MMP9* expression in both U251 and U87MG cells in a dose-dependent manner (Figure 5C,D). These findings further support a role for pharmacological ascorbate in limiting glioblastoma invasiveness by attenuating ECM-degrading capacity. Collectively, repression of both EMT-related transcriptional programs and proteolytic enzymes essential for matrix remodeling provides a coherent molecular basis for the inhibitory effects of P-AscH^−^ on glioblastoma cell motility and invasion.

### 2.5. Extracellular H_2_O_2_ Mediates the Anti-Migratory and Anti-Invasive Effects of Pharmacological Ascorbate in Glioblastoma

**Figure 6:**
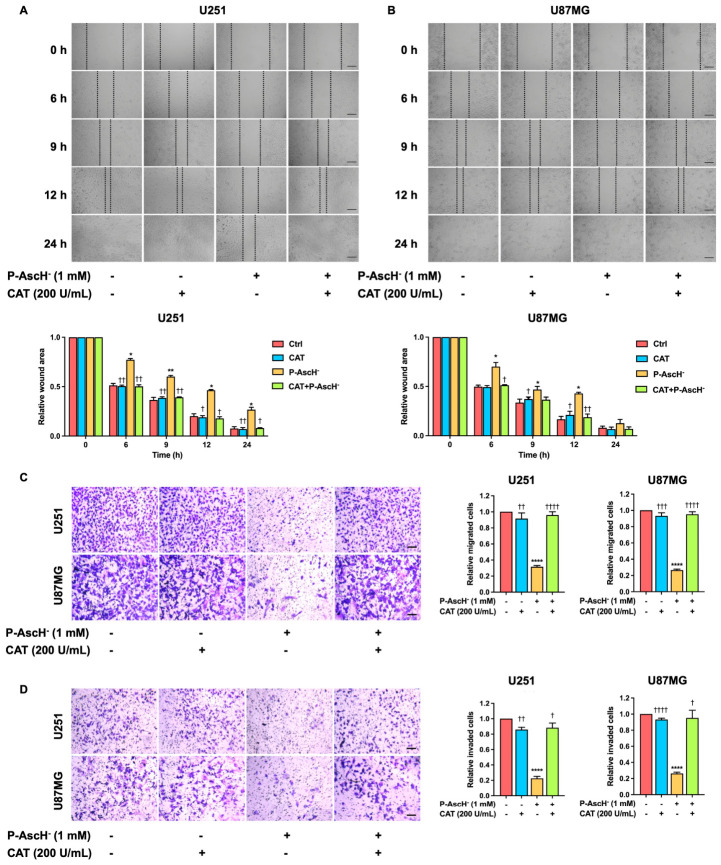
Extracellular hydrogen peroxide mediates the anti-migratory and anti-invasive effects of pharmacological ascorbate. (**A**,**B**) Extracellular hydrogen peroxide is required for the anti-migratory activity of P-AscH^−^ against glioblastoma cells. Following scratch wound generation, cells were treated with P-AscH^−^ (1 mM) in the presence or absence of catalase (CAT; 200 U/mL) for 1 h. Wound closure was monitored over 24 h, and wound area was quantified and expressed relative to the initial wound area at 0 h for each group. Representative phase-contrast images are shown from three independent wound-healing experiments. (**C**,**D**) Catalase abrogates the anti-migratory and anti-invasive effects of P-AscH^−^. Cells were treated with P-AscH^−^ (1 mM) with or without CAT (200 U/mL) for 1 h, followed by assessment of migration (**C**) and invasion (**D**) using Transwell-based assays. Migrated and invaded cells on the lower surface of the membrane were quantified and expressed relative to untreated controls. Representative images are shown from three independent experiments. Data are presented as mean ± SEM. Ctrl, untreated control; CAT, catalase. Scale bar = 50 μm. *, *p* < 0.05; **, *p* < 0.01; ****, *p* < 0.0001 vs. Ctrl; †, *p* < 0.05; ††, *p* < 0.01; †††, *p* < 0.001; ††††, *p* < 0.0001 vs. P-AscH^−^ alone.

Pharmacological ascorbate is known to induce oxidative stress in glioma cells predominantly through the extracellular generation of H_2_O_2_ [24]. To determine whether extracellular H_2_O_2_ mediates the inhibitory effects of P-AscH^−^ on GBM cell motility, glioblastoma cells were co-treated with catalase, an antioxidant enzyme that selectively decomposes high fluxes of H_2_O_2_ [18]. In wound-healing assays, P-AscH^−^ treatment significantly impaired migratory capacity in both U251 and U87MG cells, where co-administration of catalase effectively rescued migratory capacity, restoring wound closure to levels comparable to untreated controls (Figure 6A,B). These results suggest that extracellular H_2_O_2_ is a necessary mediator of the anti-migratory effect of P-AscH^−^.

We next extended this mechanistic assessment to invasive behavior using transwell migration and Matrigel invasion assays. Consistent with the wound-healing results, P-AscH^−^ substantially reduced both migration and invasion in GBM cells. In the presence of catalase, both migratory and invasive activities were restored to near-baseline levels (Figure 6C,D), indicating that extracellular H_2_O_2_ is required for P-AscH^−^-induced suppression of cell motility across multiple assay platforms.

Collectively, these results demonstrate that extracellular H_2_O_2_ generation is a critical upstream determinant of the anti-migratory and anti-invasive actions of pharmacological ascorbate in glioblastoma cells, providing a mechanistic link between redox stress and suppression of GBM motility programs.

### 2.6. Pharmacological Ascorbate Regulates EMT-Associated and Invasion-Related Gene Expression via Extracellular H_2_O_2_

EMT is a central driver of GBM cell migration and invasion [5,6], and our preceding results demonstrated that P-AscH^−^ suppresses these invasive phenotypes in an extracellular H_2_O_2_-dependent manner (Figure 6). We therefore next examined whether extracellular H_2_O_2_ also mediates the transcriptional regulation of EMT-associated genes in response to P-AscH^−^ treatment. To address this question, the expression of canonical EMT markers was assessed following P-AscH^−^ exposure in the presence or absence of extracellular catalase.

**Figure 7:**
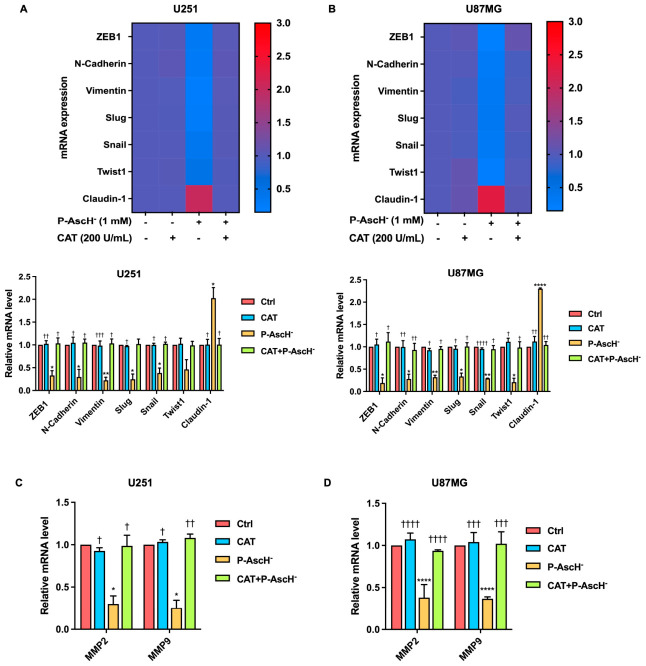
Extracellular H_2_O_2_ regulates EMT- and invasion-associated gene expression following pharmacological ascorbate treatment. (**A**,**B**) Extracellular hydrogen peroxide is required for P-AscH^−^-mediated regulation of epithelial–mesenchymal transition (EMT)-associated gene expression in glioblastoma cells. Cells were treated with P-AscH^−^ (1 mM) in the presence or absence of catalase (CAT; 200 U/mL) for 1 h, and mRNA levels of EMT markers were quantified by qRT-PCR 6 h after treatment. (**C**,**D**) Catalase prevents the P-AscH^−^-induced downregulation of invasion-associated matrix metalloproteinase transcripts, *MMP2* and *MMP9*. Transcript levels were quantified by qRT-PCR under the same treatment conditions as in panels (**A**,**B**). Data are presented as mean ± SEM (*n* = 3 independent biological experiments, each with three technical replicates). Expression levels are shown relative to untreated control. Ctrl, untreated control; CAT, catalase. *, *p* < 0.05; **, *p* < 0.01; ****, *p* < 0.0001 vs. Ctrl; †, *p* < 0.05; ††, *p* < 0.01; †††, *p* < 0.001; ††††, *p* < 0.0001 vs. P-AscH^−^ alone.

As shown in Figure 5A,B, treatment with P-AscH^−^ alone resulted in a pronounced reduction in the mRNA expression of key mesenchymal regulators, including *ZEB1*, *N-cadherin*, *Vimentin*, *Slug*, and *Twist1*, accompanied by a significant increase in the epithelial marker *Claudin-1*, in both U251 (Figure 7A) and U87MG cells (Figure 7B). In contrast, co-treatment with catalase effectively abolished these transcriptional changes, restoring mesenchymal gene expression to levels comparable to untreated controls. Additionally, catalase co-administration also attenuated the P-AscH^−^-induced upregulation of *Claudin-1*. This reversal was consistently observed across both GBM cell lines, indicating that extracellular H_2_O_2_ is required for P-AscH^−^-mediated modulation of EMT-associated transcriptional programs.

Given the close functional relationship between EMT activation and extracellular matrix remodeling [43,44], we next assessed whether extracellular H_2_O_2_ contributes to the regulation of invasion-associated proteolytic enzymes. ConsistenSct with the EMT gene expression data, catalase co-treatment restored the expression of both *MMP2* and *MMP9* to near-baseline levels in U251 and U87MG cells (Figure 7C,D). These findings indicate that extracellular H_2_O_2_ acts as a critical upstream determinant of P-AscH^−^-induced suppression of invasion-related gene expression.

Collectively, these results demonstrate that pharmacological ascorbate downregulates EMT-associated transcription factors and matrix-degrading enzymes predominantly through its pro-oxidant activity, thereby linking extracellular H_2_O_2_ generation to transcriptional control of glioblastoma invasiveness.

### 2.7. Extracellular H_2_O_2_ Drives P-AscH^−^-Induced Modulation of EMT-Associated Proteins in Glioblastoma

**Figure 8:**
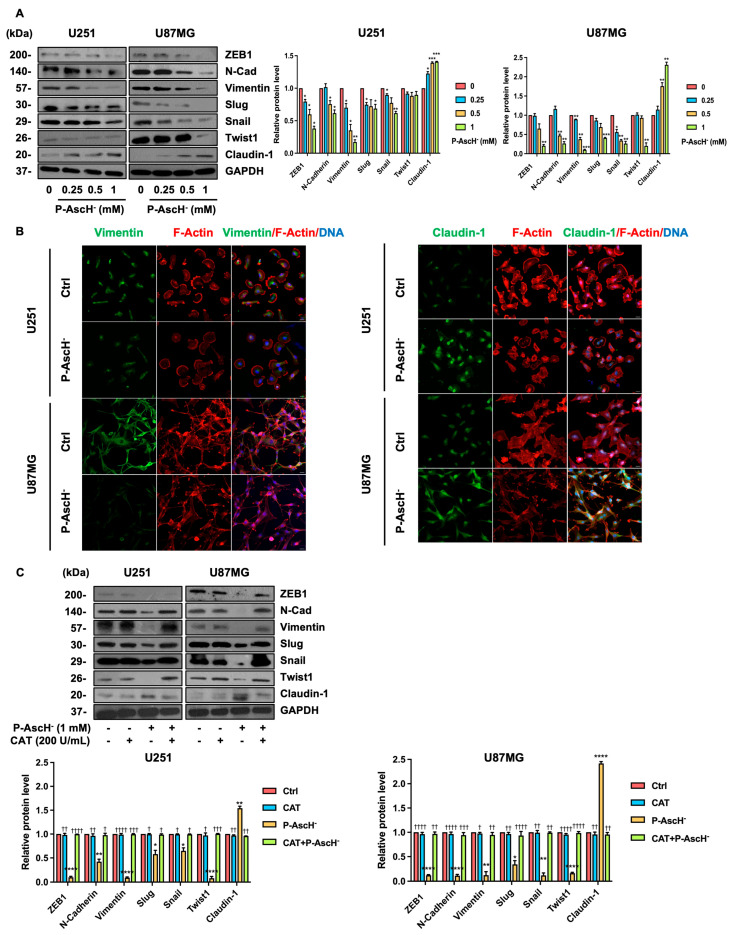
Pharmacological ascorbate suppresses EMT-associated protein expression through extracellular H_2_O_2_. (**A**) P-AscH^−^ reduces the protein abundance of mesenchymal markers (N-cadherin, Vimentin, Slug, Snail, Twist, and ZEB1) while concomitantly increasing expression of the epithelial marker Claudin-1. Cells were treated with P-AscH^−^ (0.25–1 mM) for 1 h, and protein levels were assessed by Western blotting at 6 h after treatment. Band intensities were quantified and normalized to the untreated control. (**B**) Immunofluorescence analysis confirms increased Claudin-1 expression and reduced Vimentin expression in both U251 and U87MG cells following P-AscH^−^ treatment. Cells were exposed to P-AscH^−^ (1 mM) for 1 h, and protein localization was visualized by immunofluorescence at 6 h after treatment. (**C**) Extracellular hydrogen peroxide generated by P-AscH^−^ is required for EMT protein modulation. Cells were treated with P-AscH^−^ (1 mM) in the presence or absence of CAT (200 U/mL) for 1 h, and EMT-associated protein expression was analyzed by Western blotting at 6 h after treatment. Western blot and immunofluorescence images are representative of three independent biological experiments. Quantitative data are presented as mean ± SEM (*n* = 3 biological replicates, each with three technical replicates). Ctrl, untreated control; CAT, catalase. Scale bar = 20 μm. *, *p* < 0.05; **, *p* < 0.01; ***, *p* < 0.001; ****, *p* < 0.0001 vs. Ctrl; †, *p* < 0.05; ††, *p* < 0.01; †††, *p* < 0.001; ††††, *p* < 0.0001 vs. P-AscH^−^ alone.

To determine whether the transcriptional changes in EMT-related genes induced by P-AscH^−^ translated into corresponding protein-level alterations, EMT marker expression was examined by Western blotting and immunofluorescence analysis. As shown in Figure 8A, P-AscH^−^ treatment resulted in a dose-dependent reduction in the protein levels of multiple mesenchymal markers, including ZEB1, N-cadherin, Vimentin, Slug, and Twist1, in both U251 and U87MG cells. In parallel, expression of the epithelial marker Claudin-1 was significantly increased. These protein-level changes were concordant with the transcriptional profiling results, confirming that P-AscH^−^ suppresses mesenchymal features while promoting epithelial characteristics in GBM cells.

To further visualize EMT modulation at the cellular level, immunofluorescence staining was performed. Vimentin and Claudin-1 were selected as representative mesenchymal and epithelial markers, respectively. Vimentin was chosen because it is a core intermediate filament protein that supports cytoskeletal remodeling, cell polarity, and mechanical flexibility during mesenchymal transition [45] and is closely linked to enhanced migratory and invasive capacity in glioblastoma cells [46]. In addition, Vimentin exhibited the most robust and consistent suppression among mesenchymal markers in our Western blot analyses (Figure 8B). Claudin-1 was selected as an epithelial marker due to its established role as a tight junction protein indicative of epithelial integrity and cell–cell adhesion [47]. Consistent with the immunoblotting results, P-AscH^−^ treatment markedly reduced vimentin staining, reflecting disruption of mesenchymal cytoskeletal organization, while concurrently restoring Claudin-1 localization at the plasma membrane (Figure 8B). Together, these changes visually corroborate the biochemical data and highlight a P-AscH^−^-induced shift away from mesenchymal characteristics toward a more epithelial-like cellular phenotype.

The contribution of extracellular H_2_O_2_ to these protein-level EMT changes was further assessed by catalase co-treatment. As shown in Figure 8C, catalase effectively prevented the P-AscH^−^-mediated reduction in mesenchymal proteins and abolished Claudin-1 induction, restoring EMT marker expression patterns to levels comparable to untreated controls. Together, these findings demonstrate that extracellular H_2_O_2_ is indispensable for P-AscH^−^-induced modulation of EMT-associated proteins and reinforce the role of its pro-oxidant activities in limiting glioblastoma invasiveness.

### 2.8. Pharmacological Ascorbate Attenuates mTOR Signaling and Cooperates with mTOR Inhibition to Suppress Glioblastoma Migration

**Figure 9:**
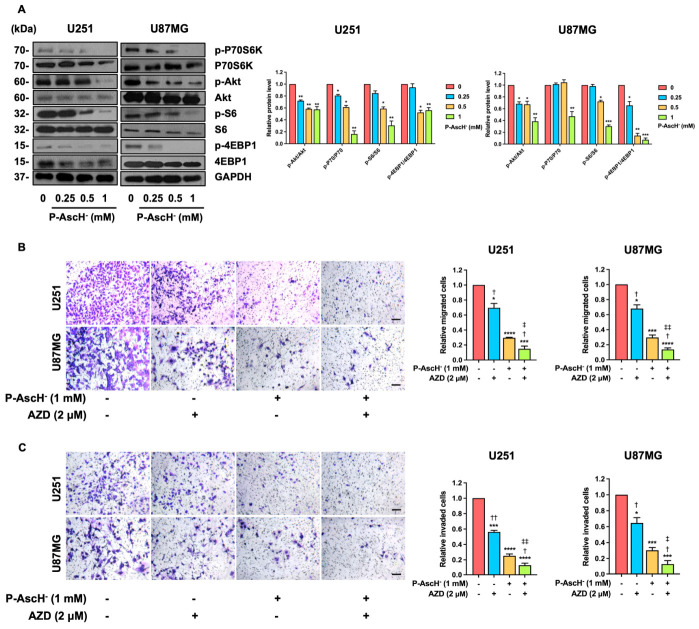
Pharmacological ascorbate attenuates mTOR signaling and enhances the inhibitory effects of dual mTOR blockade on glioblastoma cell migration and invasion. (**A**) P-AscH^−^ attenuates both mTORC1 and mTORC2 signaling in glioblastoma cells. Cells were treated with P-AscH^−^ (0.25–1 mM) for 1 h, and phosphorylation of canonical mTORC1 downstream targets (pThr389-p70S6K, pSer235/236-S6, and pSer65-4EBP1) and the mTORC2 readout pSer473-Akt was assessed by Western blotting at 6 h after treatment. Phosphorylated protein levels were normalized to their respective total protein levels and expressed relative to untreated controls. Representative immunoblots from three independent experiments are shown, with quantitative data presented as mean ± SEM (*n* = 3). (**B**,**C**) P-AscH^−^ enhances the anti-migratory effects of the dual mTORC1/2 inhibitor AZD8055. Cells were treated with P-AscH^−^ alone (1 mM; 1 h), AZD8055 alone (2 μM; 24 h), or the combination. For combination treatment, cells were pre-treated with AZD8055 for 24 h followed by P-AscH^−^ exposure for 1 h. Cell migration (**B**) and invasion (**C**) were evaluated using transwell-based assays. Data are expressed as the number of migrated or invaded cells relative to untreated controls. Representative microscopic images and quantification are derived from three independent experiments. Data are presented as mean ± SEM. Ctrl, untreated control. Scale bar = 50 μm. *, *p* < 0.05; **, *p* < 0.01; ***, *p* < 0.001; ****, *p* < 0.0001 vs. Ctrl; †, *p* < 0.05; ††, *p* < 0.01 vs. P-AscH^−^ alone; ‡, *p* < 0.05; ‡‡, *p* < 0.01 vs. AZD8055 alone.

mTOR signaling is a central regulator of glioblastoma cell migration, invasion, and EMT, and aberrant activation of this pathway is strongly associated with aggressive tumor behavior, poor patient prognosis, and therapeutic resistance [4]. Previous studies have demonstrated that genetic or pharmacological disruption of mTOR signaling attenuates glioma cell motility and suppresses mesenchymal-like phenotypes, underscoring the requirement of this axis for maintaining the invasive properties of GBM cells [48,49]. Consistent with these observations, Western blot analyses revealed that P-AscH^−^ induced a dose-dependent suppression of mTOR pathway activation, as evidenced by reduced phosphorylation of Akt, p70S6K, S6, and 4EBP1 relative to total protein levels (Figure 9A), identifying mTOR signaling as a downstream pathway modulated by P-AscH^−^ in glioblastoma therapy.

To assess whether inhibition of mTOR signaling functionally contributes to the anti-migratory effects of pharmacological ascorbate (P-AscH^−^), we next examined the impact of direct mTOR blockade on glioblastoma cell motility and its interaction with P-AscH^−^. mTOR signaling is mediated through two functionally distinct complexes, mTORC1 and mTORC2, which together regulate cytoskeletal organization, protein translation, and invasion-associated programs [50]. Accordingly, AZD8055, a well-characterized dual mTORC1/2 inhibitor [51], effectively suppresses Akt/mTOR signaling in glioblastoma cells (Figure S10A–C), was employed to interrogate the contribution of mTOR signaling to glioblastoma migration and invasion. In transwell-based assays, AZD8055 treatment alone significantly reduced both migration and invasion in U251 and U87MG cells, supporting a critical role for mTOR signaling in regulating glioblastoma motility (Figure 9B,C). Notably, combined treatment with P-AscH^−^ and AZD8055 produced a greater overall reduction in total motile cells than either agent alone (Figure 9B,C), indicating that pharmacological ascorbate enhances the inhibitory effects of mTOR blockade on glioblastoma cell motility.

**Figure 10:**
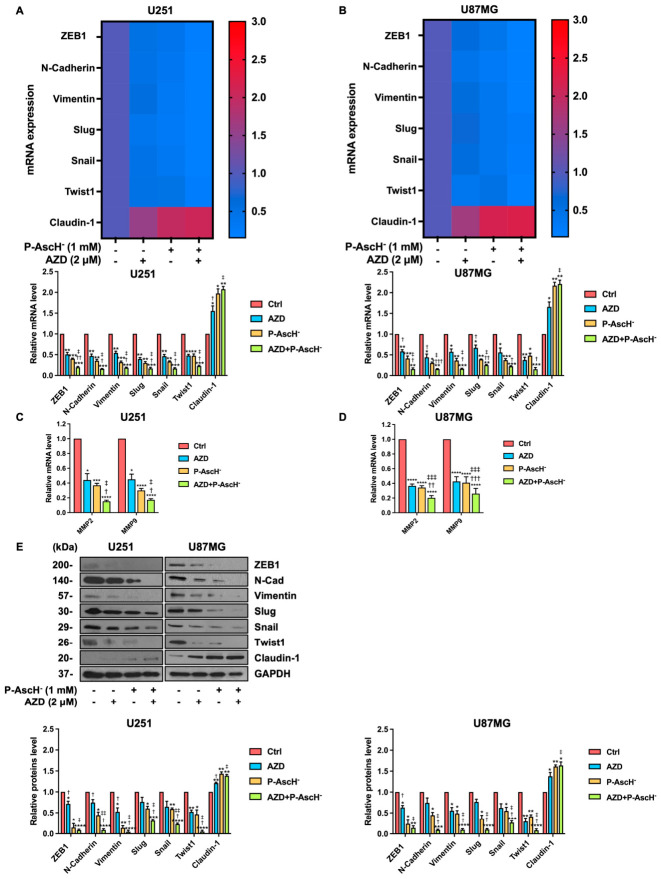
Pharmacological ascorbate enhances the inhibitory effects of AZD8055 on EMT- and invasion-associated gene and protein expression in glioblastoma cells. (**A**–**D**) P-AscH^−^ augments the suppression of EMT- and MMP-associated gene expression by the dual mTORC1/2 inhibitor AZD8055. Glioblastoma cells were treated according to the same experimental protocol described in Figure 9B,C. Total RNA was extracted at 6 h after treatment, and mRNA levels of EMT markers and MMPs were quantified by qRT-PCR. Transcript abundance is expressed relative to untreated controls. (**E**) P-AscH^−^ enhances AZD8055-mediated suppression of EMT-associated protein expression. Cells were treated using the same protocol as in Figure 9B,C, and protein expression was analyzed by Western blotting at 6 h after treatment. Protein levels were quantified relative to untreated controls. Representative immunoblots from three independent experiments are shown. Data are presented as mean ± SEM (*n* = 3 independent biological experiments, each with three technical replicates). Ctrl, untreated control. *, *p* < 0.05; **, *p* < 0.01; ***, *p* < 0.001; ****, *p* < 0.0001 vs. Ctrl; †, *p* < 0.05; ††, *p* < 0.01; †††, *p* < 0.001 vs. P-AscH^−^ alone; ‡, *p* < 0.05; ‡‡, *p* < 0.01; ‡‡‡, *p* < 0.001 vs. AZD8055 alone.

At the transcriptional level, qRT-PCR analyses showed that AZD8055 treatment alone significantly reduced the expression of EMT-associated genes, including *N-cadherin*, *Vimentin*, *Slug*, *Snail*, *Twist*, and *ZEB1*, while increasing the expression of the epithelial marker *Claudin-1* and decreasing the invasion-associated genes *MMP2* and *MMP9* in both U251 and U87MG cells (Figure 10A–D). These findings are consistent with an essential role for mTOR signaling in sustaining migratory and invasive programs in GBM. Co-treatment with P-AscH^−^ and AZD8055 resulted in a more pronounced repression of mesenchymal and invasion-associated transcripts than AZD8055 monotherapy (Figure 10A–D).

Protein-level analyses corroborated these transcriptional changes. AZD8055 reduced the abundance of multiple mesenchymal markers while concomitantly increasing Claudin-1 expression. Notably, combined treatment with P-AscH^−^ led to further suppression of mesenchymal proteins and resulted in an increased level of Claudin-1 compared with AZD8055 monotherapy (Figure 10E). Taken together, combined pharmacological ascorbate and mTOR blockade elicit coordinated suppression of EMT regulators at transcriptional and translational levels, consistent with strengthened attenuation of mesenchymal programs that govern glioblastoma cell motility.

Collectively, these findings demonstrate that pharmacological ascorbate suppresses glioblastoma migration and invasion, along with EMT-associated programs, at least in part, through attenuation of mTOR signaling. Moreover, the greater reduction in migratory behavior observed with combined P-AscH^−^ and mTOR inhibition supports the concept that pharmacological ascorbate can complement mTOR-targeted therapies by reinforcing suppression of mesenchymal, motility-, and invasion-associated pathways, providing a mechanistic rationale for its consideration as an adjuvant strategy in mTOR-directed glioblastoma treatment.

### 2.9. Pharmacological Ascorbate Suppresses Invasion and EMT Programs in a Patient-Derived Glioblastoma Model and Enhances the Effects of mTOR Inhibition

**Figure 11:**
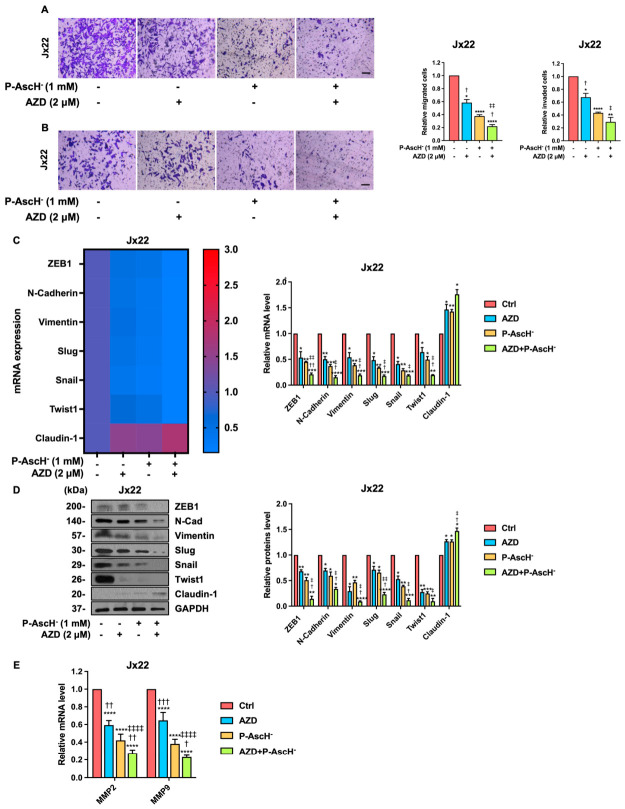
Pharmacological ascorbate enhances the anti-migratory, anti-invasive and EMT-suppressive effects of AZD8055 in a patient-derived glioblastoma model. (**A**,**B**) P-AscH^−^ enhances the inhibitory effects of AZD8055 on migration and invasion in the patient-derived glioblastoma model Jx22. Cells were treated according to the same experimental protocol described in Figure 9B,C. Migration was assessed using transwell assays (**A**), and invasion was evaluated using Matrigel^®^-coated transwell assays (**B**). Representative images from three independent experiments are shown. Quantification is presented as the number of migrated or invaded cells relative to untreated controls. (**C**–**E**) P-AscH^−^ reinforces AZD8055-mediated suppression of EMT- and invasion-associated molecular programs in Jx22 cells. Treatments were performed under the same conditions described in Figure 9B,C. EMT-associated (**C**) and matrix metalloproteinase (MMP)-associated (**E**) mRNA expression levels were quantified by qRT-PCR, while EMT-associated protein expression was evaluated by Western blotting (**D**). Transcript and protein expression levels are expressed relative to untreated controls. Representative immunoblots from three independent experiments are shown. Data are presented as mean ± SEM (*n* = 3 independent biological experiments, each with three technical replicates). Ctrl, untreated control. Scale bar = 50 μm. *, *p* < 0.05; **, *p* < 0.01; ***, *p* < 0.001; ****, *p* < 0.0001 vs. Ctrl; †, *p* < 0.05; ††, *p* < 0.01; †††, *p* < 0.001 vs. P-AscH^−^ alone; ‡, *p* < 0.05; ‡‡, *p* < 0.01; ‡‡‡‡, *p* < 0.0001 vs. AZD8055 alone.

To extend the relevance of our findings beyond established glioblastoma cell lines, we evaluated the effects of P-AscH^−^ in the patient-derived glioblastoma model Jx22. This model was selected because patient-derived lines retain key molecular, genetic, and phenotypic features of human glioblastoma, providing a clinically relevant platform for translational validation [52,53]. In transwell-based assays, P-AscH^−^ treatment significantly reduced the overall motility of Jx22 cells, as reflected by decreased migration and invasion compared with untreated controls (Figure 11A,B). These results extend our observations in established GBM cell lines and demonstrate that the anti-migratory and anti-invasive effects of pharmacological ascorbate are preserved in the patient-derived glioblastoma model.

Consistent with the role of mTOR signaling in regulating invasive behavior, treatment with the dual mTORC1/2 inhibitor AZD8055 also significantly suppressed migration and invasion in Jx22 cells (Figure 11A,B). Interestingly, combined treatment with P-AscH^−^ and AZD8055 further reduced both migrated and invaded cell populations compared with AZD8055 alone (Figure 11A,B). Notably, this pattern aligns with our findings in U87MG and U251 cells, supporting the potential utility of P-AscH^−^ in combination with mTOR-targeted strategies.

At the molecular level, analyses of Jx22 cells revealed that P-AscH^−^ reduced expression of a broad panel of mesenchymal markers (N-cadherin, Vimentin, Slug, Snail, Twist, and ZEB1) while increasing expression of the epithelial marker Claudin-1 at both mRNA (Figure 11C) and protein levels (Figure 11D). In parallel, P-AscH^−^ significantly suppressed *MMP2* and *MMP9* transcript levels, supporting inhibition of EMT-associated and invasion-related gene programs in a patient-derived GBM model (Figure 11E). AZD8055 produced a comparable molecular profile, characterized by coordinated downregulation of mesenchymal markers, induction of epithelial features, and repression of *MMP2* and *MMP9* mRNA expression (Figure 11C–E), reinforcing the central role of mTOR signaling in sustaining mesenchymal and invasive phenotypes in glioblastoma.

Importantly, co-treatment with P-AscH^−^ and AZD8055 resulted in a more pronounced and consistent suppression across the mesenchymal marker panel than either treatment alone, evident at both transcriptional and protein levels (Figure 11C,D). In addition, the combination treatment increased Claudin-1 expression, with a trend toward elevated mRNA levels and a significant increase at the protein level compared with AZD8055 alone (Figure 11C,D). This shift further supports reinforcement of an epithelial-like phenotype in patient-derived GBM cells. Further suppression of *MMP2* and *MMP9* transcripts by the combination was also observed in this model (Figure 11E), consistent with findings in established GBM cell lines. Together, these findings indicate that pharmacological ascorbate enhances the ability of mTOR inhibition to suppress mesenchymal identity and invasion-associated gene expression in patient-derived glioblastoma cells, highlighting the translational potential of combining P-AscH^−^ with mTOR-targeted therapies for limiting GBM invasiveness.

## 3. Discussion

Glioblastoma remains one of the most lethal human malignancies, with diffuse infiltration into the surrounding brain parenchyma representing its defining clinical challenge. Even after maximal surgical resection, residual tumor cells disseminate along white-matter tracts and perivascular niches, establishing microscopic reservoirs that inevitably fuel recurrence [1,2]. This extraordinary invasive capacity is not a passive property but an actively regulated program, in which GBM cells dynamically interconvert among transcriptional states (pro-neural, mesenchymal, stem-like, and hybrid) in response to microenvironmental and therapeutic stress [54,55]. Among these states, the mesenchymal program is particularly associated with enhanced migratory capacity, extracellular matrix remodeling, and resistance to oxidative injury [13,56], collectively enabling GBM cells to survive and infiltrate under hostile conditions. Effective therapeutic strategies must therefore move beyond direct cytotoxicity to target the regulatory networks governing this invasive, phenotypically plastic behavior.

Within this framework, the present study investigates pharmacological ascorbate (P-AscH^−^) as a candidate modulator of GBM invasiveness. Using comparative transcriptomic profiling of H4, LN229, and U87MG cells, we first established a molecular basis for model selection, identifying in U87MG a coordinated transcriptional program that integrates mitochondrial metabolic activation, cytoskeletal remodeling, and robust enrichment of epithelial-to-mesenchymal transition (EMT)-associated pathways (Figure 1 and Figure 2), features that collectively define a highly aggressive GBM phenotype. Notably, U87MG exhibited enhanced oxidative phosphorylation and ROS-related pathway enrichment relative to the other lines, consistent with emerging evidence that metabolically plastic, aggressive GBM subpopulations can exploit mitochondrial activity to support energy-intensive processes such as directional migration and cytoskeletal reorganization [57,58,59]. Direct comparison between U87MG and LN229 further revealed enrichment of mitotic spindle organization and DNA replication pathways uniquely in U87MG, indicating that its aggressive phenotype integrates proliferative acceleration with enhanced motility in a hybrid malignant state supported by shared microtubule dynamics [60,61,62,63,64,65,66]. This bioinformatics-driven characterization provided a mechanistically grounded rationale for selecting U87MG as the primary model for downstream mechanistic interrogation, while U251 was incorporated to confirm that key findings were not restricted to a single genetic background.

Building on the central role of cell motility in glioblastoma dissemination, our study demonstrates that P-AscH^−^ markedly suppresses both migratory and invasive behavior in U87MG and U251 cells, as assessed by complementary wound-healing and transwell-based assays (Figure 4). Importantly, all experiments were conducted using concentrations of P-AscH^−^ that were defined as sub-cytotoxic based on cell viability and proliferation analyses (Figure 3). This experimental design minimizes confounding effects from overt cytotoxicity or growth arrest and indicates that the observed reductions in migration and invasion reflect intrinsic inhibitory effects on motility programs rather than secondary consequences of cell loss. Notably, although sub-cytotoxic P-AscH^−^ exposure did not significantly alter short-term viability or proliferative rates, it produced a pronounced suppression of clonogenic survival (Figure S11). This effect was manifested as a significant reduction in colony-forming efficiency, revealing a compromised capacity for long-term proliferation and self-renewal following transient P-AscH^−^ treatment. The divergence between short-term viability assays and long-term clonogenic outcomes underscores an important biological distinction: brief exposure to pharmacological ascorbate can induce durable cellular changes that impair the ability of GBM cells to re-establish proliferative colonies, even in the absence of immediate cytotoxic effects. Together, these findings suggest that P-AscH^−^ exerts sustained inhibitory pressure on GBM cell fitness, extending beyond acute growth suppression to affect long-term tumorigenic potential.

A mechanistic basis for this anti-invasive activity was established through coordinated suppression of EMT-associated programs. P-AscH^−^ induced downregulation of canonical mesenchymal markers, specifically N-cadherin, vimentin, Slug, Snail, Twist, and ZEB1, together with induction of the epithelial marker Claudin-1, at both the transcriptional (Figure 5A,B) and protein levels (Figure 8A,B). These molecular changes were accompanied by significant suppression of *MMP2* and *MMP9* transcripts (Figure 5C,D), linking EMT inhibition to a concurrent reduction in extracellular matrix-degrading capacity. Together, these findings position P-AscH^−^ as a suppressor of the full cytoskeletal and proteolytic machinery required for GBM cell infiltration. Importantly, these anti-invasive effects are consistent with prior reports in pancreatic [21,67] and uterine cancers [68], where P-AscH^−^ similarly disrupted EMT programs and suppressed MMP expression, and the present data extend those observations to GBM, a tumor characterized by exceptional phenotypic plasticity. This conservation of effect supports the potential of P-AscH^−^ as a broad anti-invasive strategy across aggressive cancer types.

Mechanistically, pharmacological ascorbate is widely proposed to exert its anticancer effects through a pro-oxidant mechanism driven by the extracellular generation of H_2_O_2_ [18,19,24,67,69,70,71]. We provide direct evidence that this oxidant species is the critical upstream determinant of P-AscH^−^-mediated invasion suppression in GBM: enzymatic scavenging of extracellular H_2_O_2_ by extracellular catalase completely abolished the inhibitory effects on GBM cell motility (Figure 6). Crucially, catalase co-treatment also reversed the P-AscH^−^-induced downregulation of mesenchymal markers (N-cadherin, Vimentin, Slug, Snail, Twist, and ZEB1) and the induction of the epithelial marker Claudin-1 at both transcriptional and protein levels (Figure 7A,B and Figure 8C), linking redox stress directly to modulation of EMT-associated programs. In parallel, extracellular H_2_O_2_ was required for suppression of *MMP2* and *MMP9* transcripts (Figure 7C,D), further connecting oxidative signaling to reduced extracellular matrix-degrading capacity. These results identify extracellular H_2_O_2_ as a central redox signal that coordinates inhibition of migration, invasion, EMT-associated transcriptional programs, and proteolytic remodeling in a unified mechanistic cascade. To our knowledge, this represents the first mechanistic evidence in GBM directly linking pharmacological ascorbate-derived H_2_O_2_ to suppression of invasive behavior through integrated regulation of EMT and MMP expression, thereby expanding the functional relevance of redox-based therapy in GBM well beyond tumor-selective cytotoxicity.

The mTOR signaling pathway is a central regulator of GBM cell growth, migration, and invasion [72,73], and its intersection with P-AscH^−^ activity was examined at both the signaling and functional levels. P-AscH^−^ broadly attenuated mTOR pathway activity, as evidenced by reduced phosphorylation of mTORC1 downstream effectors (p70S6K, S6, and 4EBP1) and of Akt, a key readout of mTORC2 activity, in both U251 and U87MG models (Figure 9A). These findings indicate that P-AscH^−^ broadly attenuates mTOR signaling rather than selectively targeting a single complex. Importantly, this dual inhibition of mTORC1 and mTORC2 is concordant with our previous report [24], in which substantially higher concentrations of P-AscH^−^ (8 mM; approximately 8–32-fold greater than those used here) similarly suppressed both mTOR complexes in glioma cells. However, under those relatively high-dose conditions, mTOR inhibition was accompanied by a global reduction in total and phosphorylated protein levels, consistent with overt cytotoxic stress. In contrast, the present study demonstrates that attenuation of mTOR signaling can be achieved at sub-cytotoxic concentrations of P-AscH^−^, without detectable loss of total protein abundance. This distinction suggests that suppression of the mTOR pathway by pharmacological ascorbate is not merely a downstream consequence of generalized protein degradation or cell death but can occur as a regulated signaling response under physiologically relevant conditions.

Building on this dual inhibitory effect on mTORC1 and mTORC2 signaling, we next examined whether pharmacological ascorbate could enhance the functional impact of direct mTOR blockade. P-AscH^−^ increased the anti-migratory and anti-invasive effects of the dual mTORC1/2 inhibitor AZD8055 (Figure 9B,C), with the combination producing more pronounced repression of mesenchymal marker expression at both mRNA and protein levels (Figure 10A,B,E) and further suppression of *MMP2* and *MMP9* transcripts (Figure 10C,D) compared to either agent alone. These coordinated molecular changes provide a mechanistic basis for the observed reduction in motile and invasive cell populations, and collectively support a rationale for combining P-AscH^−^ with mTOR-directed therapy to reinforce suppression of mesenchymal and motility-associated signaling in GBM.

To strengthen the translational relevance of these findings, we validated key observations in a patient-derived glioblastoma model (Jx22 cells). Patient-derived models preserve the genetic, molecular, and phenotypic features of original patient tumors, including intertumoral heterogeneity and invasive behavior, and are therefore considered more predictive of clinical responses than long-term cultured cell lines [52,53]. P-AscH^−^ significantly impaired the migratory and invasive capacity of Jx22 cells (Figure 11A,B), with accompanying coordinated suppression of mesenchymal markers and induction of the epithelial marker Claudin-1 at both mRNA and protein levels (Figure 11C,D), together with downregulation of *MMP2* and *MMP9* transcripts (Figure 11E). These molecular patterns closely mirror findings in established cell lines. Combination treatment with AZD8055 produced further enhancement of these effects in the patient-derived model (Figure 11), reinforcing the potential clinical value of integrating P-AscH^−^ with mTOR-targeted therapy to suppress invasive and phenotypically plastic GBM cell states. The consistency of these effects across both established and patient-derived models underscores the mechanistic robustness of the P-AscH^−^ anti-invasive program.

It is also important to recognize that, beyond its established pro-oxidant activity, ascorbate serves as an essential cofactor for a broad family of 2-oxoglutarate-dependent dioxygenases, several of which regulate pathways closely related to those examined in the present study. These include prolyl hydroxylase domain-containing enzymes (PHDs), Ten-Eleven Translocation (TET) methylcytosine dioxygenases, and Jumonji C-domain (JMJD) histone demethylases [17]. Thus, an additional mechanistic layer may contribute to the observed anti-invasive effects of pharmacological ascorbate. Specifically, ascorbate may restore or enhance PHD activity, promoting HIF-1α hydroxylation and subsequent proteasomal degradation [74,75]. Reduced HIF-1α signaling could consequently attenuate transcriptional programs linked to mesenchymal transition and invasion, including the induction of MMPs and other pro-invasive mediators [76]. In parallel, TET and JMJD enzymes may influence cellular phenotype through epigenetic remodeling by facilitating DNA and histone demethylation at loci governing epithelial identity and invasion-associated pathways [77,78,79]. Importantly, these dioxygenase-dependent mechanisms are unlikely to function independently of the extracellular H_2_O_2_-mediated pro-oxidant effects identified in the present study. Rather, they may act in concert as complementary mechanisms through which pharmacological ascorbate reinforces EMT suppression and restrains invasive behavior. Further studies will be required to delineate the relative contribution and interplay of these interconnected pathways in mediating the anti-invasive phenotype observed here.

Several limitations of the present study should be acknowledged. Although the inclusion of patient-derived GBM cells improves translational relevance, the majority of experiments were conducted under in vitro conditions, which cannot fully capture the complexity of the brain tumor microenvironment. GBM progression is influenced not only by tumor cell-intrinsic mechanisms but also by dynamic interactions among stromal, immune, and vascular components that collectively regulate invasive behavior and local redox homeostasis. Consequently, the anti-invasive effects of pharmacological ascorbate observed in simplified culture systems may differ within the intact tumor ecosystem.

A major contributor to this complexity is the diverse cellular composition of the GBM microenvironment. Reactive astrocytes at the invasive tumor margin secrete factors that promote EMT-like programs and cytoskeletal remodeling [80,81] while simultaneously enhancing antioxidant buffering through glutathione synthesis and the release of cysteine precursors [82,83]. Such adaptations may attenuate extracellular peroxide accumulation and reduce the effective oxidative H_2_O_2_ flux generated by P-AscH^−^. Likewise, tumor-associated macrophages and microglia, which frequently adopt an immunosuppressive M2-like phenotype, contribute to mesenchymal transition and tumor invasion through cytokine signaling [84]. These cells also express elevated levels of peroxide-detoxifying enzymes, including catalase and glutathione peroxidase [85], potentially restricting the magnitude and duration of P-AscH^−^-mediated oxidative signaling.

Vascular and metabolic factors add an additional layer of complexity. The abnormal vasculature of GBM generates hypoxic gradients that stabilize HIF-1α and promote extracellular matrix remodeling, mesenchymal transition, and invasive behavior [4,86], processes that may influence the response to P-AscH^−^ in ways not adequately modeled under normoxic culture conditions. Furthermore, the blood–brain barrier, regional variations in catalytic metals, antioxidant reserves, and peroxide-scavenging enzymes may collectively alter the distribution and redox activity of pharmacological ascorbate in vivo [87].

Collectively, these limitations emphasize the importance of validating the present findings in physiologically relevant models. Future studies employing orthotopic patient-derived xenograft models together with redox-sensitive imaging and integrated metabolic analyses will be important for defining how tumor microenvironment-derived cellular and biochemical factors shape oxidative signaling and ultimately influence the anti-invasive efficacy of P-AscH^−^ within the intact brain.

An additional consideration relates to the physicochemical environment of conventional cell culture systems used in pharmacological ascorbate studies. Extracellular H_2_O_2_ generation by P-AscH^−^ depends on catalytic redox-active transition metals that facilitate electron transfer reactions and oxygen reduction [17]. In the present study, treatments were performed under serum-free conditions to minimize variability associated with serum-derived iron. Under these conditions, catalytic metal availability would primarily arise from defined transition-metal components within media formulations (e.g., ferric nitrate, ferrous sulfate, copper salts) and potential adventitious metal contaminants introduced during media preparation. Although present at low concentrations, these metal pools are sufficient to catalyze ascorbate oxidation and sustain extracellular H_2_O_2_ generation at biologically relevant fluxes [88,89]. Importantly, conventional culture systems differ substantially from physiological environments, where iron availability is tightly regulated by metal-binding proteins and intracellular homeostatic mechanisms [90]. Among these regulatory systems, transferrin serves as the principal extracellular iron transport protein and has also been proposed as a potential source of catalytic iron capable of supporting ascorbate oxidation under specific conditions [91,92]. This observation suggests that the biologically active catalytic metal pool may extend beyond freely dissolved transition metal ions alone. Nevertheless, compared with tightly controlled physiological systems, in vitro culture conditions may provide a more permissive environment for metal-catalyzed oxidation reactions and therefore may not fully recapitulate endogenous redox homeostasis. Despite these limitations, the translational relevance of P-AscH^−^ does not appear to be substantially diminished. Landmark studies by Chen’s research team demonstrated that H_2_O_2_ generated following pharmacological ascorbate exposure in cell culture systems was quantitatively comparable to extracellular levels observed in animal models, while pharmacokinetic study showed that intravenous administration in humans achieved millimolar plasma concentrations of ascorbate comparable to preclinical settings [69,70,93]. Together, these findings support the biological and translational relevance of the pro-oxidant mechanism of P-AscH^−^. Future studies employing orthotopic GBM models with direct quantification of H_2_O_2_ flux within tumor interstitial fluid and, where accessible, cerebrospinal fluid, will be important to fully define the oxidant dynamics achievable at the tumor site in vivo.

Pharmacological ascorbate is currently under active clinical investigation across multiple cancer types, with early-phase studies demonstrating favorable tolerability and preliminary efficacy signals, particularly in combination with standard therapies [19,21,26,94,95,96]. In glioblastoma, this momentum is reflected in ongoing clinical trials, including a Phase I study (NCT04900792) and a Phase II trial (NCT02344355). A persistent challenge in translating these findings into clinical integration, however, has been the absence of mechanistic frameworks that extend beyond direct tumor cytotoxicity. The present study addresses this gap directly. By demonstrating that P-AscH^−^ suppresses GBM migration, EMT-associated transcriptional programs, and mTOR-dependent signaling at sub-cytotoxic concentrations, and that extracellular H_2_O_2_ coordinates these effects in an integrated mechanistic cascade, our findings highlight a previously underappreciated dimension of pharmacological ascorbate activity. In glioblastoma, where diffuse infiltration rather than uncontrolled proliferation alone drives recurrence and therapeutic failure [97], an agent capable of targeting invasive and phenotypically plastic tumor states offers a distinct and complementary therapeutic rationale. These findings therefore provide a biologically grounded basis for incorporating pharmacological ascorbate into future clinical strategies aimed at limiting glioblastoma invasiveness and improving treatment durability.

## 4. Materials and Methods

### 4.1. Chemicals and Reagents

A complete list of chemicals and reagents, including manufacturer information and catalog numbers, is summarized in Supplementary Table S1.

### 4.2. Cell Cultures

The human glioblastoma cell line H4 (CVCL_1239), LN229 (CVCL_0393), and U87MG (CVCL_0022) were obtained from the American Type Culture Collection (ATCC; Manassas, VA, USA). The human glioma cell lines U251 (CVCL_0021) were originally provided by Dr. Darell D. Bigner (Duke University, Durham, NC, USA). The patient-derived glioblastoma cells Jx22 was kindly provided by Dr. Jann Sarkaria (Mayo Clinic, Rochester, MN, USA) [53].

H4, LN229, and U87MG cells were cultured in Dulbecco’s Modified Eagle Medium (DMEM), while U251 and Jx22 cells were maintained in DMEM/F12 medium. All culture media were supplemented with 10% fetal bovine serum (FBS), 100 U/mL penicillin, and 100 μg/mL streptomycin. Cells were maintained at 37 °C in a humidified incubator under an atmosphere of 95% air and 5% CO_2_. Routine screening for mycoplasma contamination was performed using the Universal Mycoplasma Detection Kit (ATCC; Manassas, VA, USA).

### 4.3. RNA Extraction

Human glioma cell lines H4, LN229, and U87MG were cultured in 10 cm tissue culture dishes under standard conditions until approximately 90% confluency. Cells were harvested for total RNA extraction using the RNeasy Mini Kit (Qiagen, Hilden, Germany) according to the manufacturer’s instructions, including on-column DNase digestion to remove genomic DNA contamination. Total RNA concentration and purity were assessed spectrophotometrically, and RNA integrity was evaluated using the RNA Integrity Number (RIN). Only samples with RIN > 7.0 were used for downstream library preparation. At least 3 μg of total RNA per sample was dried under vacuum using GenTegra-RNA stabilization tubes (GenTegra^®^, Pleasanton, CA, USA) prior to sequencing library preparation. All experiments were performed with three independent biological replicates per cell line.

### 4.4. RNA Sequencing and Transcriptomic Data Analysis

RNA-seq libraries were prepared and sequenced by BMKGENE (Biomarker Technologies, Münster, Germany). Paired-end sequencing (2 × 150 bp) was performed on the Illumina PE150 platform (Illumina, San Diego, CA, USA). Raw sequencing reads were processed and aligned using the BMKGENE standard RNA-seq analysis pipeline. Transcript expression levels were quantified as fragments per kilobase of transcript per million mapped reads (FPKM). Principal component analysis (PCA) was conducted to assess global transcriptomic variation across samples. PCA plots were generated using BioJupies [98]. For downstream analyses, gene expression values were transformed to log_2_(FPKM + 1). Pairwise comparisons were performed between U87MG *v.s.* H4 and U87MG *v.s.* LN229. Log_2_ fold change (log_2_FC) values were calculated as:

log_2_ (FPKM + 1)*_U87MG_* − log_2_(FPKM + 1)*_H4_* and log_2_(FPKM + 1)*_U87MG_* − log_2_(FPKM + 1)*_LN229_*

Genes with |log_2_FC| > 1.5 were classified as differentially expressed genes (DEGs). Volcano plots displaying log_2_FC values were generated using GraphPad Prism (version 10.6.1; GraphPad Software, Boston, MA, USA). The raw datasets corresponding to each analysis are provided in Supplementary Tables S4–S6.

### 4.5. Gene Set and Pathway Enrichment Analysis

Cancer hallmark gene sets were obtained from the Molecular Signatures Database (MSigDB; Broad Institute of MIT and Harvard, Cambridge, MA, USA). Hierarchically clustered heatmaps were generated using Clustergrammer [99] and visualized in GraphPad Prism. Functional enrichment analysis of upregulated and downregulated DEGs was performed using DAVID Bioinformatics Resources (DAVID Knowledgebase v2024q4) [100]. Gene Ontology (GO) categories analyzed included Biological Process (GOBP), Cellular Component (GOCC), and Molecular Function (GOMF). The GO terms with a false discovery rate (FDR) < 0.05 were considered statistically significant. The top enriched unique terms were selected for visualization. Enrichment results were plotted as –log_10_(*p*-value) and color-coded according to fold enrichment values. Genes associated with epithelial–mesenchymal transition (EMT) were curated from published datasets [101]. Enrichment analyses of EMT-related gene subsets were performed using DAVID (v2024q4), following the same statistical criteria (FDR < 0.05). Fold enrichment values were plotted, with –log_10_(*p*-value) indicated by color scaling.

### 4.6. Treatment with P-AscH^−^

Preparation of ascorbate stock solutions and pharmacological ascorbate (P-AscH^−^) treatments were performed following established protocols developed by Wagner and Buettner [102]. Briefly, a 1.0 M L-ascorbic acid stock solution (pH 7.0) was prepared under an inert nitrogen atmosphere to minimize oxidative degradation. Ascorbate concentration was verified spectrophotometrically using a Cary 60 UV/Vis spectrophotometer (Agilent Technologies, Santa Clara, CA, USA) at 265 nm, applying a molar extinction coefficient (ε) of 14,500 M^−1^·cm^−1^. Stock solutions were stored in borosilicate glassware with minimal headspace to further limit oxidation.

Because extracellular hydrogen peroxide generation from P-AscH^−^ is influenced by medium composition and pH [102], cells were washed with phosphate-buffered saline and incubated in serum-free medium immediately prior to treatment. Cells were then exposed to P-AscH^−^ at 37 °C for 1 h, after which the treatment medium was removed and replaced with complete growth medium. Cells were subsequently processed for the indicated downstream assays.

### 4.7. Treatment with Catalase

To evaluate the contribution of extracellular hydrogen peroxide (H_2_O_2_) to the molecular effects of pharmacological ascorbate (P-AscH^−^), cells were co-treated with catalase during P-AscH^−^ exposure. Stock solutions were prepared in ultrapure water, sterilized using a 0.22 μm filter, and stored at 4 °C. Catalase was applied at a final concentration of 200 U/mL immediately before P-AscH^−^ exposure. This concentration has been previously validated to efficiently neutralize extracellular H_2_O_2_ [20,22,24].

### 4.8. Treatment with AZD8055

AZD8055 stock solutions were prepared in dimethyl sulfoxide (DMSO) and stored at −20 °C. Cells were treated with AZD8055 according to the specified experimental design. Control cells received an equivalent volume of DMSO as a vehicle control. The final DMSO concentration was maintained at 0.5% (*v*/*v*) in all treatment conditions to minimize solvent-related effects.

### 4.9. MTT Viability Assay

Cells were seeded into 96-well plates at a density of 1.0 × 10^4^ cells/well and incubated for 24 h to allow attachment. Following treatment, cells were washed with PBS and incubated with MTT solution (1 mg/mL in PBS; 100 μL/well) for 4 h at 37 °C in the dark. The MTT solution was then removed, and the resulting formazan crystals were solubilized in 200 μL DMSO. Absorbance was measured at 570 nm using a CLARIOStar microplate reader (BMG Labtech, Ortenberg, Germany). Cellular metabolic activity was expressed as a percentage relative to untreated controls and used as an indirect indicator of relative cell viability.

### 4.10. Proliferation Assay

Cells were seeded at a low density (5 × 10^3^ cells/well) in 96-well plates to permit progressive cell expansion throughout the experimental period and treated with varying concentrations of P-AscH^−^. Following treatment, cells were maintained in complete medium for 24, 48, and 72 h. At each time point, cellular metabolic activity was evaluated using the MTT assay.

To estimate relative changes in cell proliferation over time, optical density (OD) values obtained at 48 and 72 h were normalized to the corresponding OD value at 24 h for the same treatment group, which served as an internal baseline. Under these low-density culture conditions, temporal increases in MTT signal were used as an indirect surrogate measure of cell population expansion [103,104,105]. Data are presented as fold change relative to the 24 h baseline.

### 4.11. Wound Healing Assay

Cells were seeded at a density of 1.0 × 10^4^ cells per well in 96-well plates and cultured until a uniform confluent monolayer was established. Linear scratch wounds were generated using a sterile 10 μL pipette tip. Detached cells and debris were gently removed by washing with phosphate-buffered saline (PBS), after which cells were exposed to the indicated treatments in fresh medium.

Phase-contrast images were acquired at the specified time points using an inverted microscope (Zeiss PrimoVert, Jena, Germany). To ensure consistent imaging of the same wound region over time, reference positions on the culture plate were used during image acquisition. Wound areas were quantified using ImageJ software (version 1.54p; National Institutes of Health, Bethesda, MD, USA). For each image, wound width was measured at three predefined positions (top, middle, and bottom of the wound area), and the mean value was calculated for each field. For each well, the wound area at each time point was normalized to the corresponding wound area at 0 h, which was defined as 1. Data are presented as the relative remaining wound area over time, providing a quantitative measure of collective cell migratory capacity.

### 4.12. Transwell Migration and Invasion Assay

Cells were seeded in 6-well plates at a density of 1.0 × 10^5^ cells per well and allowed to adhere for 24 h prior to treatment. Following the indicated treatments, cells were harvested, resuspended in serum-free medium, and used for migration and invasion assays.

For migration assays, 2.5 × 10^4^ cells suspended in 300 μL of serum-free medium were seeded into the upper chambers of 24-well Transwell inserts. The lower chambers were filled with 700 μL of complete medium containing 10% FBS as a chemoattractant. After incubation for 24 h at 37 °C, non-migrated cells on the upper surface of the membrane were gently removed using a cotton swab. Cells that migrated to the lower surface were fixed with 3.7% paraformaldehyde, permeabilized with 100% methanol at −20 °C, and stained with 0.1% crystal violet. Migrated cells were imaged and quantified by counting five randomly selected fields per insert using a phase-contrast microscope (Zeiss PrimoVert, Jena, Germany).

For invasion assays, Transwell inserts were precoated with 100 μL of Matrigel^®^ (50 μg/mL diluted in serum-free medium) and incubated overnight at 37 °C to allow gel polymerization. Cells (2.5 × 10^4^ cells in 200 μL serum-free medium) were then seeded into the Matrigel-coated upper chambers, with 10% FBS-containing medium added to the lower chambers. After 24 h of incubation, invaded cells were fixed, permeabilized, stained, and quantified using the same procedure described for the migration assays.

### 4.13. RNA Extraction and Quantitative Real-Time Polymerase Chain Reaction (qRT–PCR)

Cells were seeded at a density of 3.0 × 10^5^ cells per well in 6-well plates and cultured under standard conditions for 24 h. Following indicated treatments, total RNA was isolated using GENEzol reagent. One microgram of RNA was reverse-transcribed into cDNA with SuperScript™ III Reverse Transcriptase(Invitrogen, Carlsbad, CA, USA). qRT-PCR was performed using SensiFAST™ SYBR^®^ NO-ROX Kit (Bioline London, UK) on a StepOnePlus Real-Time PCR system (Applied Biosystems, CA, USA). Cycling conditions were: 95 °C for 10 min, followed by 45 cycles of 95 °C for 30 s and 60 °C for 30 s. Relative mRNA levels were normalized to GAPDH and calculated using the 2^−ΔΔCt^ method [106]. Primer sequences are provided in Supplementary Table S2.

### 4.14. Western Blot Assay

To evaluate treatment-induced alterations in protein expression and signaling, GBM cells were plated at a density of 3.0 × 10^5^ cells per well in 6-well plates and allowed to adhere for 24 h prior to the indicated treatments. Following treatments, cells were lysed in RIPA buffer supplemented with protease and phosphatase inhibitors. Protein concentrations were determined using the Pierce BCA Protein Assay Kit (Thermo Fisher Scientific, Waltham, MA, USA). Equal protein amounts were separated by SDS-PAGE and transferred onto PVDF membranes. Membranes were blocked with BlockPro™ protein-free blocking buffer (Visual Protein Biotechnology, Taipei, Taiwan) for 5 min, followed by overnight incubation at 4 °C with primary antibodies. After washing, membranes were incubated with HRP-conjugated secondary antibodies for 1 h at room temperature. Signals were detected using Immobilon HRP chemiluminescent substrate, and band intensities were quantified with ImageJ. GAPDH served as a loading control. Antibody details are provided in Supplementary Table S3, and full-length blots are presented in Supplementary Figures S1–S7.

### 4.15. Immunofluorescence Assay

For immunofluorescence analysis, cells were seeded at a density of 2.0 × 10^4^ cells per well onto round glass coverslips (Menzel Gläser, Braunschweig, Germany) placed in 24-well plates and allowed to adhere for 24 h prior to the indicated treatments. Following treatment, cells were fixed using either cold methanol (−20 °C, 5 min) or 3.7% paraformaldehyde/glutaraldehyde (37 °C, 15 min), depending on the fixation requirements of the primary antibody. Cells were subsequently permeabilized with 0.1% Triton X-100 and blocked using BlockPro™ blocking buffer.

Coverslips were incubated overnight at 4 °C with the indicated primary antibodies (1:1000 dilution), followed by incubation with Alexa Fluor™ (Thermo Fisher Scientific, Waltham, MA, USA) 488-conjugated secondary antibodies (1:2000 dilution) for 1 h at room temperature. Nuclear counterstaining was performed using 4′,6-diamidino-2-phenylindole (DAPI). Coverslips were mounted with FluorSave™ (Calbiochem, Darmstadt, Germany) mounting medium.

Images were acquired using a Zeiss LSM 990 confocal microscope equipped with Airyscan (Zeiss, Jena, Germany) using a 20× objective. All immunofluorescence experiments were performed across three independent biological replicates.

### 4.16. Statistics

Data are presented as mean ± standard error of the mean (SEM) from at least three independent experiments. Statistical analyses were performed using GraphPad Prism. One-way analysis of variance (ANOVA) followed by Tukey’s post hoc test was used to assess statistical significance.

## 5. Conclusions

This study establishes a mechanistic framework in which pharmacological ascorbate restrains glioblastoma invasiveness through extracellular redox stress that converges on EMT-associated transcriptional programs and mTOR signaling. By generating extracellular H_2_O_2_, P-AscH^−^ suppresses mesenchymal identity, limits matrix-degrading capacity, and attenuates motility-associated signaling networks. These effects were observed at sub-cytotoxic concentrations and replicated across both established and patient-derived GBM models. Pharmacological ascorbate also reinforced the anti-migratory impact of mTOR inhibition, suggesting a complementary interaction between redox modulation and pathway-directed therapy. While these findings are derived from in vitro experimental systems and await validation in more complex tumor microenvironment models and in vivo settings, they nonetheless extend the conceptual role of pharmacological ascorbate beyond cytotoxicity toward modulation of invasive and plastic tumor cell states. These results provide a biologically grounded rationale for continued investigation of pharmacological ascorbate as an adjuvant strategy in glioblastoma and, potentially, other highly invasive cancers.

## Data Availability

The original contributions presented in this study are included in the article/Supplementary Material. Further inquiries can be directed to the corresponding author.

## Supplementary Materials

The following supporting information can be downloaded at https://www.mdpi.com/article/10.3390/ijms27114964/s1.
