# Supplementary material for: Pharmacological Ascorbate Restrains Epithelial–Mesenchymal Transition and Invasion in Glioblastoma Cells via Extracellular H2O2 Generation
Source: Int J Mol Sci. 2026 May 30;27(11):4964. doi: 10.3390/ijms27114964 (PMC13257296; doi:10.3390/ijms27114964)
Supplement: Supplementary file 1 [file ijms-27-04964-s001.zip › 260220 Supplementary table.pdf]

Pharmacological Ascorbate Restrains Epithelial-Mesenchymal-Transition and Invasion in Glioblastoma Cells *via* Extracellular H_2O_2 Generation

Onsurang Wattanathamsan [1], **Naphat Chantaravisoot** [2,3,4], **Rungnapa Bootsri** [2,4], **Nuttiya Kalpongnukul** [4], **Napatsakon Youngsanbhu** [2,4], **Claudia R. Oliva** [5], **Corinne E. Griguer** [5], **Visarut Buranasudja** [1,6,*]

[1] Department of Pharmacology and Physiology, Faculty of Pharmaceutical Sciences, Chulalongkorn University, Bangkok, Thailand

[2] Department of Biochemistry, Faculty of Medicine, Chulalongkorn University, Bangkok 10330, Thailand.

[3] Center of Excellence in Systems Microbiology, Faculty of Medicine, Chulalongkorn University, Bangkok 10330, Thailand.

[4] Center of Excellence in Systems Biology, Faculty of Medicine, Chulalongkorn University, Bangkok 10330, Thailand.

[5] Free Radical & Radiation Biology Program, Department of Radiation Oncology, University of Iowa, Iowa, IA 52242, USA.

[6] Center of Excellence in Natural Products for Ageing and Chronic Diseases, Faculty of Pharmaceutical Sciences, Chulalongkorn University, Bangkok 10330, Thailand.

*** Correspondence:** Visarut Buranasudja, Department of Pharmacology and Physiology, Faculty of Pharmaceutical Sciences, Chulalongkorn University, Bangkok 10330, Thailand

Email: visarut.b@pharm.chula.ac.th

Supplementary Table S1. Detailed information on the chemicals and reagents used in this study

Manufacturer	Chemicals	Catalogue number
ATCC (Manassas, VA, USA)	Universal Mycoplasma Detection Kit	30-1012K
Bioline (London, UK)	SensiFAST™ SYBR® NO-ROX Kit	BIO-98005
Calbiochem (Darmstadt, Germany)	FluorSave™ Reagent	345789
Cell Signaling Technology (Danvers, MA, USA)	4′,6′-diamidino-2-phenylindole (DAPI)	D1306
	Protease/phosphatase inhibitor cocktail (100×)	5872
Corning (NY, USA)	Matrigel® Basement Membrane Matrix	356234
Geneaid Biotech (Taipei, Taiwan)	GENEzol Reagent	GZR100
Invitrogen (Carlsbad, CA, USA)	SuperScript™ III Reverse Transcriptase	170-8841
MilliporeSigma (Burlington, MA, USA)	L-ascorbic acid	A92902
	Bovine serum albumin	12659
	Catalase from bovine liver	C9322
	Crystal violet	101408
	Dimethyl sulfoxide (DMSO)	102952
	FluorSave™ Reagent	345789
	Immobilon Western chemiluminescent HRP substrate	WBKLS0100
	Methanol (100%)	106009
	Methylthiazolyldiphenyl-tetrazolium bromide (MTT)	M5655
	Paraformaldehyde	818715
	Skim milk powder	115363
Stemcell Technologies (Vancouver, BC, Canada)	AZD8055	73004
Thermo Fisher Scientific (Waltham, MA, USA)	Alexa Fluor™ 488 goat anti-rabbit IgG (H&L) secondary antibody	A-31566
	4′,6′-diamidino-2-phenylindole (DAPI)	D1306
	DMEM	12100046
	DMEM/F12	12400024
	Fetal bovine serum	A5256701
	Penicillin-Streptomycin (10,000 U/mL)	15140122
	Pierce BCA protein assay kit	23225
	Radioimmunoprecipitation assay (RIPA) buffer	9806
	Rhodamine phalloidin	R415
	Trypsin	25200-072
Visual Protein Biotechnology (Taipei, Taiwan)	BlockPro™ Protein-Free Blocking Buffer	BF01-1L

Supplementary Table S2. List of primers and their forward and reverse primer sequences used in RT-PCR analysis. All primers were synthesized from GENEWIZ (South Plainfield, NJ, USA).

Genes	Forward (5′→3′)	Reverse (3′→5′)
Claudin-1	TTTACTCCTATGCCGGCGAC	GAGGATGCCAACCACCATCA
N-Cadherin	GACCGAGAATCACCAAATGTG	GCGTTCCTGTTCCACTCATAG
Slug	AGCATTTCAACGCCTCCA	GGATCTCTGGTTGTGGTATGAC
Snail	CTAGCGAGTGGTTCTTCTGC	GTAGTTAGGCTTCCGATTGGG
TWIST1	GGGCCGGAGACCTAGATG	TTTCCAAGAAAATCTTTGGCATA
MMP2	GAAGTATGGGAACGCCGATGG	TTGTCGCGGTCGTAGTCCTCA
MMP9	ATGTACCCTATGTACCGCTTCACT	CAGAGAAGAAGAAAAGCTTCTTGG
Vimentin	ACCCTGCAATCTTTCAGACAG	GATTCCACTTTGCGTTCAAGG
ZEB-1	TTCACAGTGGAGAGAAGCCA	GCCTGGTGATGCTGAAAGAG
GAPDH	ACATCGCTCAGACACCATG	TGTAGTTGAGGTCAATGAAGGG

Supplementary Table S3. List of antibodies and their respective concentrations used in western blot analysis and immunofluorescence analysis. All antibodies were purchased from Cell Signaling Technology (Danvers, MA, USA).

**Antibody**	**Source**	**Catalogue number**	**Concentration**
Primary Antibody			
Akt	rabbit	9272	1:1000
Claudin-1	rabbit	13995	1:1000
GAPDH	rabbit	5174	1:1000
N-Cadherin	rabbit	13116	1:1000
p-Akt (Ser473)	rabbit	4060	1:1000
p-S6 (Ser235/236)	rabbit	4858	1:1000
p-p70 S6 Kinase (Thr389)	rabbit	9234	1:1000
p-4EBP1 (Ser65)	rabbit	9451	1:1000
S6	rabbit	2217	1:1000
p70 S6 Kinase	rabbit	9202	1:1000
Slug	rabbit	9585	1:1000
Snail	rabbit	3879	1:1000
TWIST1	rabbit	90445	1:1000
Vimentin	rabbit	5741	1:1000
ZEB-1	rabbit	70512	1:1000
ZO-1	rabbit	13663	1:1000
4EBP1	rabbit	9644	1:1000
Secondary Antibody			
Anti-rabbit IgG, HRP-linked antibody	goat	7074	1:2000
Anti-mouse IgG, HRP-linked antibody	horse	7076	1:2000

Supplementary Tables S4-S6 are provided as a separate excel file.

- **Supplementary Table S4. Transcriptomics analysis of all genes**
- **Supplementary Table S5. Transcriptomics analysis of cancer hallmark genes**
- **Supplementary Table S6. Transcriptomics analysis of EMT-related genes**
