# Supplementary material for: Pharmacological Ascorbate Restrains Epithelial–Mesenchymal Transition and Invasion in Glioblastoma Cells via Extracellular H2O2 Generation
Source: Int J Mol Sci. 2026 May 30;27(11):4964. doi: 10.3390/ijms27114964 (PMC13257296; doi:10.3390/ijms27114964)
Supplement: Supplementary file 1 [file ijms-27-04964-s001.zip › IJMS_Supplementary Figures.pdf]

Pharmacological Ascorbate Restrains Epithelial-Mesenchymal-Transition and Invasion in Glioblastoma Cells *via* Extracellular H_2O_2 Generation

Onsurang Wattanathamsan [1], Naphat Chantaravisoot [2,3,4], Rungnapa Bootsri [2,4], Nuttiya Kalpongnukul [4], Napatsakon Youngsanbhu [2,4], Claudia R. Oliva [5], Corinne E. Griguer [5], Visarut Buranasudja [1,6,*]

[1] Department of Pharmacology and Physiology, Faculty of Pharmaceutical Sciences, Chulalongkorn University, Bangkok, Thailand

[2] Department of Biochemistry, Faculty of Medicine, Chulalongkorn University, Bangkok 10330, Thailand.

[3] Center of Excellence in Systems Microbiology, Faculty of Medicine, Chulalongkorn University, Bangkok 10330, Thailand.

[4] Center of Excellence in Systems Biology, Faculty of Medicine, Chulalongkorn University, Bangkok 10330, Thailand.

[5] Free Radical & Radiation Biology Program, Department of Radiation Oncology, University of Iowa, Iowa, IA 52242, USA.

[6] Center of Excellence in Natural Products for Ageing and Chronic Diseases, Faculty of Pharmaceutical Sciences, Chulalongkorn University, Bangkok 10330, Thailand.

*** Correspondence:** Visarut Buranasudja, Department of Pharmacology and Physiology, Faculty of Pharmaceutical Sciences, Chulalongkorn University, Bangkok 10330, Thailand

Email: visarut.b@pharm.chula.ac.th

1. Supplementary material and method

1.1 Clonogenic assay

Cells were plated onto a 6-well culture plate at a density of 1.0×10^4 cells per well and incubated for 24 h. Following the specified treatments, cells were washed twice with PBS and collected by trypsinization. The density of cells was determined with a hemocytometer, and the cells were subsequently replated in triplicate in a 6-well plate, with 500 cells per well in 2.0 mL of complete medium. The cells were allowed to grow for 7 days. Afterward, the colonies were fixed with 4% paraformaldehyde for 20 min and stained with 0.1% crystal violet for 15 min. A colony was defined as a cluster containing at least 50 cells.

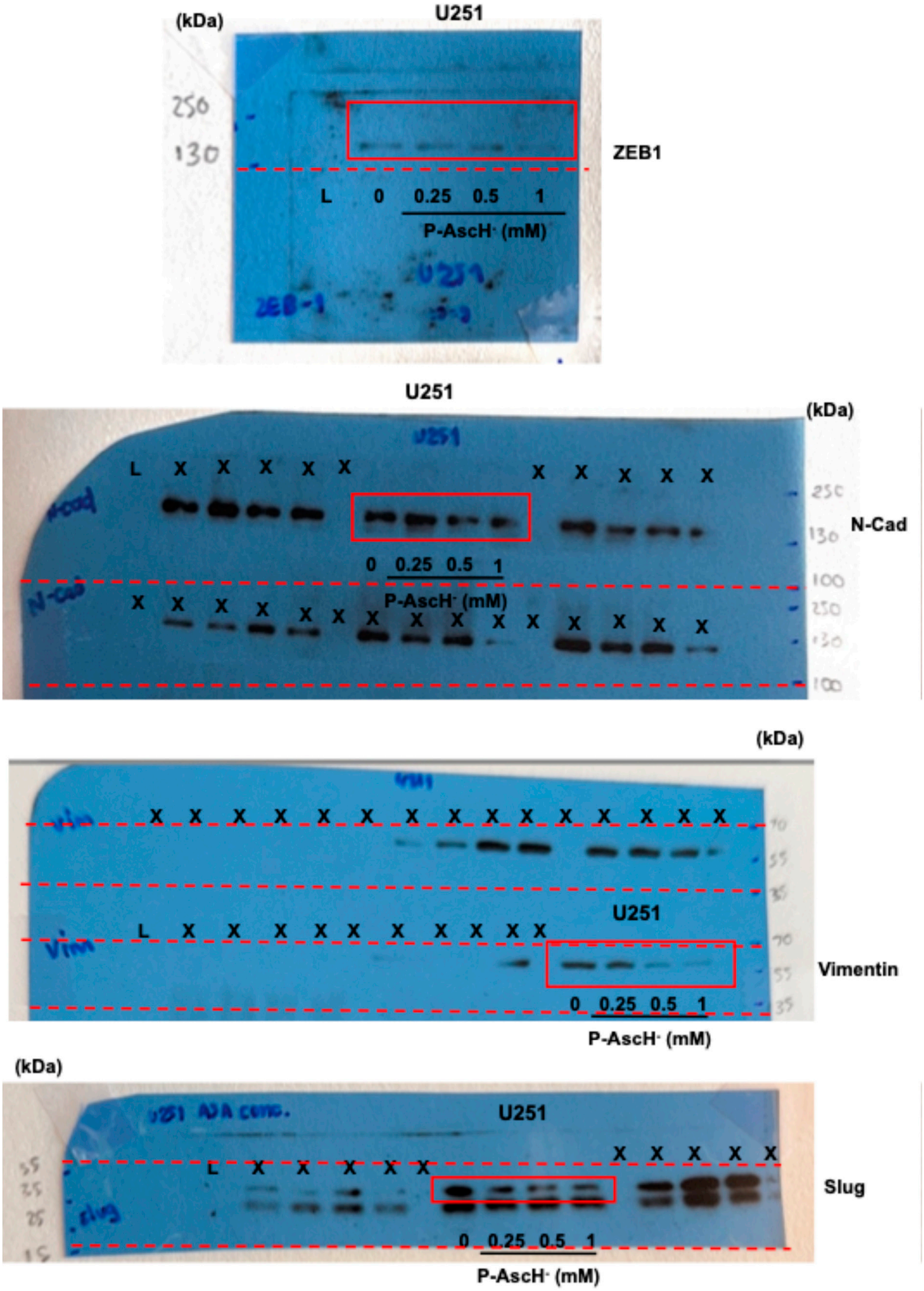
U251
(kDa)
250
130
ZEB1
L 0 0.25 0.5 1
P-AscH- (mM)
U251
(kDa)
N-Cad
0 0.25 0.5 1
P-AscH- (mM)
(kDa)
U251
Vimentin
0 0.25 0.5 1
P-AscH- (mM)
(kDa)
U251
Slug
0 0.25 0.5 1
P-AscH- (mM)

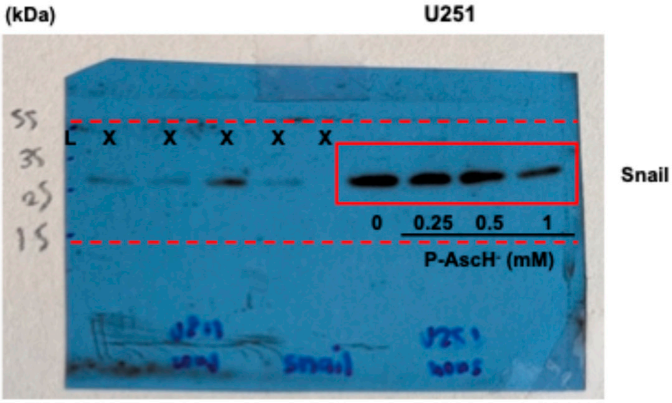
(kDa)
U251
55
35
25
15
L X X X X X
Snail
0 0.25 0.5 1
P-AscH- (mM)

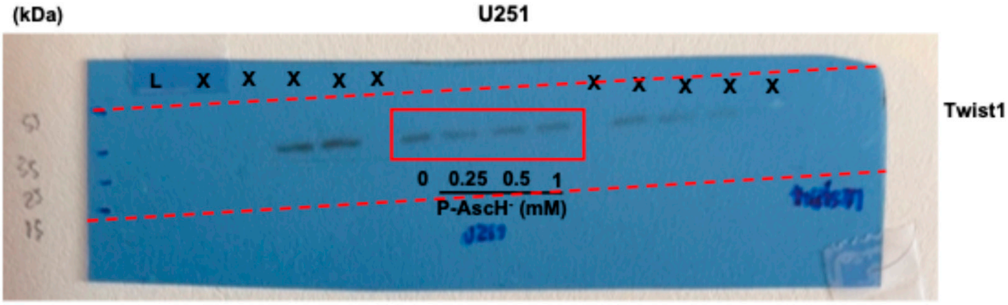
(kDa)
U251
L X X X X X
X X X X X
Twist1
0 0.25 0.5 1
P-AscH- (mM)

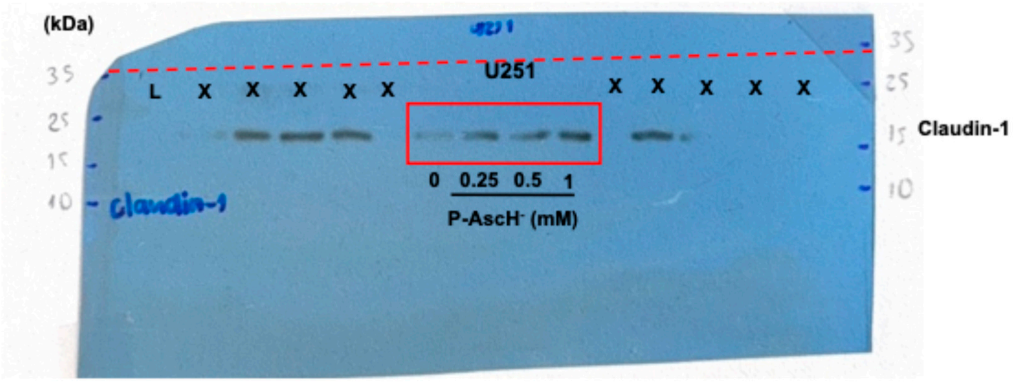
(kDa)
U251
35
25
15
10
L X X X X X
X X X X X
Claudin-1
0 0.25 0.5 1
P-AscH- (mM)

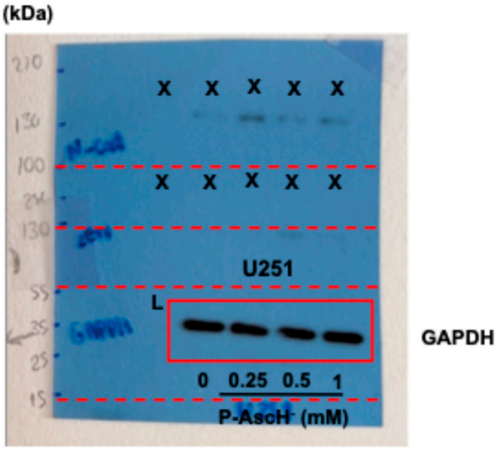
(kDa)
X X X X X
X X X X X
U251
L
GAPDH
0 0.25 0.5 1
P-AscH- (mM)

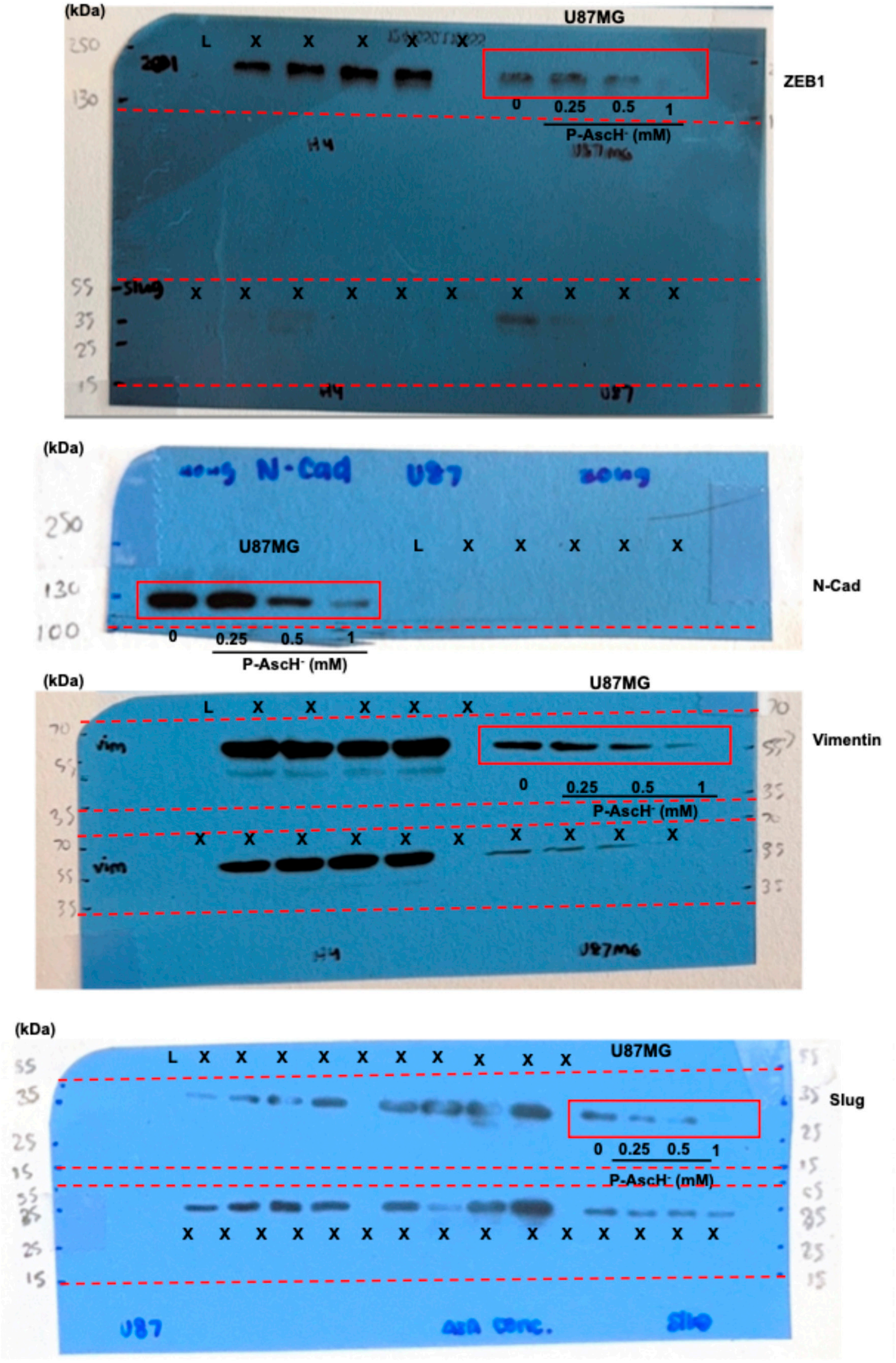
(kDa)
U87MG
ZEB1
0
0.25
0.5
1
P-AscH⁻ (mM)
N-Cad
Vimentin
Slug

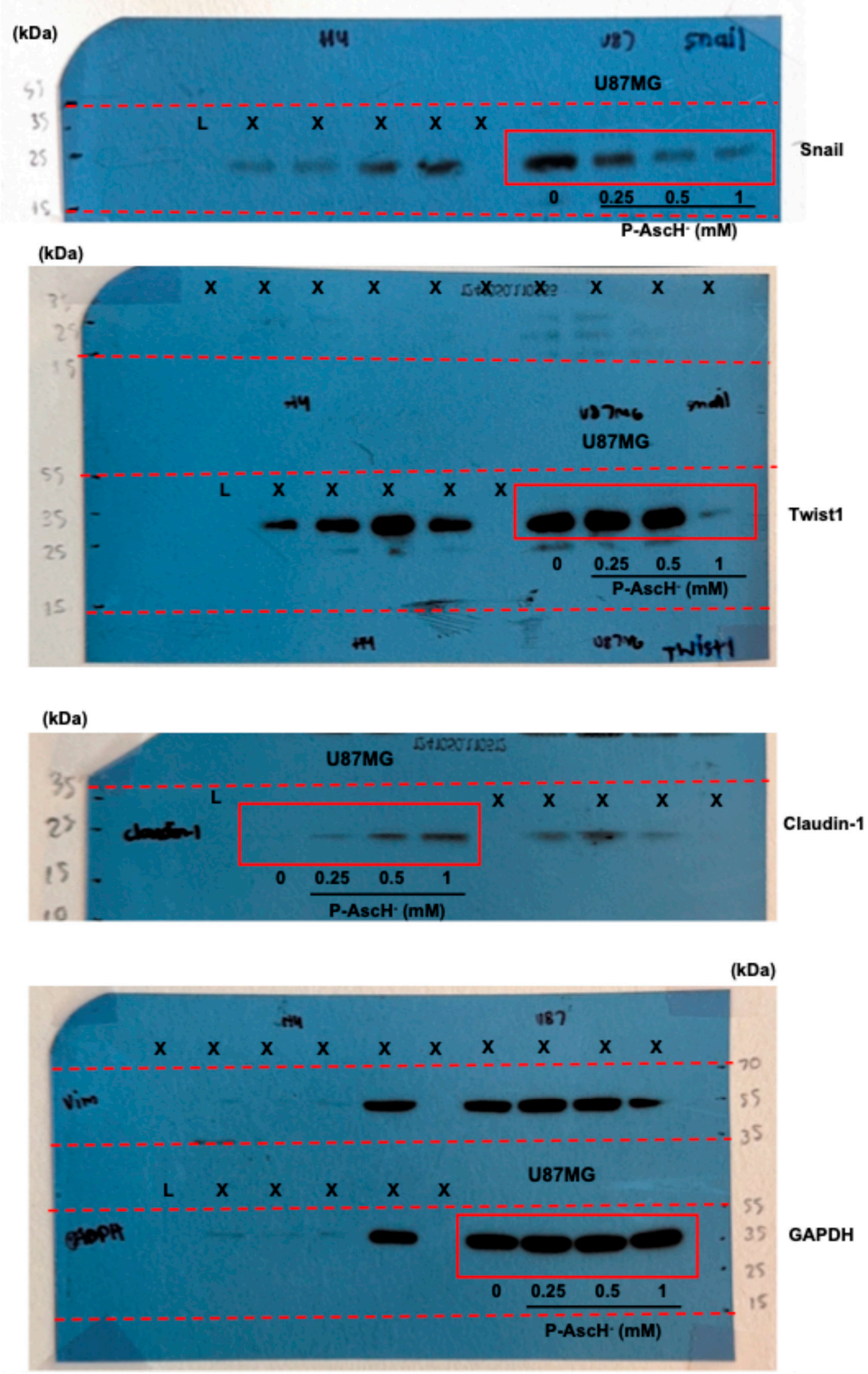

(kDa)
U87MG
L X X X X X
Snail
0 0.25 0.5 1
P-AscH⁻ (mM)
(kDa)
X X X X X X X X X X
U87MG
L X X X X X
Twist1
0 0.25 0.5 1
P-AscH⁻ (mM)
(kDa)
U87MG
L X X X X X
Claudin-1
0 0.25 0.5 1
P-AscH⁻ (mM)
(kDa)
X X X X X X X X X X
U87MG
L X X X X X
GAPDH
0 0.25 0.5 1
P-AscH⁻ (mM)

Supplementary Figure S1. Unedited original western blot images corresponding to the data presented in Figure 8A

The detected proteins and their expected molecular weights are as follows: ZEB1 (200 kDa), N-Cadherin (140 kDa), Vimentin (57 kDa), GAPDH (37 kDa), Slug (30 kDa), Snail (29 kDa), Twist1 (26 kDa), and Claudin-1 (20 kDa). Molecular weight markers were annotated according to the protein ladder reference. Dashed lines indicate regions where membranes were sectioned prior to incubation with the respective primary antibodies. Red boxes indicate the lanes included in the final assembled figure corresponding to the labeled proteins. “L” denotes the molecular weight ladder lane, whereas “X” indicates lanes that were excluded from the final figure.

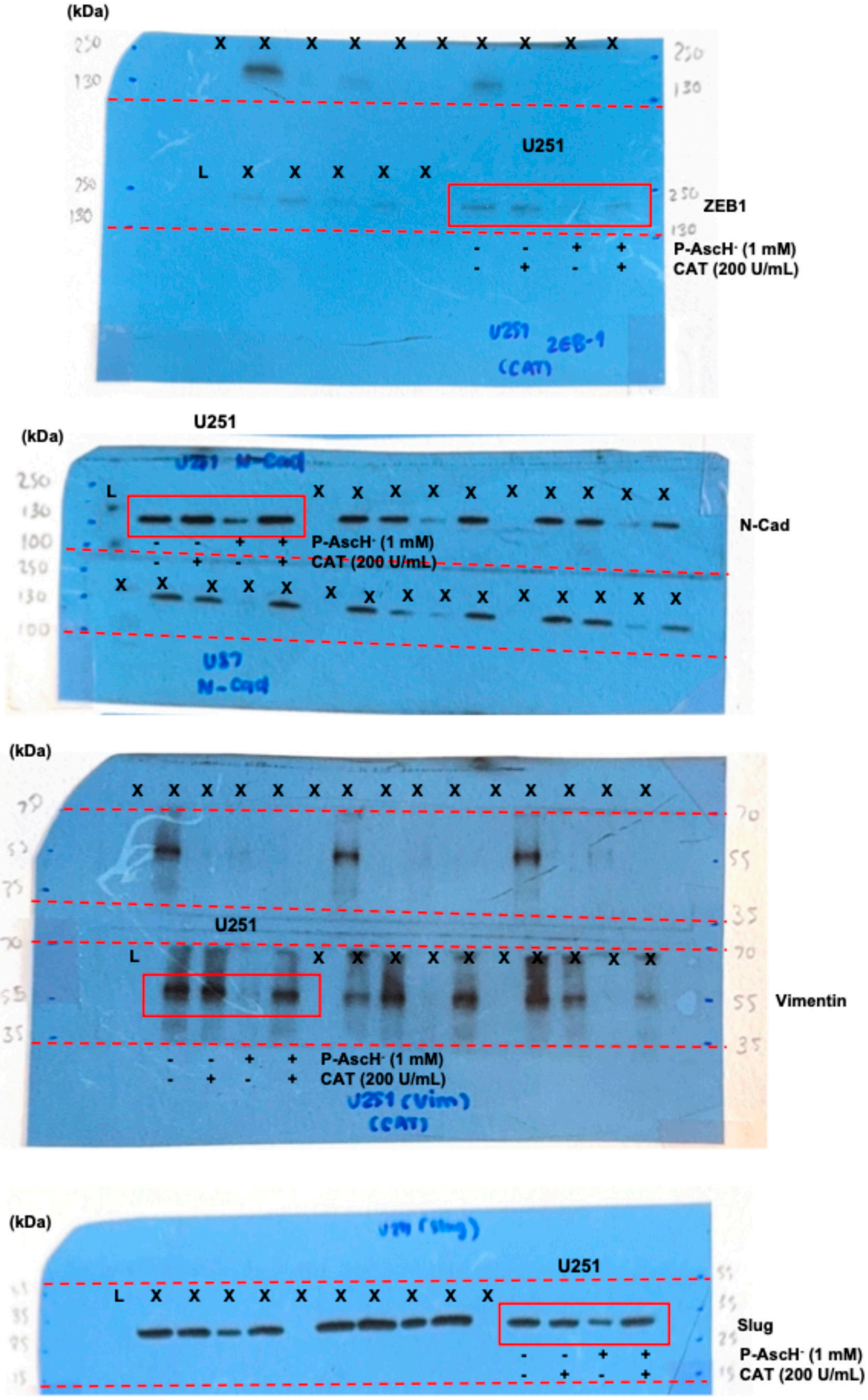

(kDa)
U251
ZEB1
P-AscH- (1 mM)
CAT (200 U/mL)
(kDa)
U251
N-Cad
P-AscH- (1 mM)
CAT (200 U/mL)
(kDa)
U251
Vimentin
P-AscH- (1 mM)
CAT (200 U/mL)
(kDa)
U251
Slug
P-AscH- (1 mM)
CAT (200 U/mL)

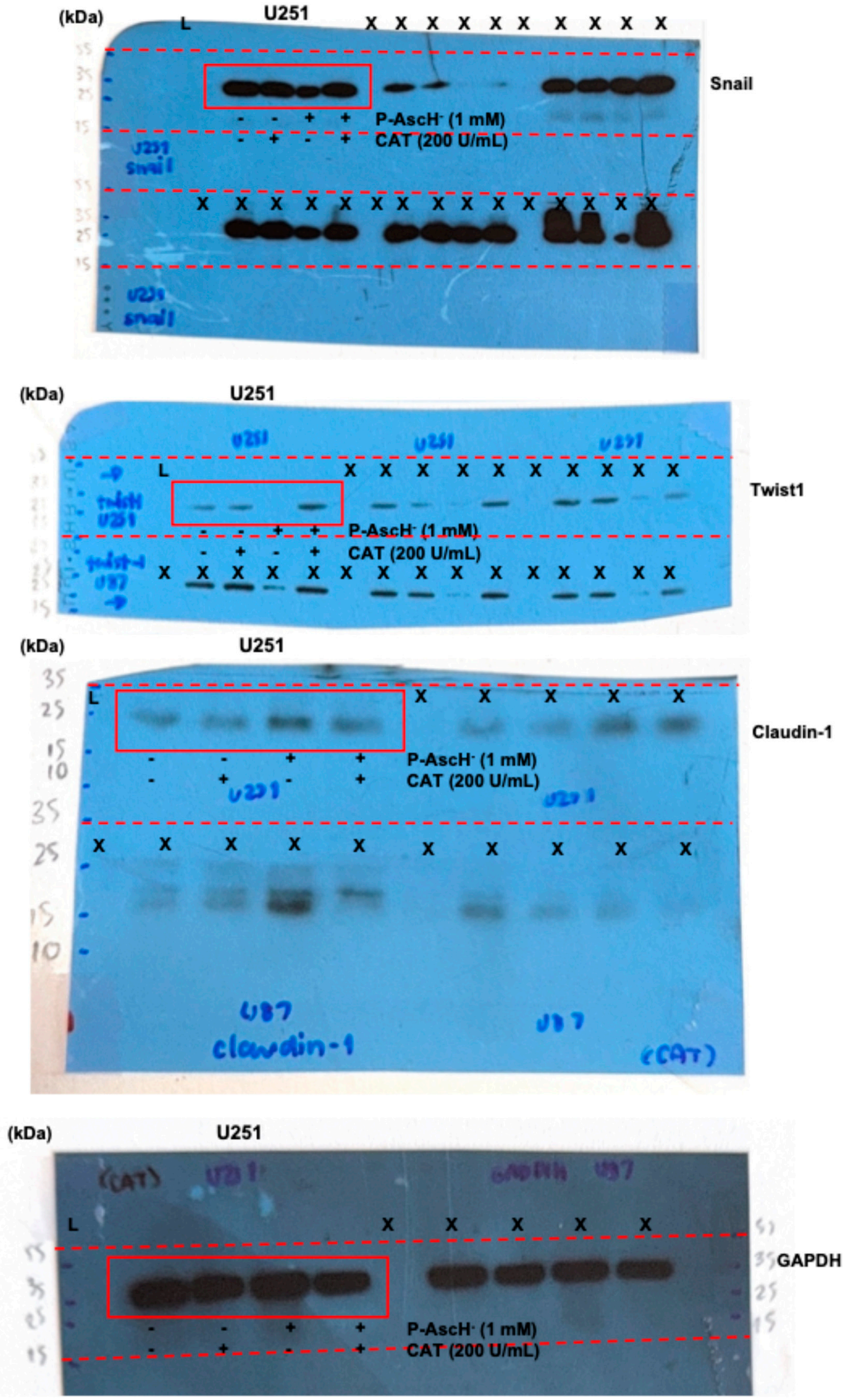
(kDa)
U251
L
Snail
P-AscH- (1 mM)
CAT (200 U/mL)
(kDa)
U251
L
Twist1
P-AscH- (1 mM)
CAT (200 U/mL)
(kDa)
U251
L
Claudin-1
P-AscH- (1 mM)
CAT (200 U/mL)
(kDa)
U251
L
GAPDH
P-AscH- (1 mM)
CAT (200 U/mL)

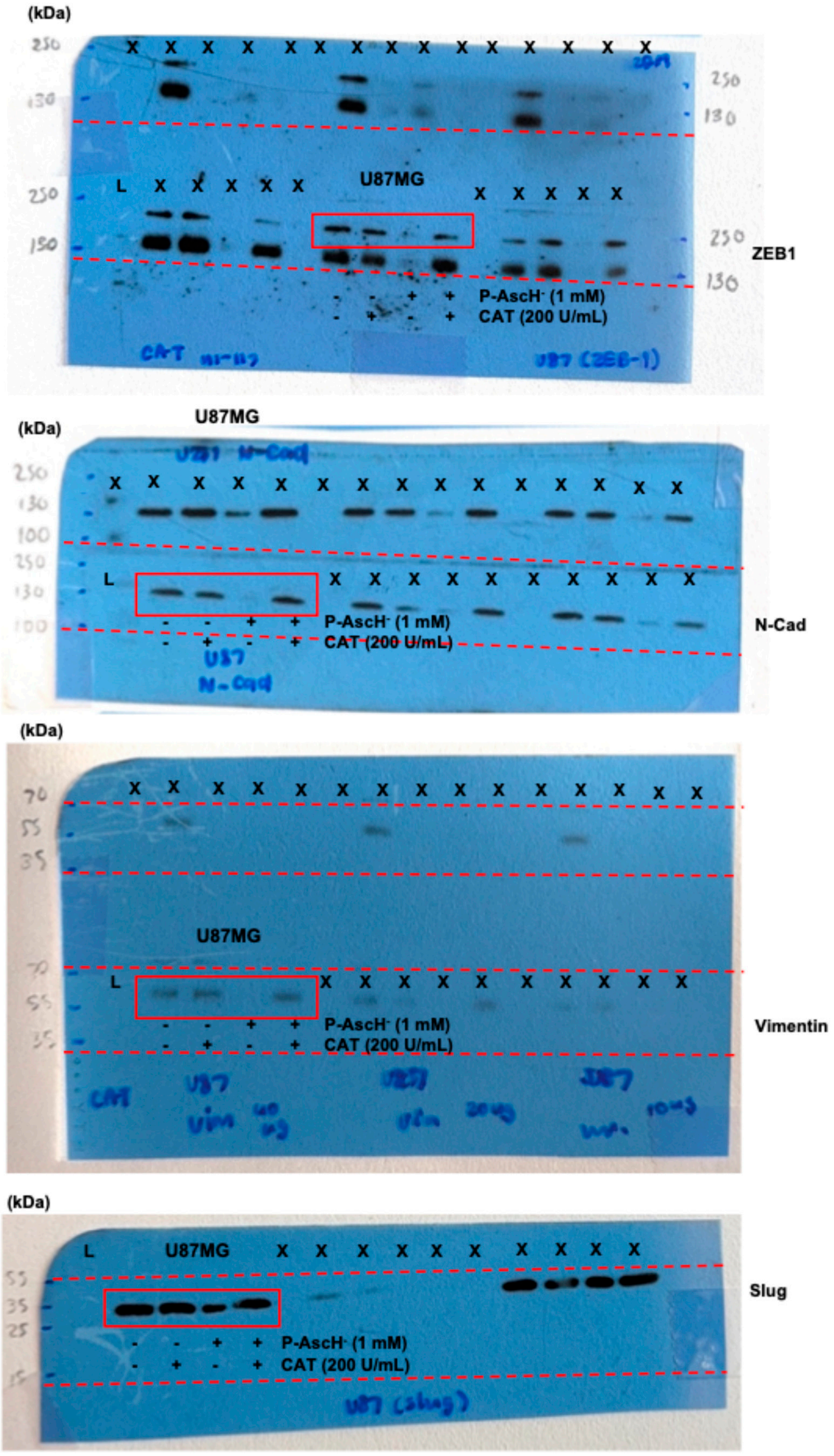
(kDa)
U87MG
ZEB1
P-AscH⁻ (1 mM)
CAT (200 U/mL)
(kDa)
U87MG
N-Cad
P-AscH⁻ (1 mM)
CAT (200 U/mL)
(kDa)
U87MG
Vimentin
P-AscH⁻ (1 mM)
CAT (200 U/mL)
(kDa)
U87MG
Slug
P-AscH⁻ (1 mM)
CAT (200 U/mL)

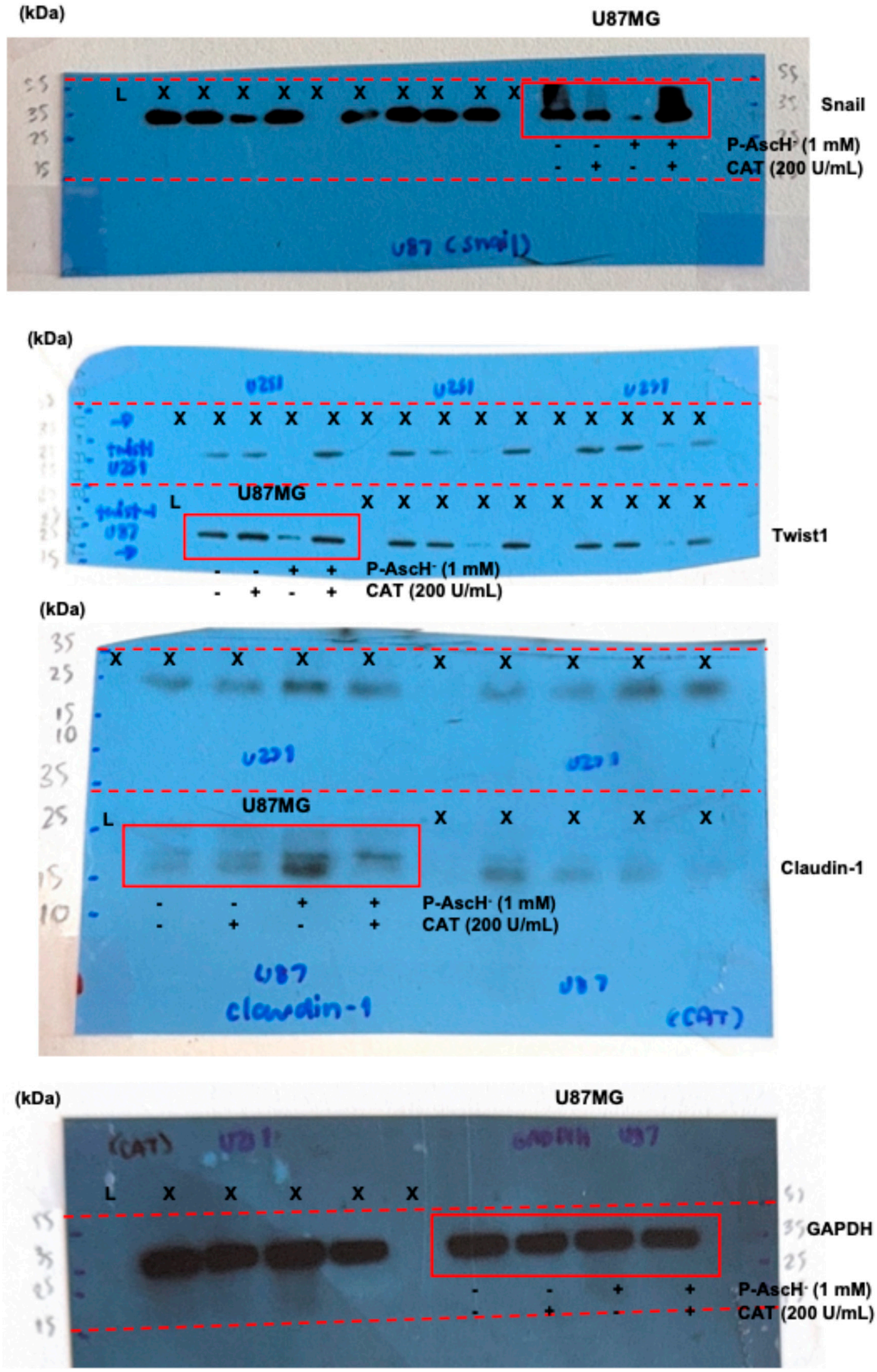

(kDa)
U87MG
Snail
P-AscH- (1 mM)
CAT (200 U/mL)
(kDa)
U87MG
Twist1
P-AscH- (1 mM)
CAT (200 U/mL)
(kDa)
U87MG
Claudin-1
P-AscH- (1 mM)
CAT (200 U/mL)
(kDa)
U87MG
GAPDH
P-AscH- (1 mM)
CAT (200 U/mL)

Supplementary Figure S2. Unedited original western blot images corresponding to the data presented in Figure 8C

The detected proteins and their expected molecular weights are as follows: ZEB1 (200 kDa), N-Cadherin (140 kDa), Vimentin (57 kDa), GAPDH (37 kDa), Slug (30 kDa), Snail (29 kDa), Twist1 (26 kDa), and Claudin-1 (20 kDa). Molecular weight markers were annotated according to the protein ladder reference. Dashed lines indicate regions where membranes were sectioned prior to incubation with the respective primary antibodies. Red boxes indicate the lanes included in the final assembled figure corresponding to the labeled proteins. “L” denotes the molecular weight ladder lane, whereas “X” indicates lanes that were excluded from the final figure.

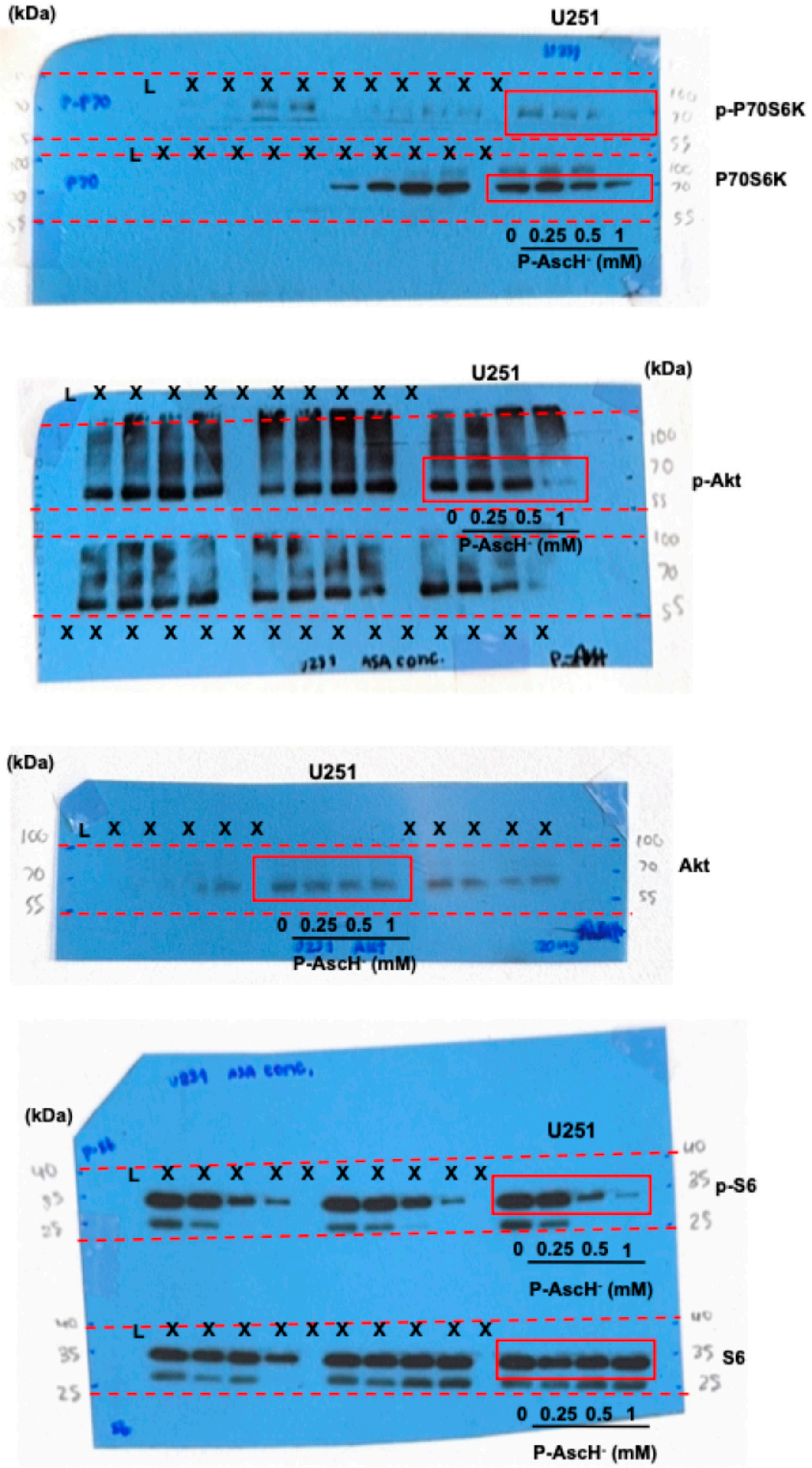
(kDa)
U251
p-P70S6K
P70S6K
0 0.25 0.5 1
P-AscH- (mM)
U251
(kDa)
p-Akt
0 0.25 0.5 1
P-AscH- (mM)
(kDa)
U251
Akt
0 0.25 0.5 1
P-AscH- (mM)
(kDa)
U251
p-S6
0 0.25 0.5 1
P-AscH- (mM)
S6
0 0.25 0.5 1
P-AscH- (mM)

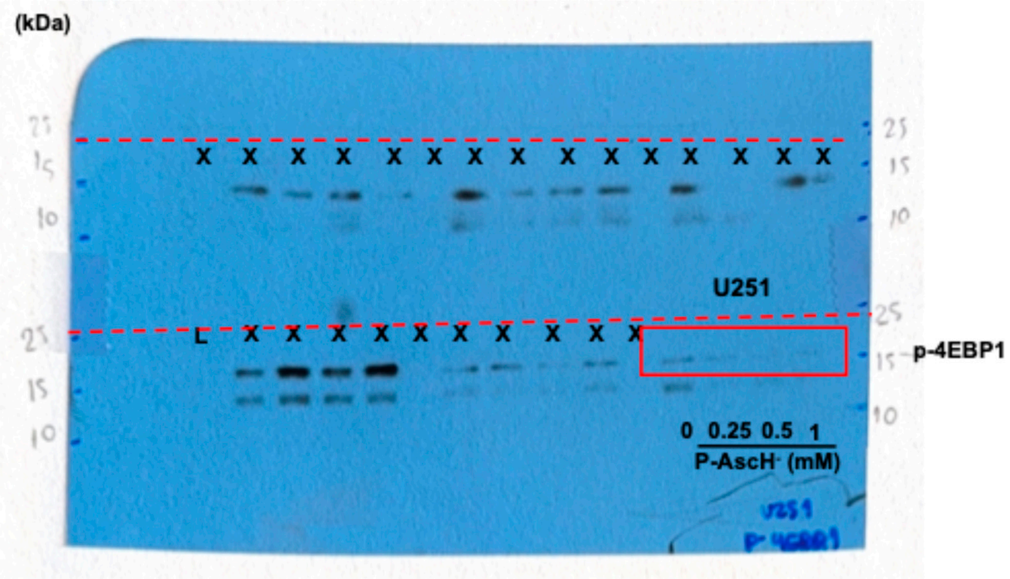
(kDa)
25
15
10
X X X X X X X X X X X X X X X
U251
L X X X X X X X X X X
p-4EBP1
0 0.25 0.5 1
P-AscH- (mM)
U251
P-4EBP1

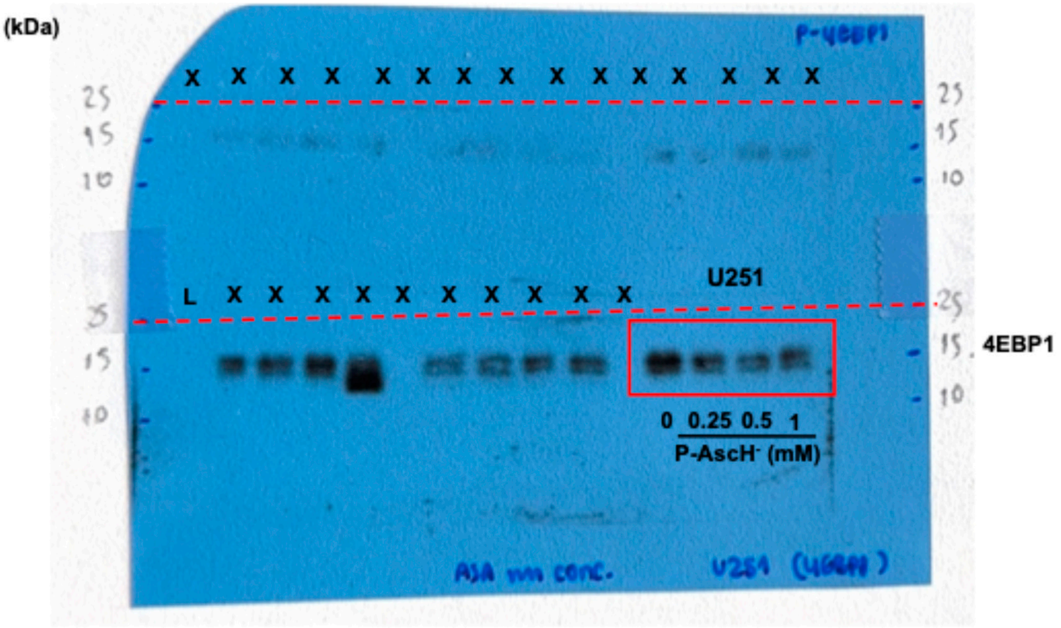
(kDa)
P-4EBP1
X X X X X X X X X X X X X X X
25
15
10
U251
L X X X X X X X X X X
4EBP1
0 0.25 0.5 1
P-AscH- (mM)
ASA mm conc.
U251 (4EBP1)

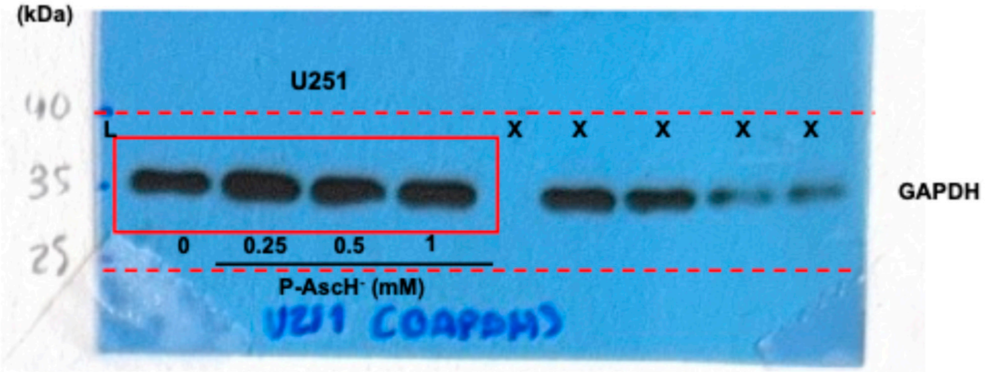
(kDa)
U251
40
35
25
L
X X X X X
GAPDH
0 0.25 0.5 1
P-AscH- (mM)
U251 (GAPDH)

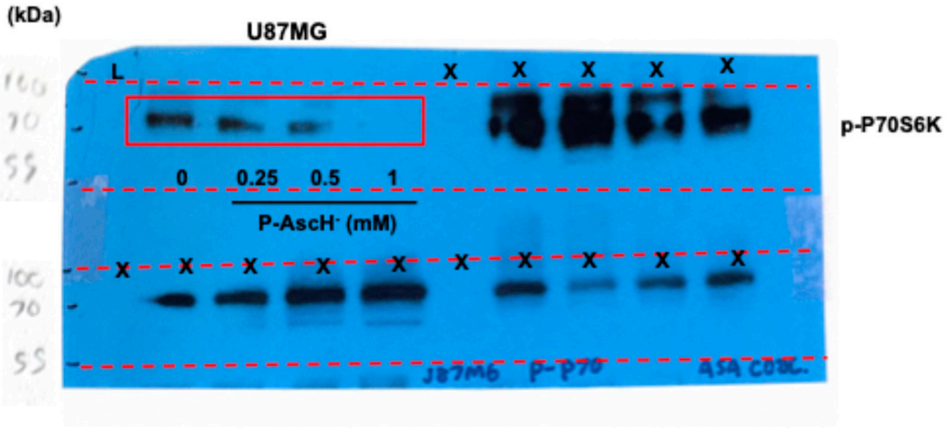
(kDa)
U87MG
p-P70S6K
0
0.25
0.5
1
P-AscH⁻ (mM)

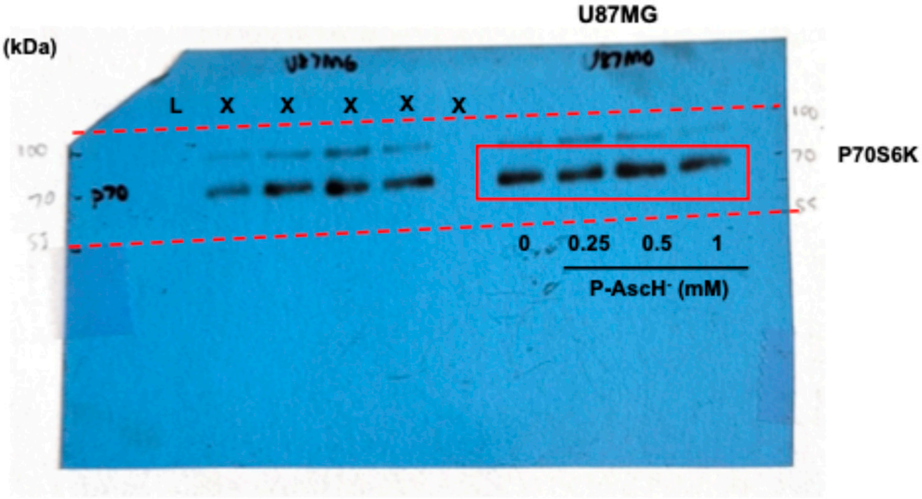
(kDa)
U87MG
P70S6K
0
0.25
0.5
1
P-AscH⁻ (mM)

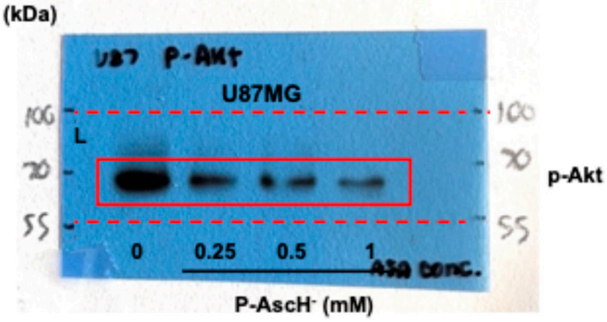
(kDa)
U87MG
p-Akt
0
0.25
0.5
1
P-AscH⁻ (mM)

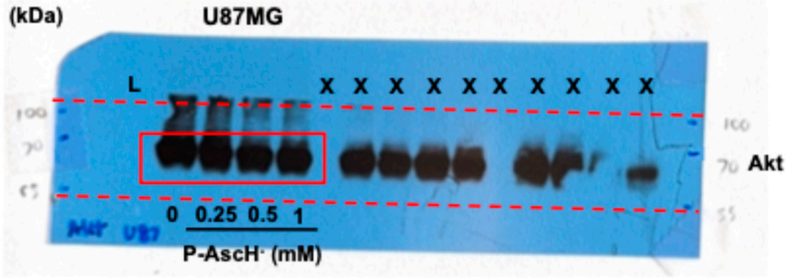
(kDa)
U87MG
Akt
0
0.25
0.5
1
P-AscH⁻ (mM)

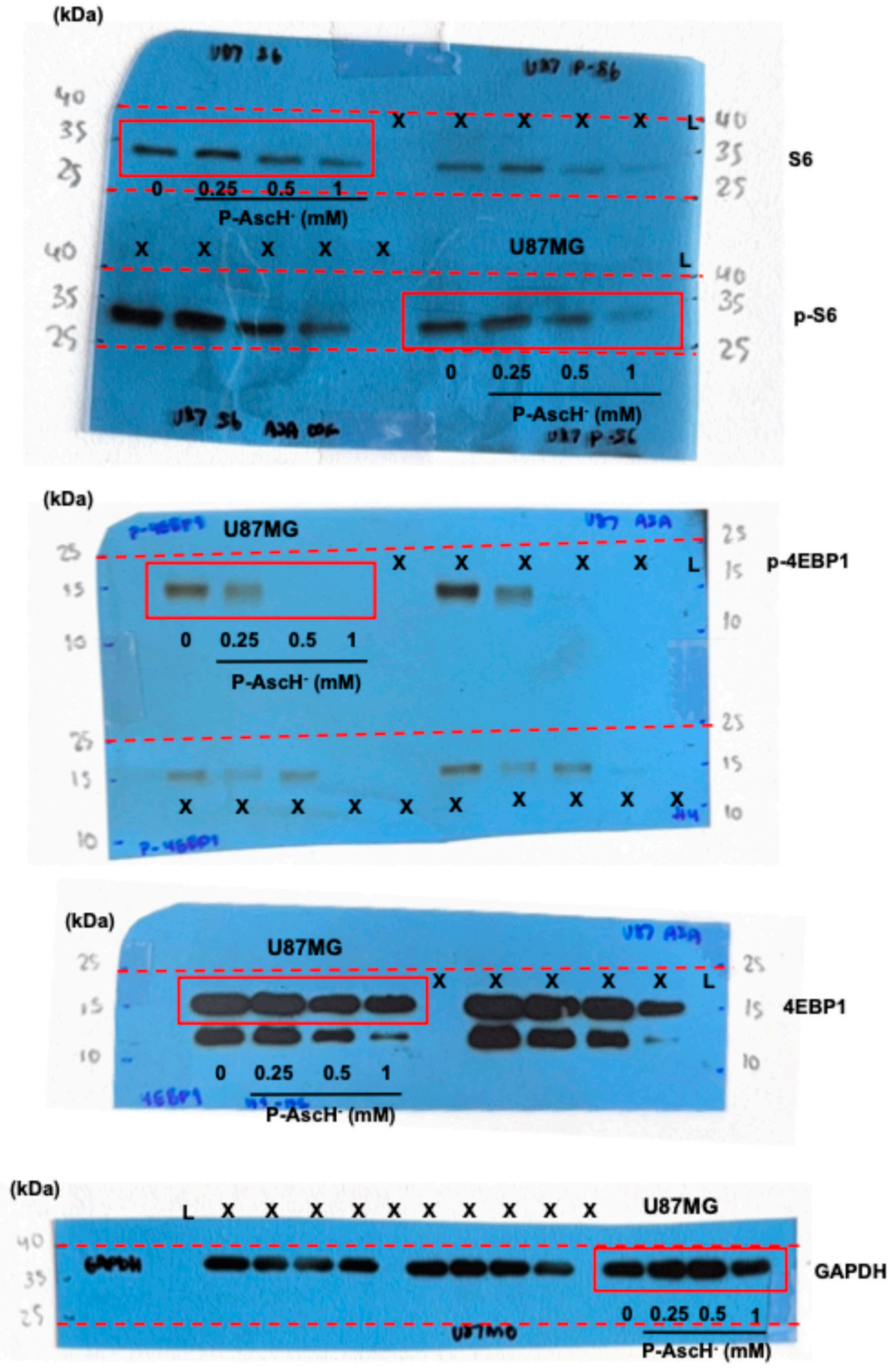
(kDa)
S6
0 0.25 0.5 1
P-AscH- (mM)
U87MG
p-S6
0 0.25 0.5 1
P-AscH- (mM)
(kDa)
U87MG
p-4EBP1
0 0.25 0.5 1
P-AscH- (mM)
(kDa)
U87MG
4EBP1
0 0.25 0.5 1
P-AscH- (mM)
(kDa)
U87MG
GAPDH
0 0.25 0.5 1
P-AscH- (mM)

Supplementary Figure S3. Unedited original western blot images corresponding to the data presented in Figure 9A

The detected proteins and their expected molecular weights are as follows: p-p70S6K (70 kDa), p70S6K (70 kDa), p-Akt (60 kDa), Akt (60 kDa), GAPDH (37 kDa), p-S6 (32 kDa), S6 (32 kDa), p-4EBP1 (15-20 kDa), and 4EBP1 (15-20 kDa). Molecular weight markers were annotated according to the protein ladder reference. Dashed lines indicate regions where membranes were sectioned prior to incubation with the respective primary antibodies. Red boxes indicate the lanes included in the final assembled figure corresponding to the labeled proteins. "L" denotes the molecular weight ladder lane, whereas "X" indicates lanes that were excluded from the final figure.

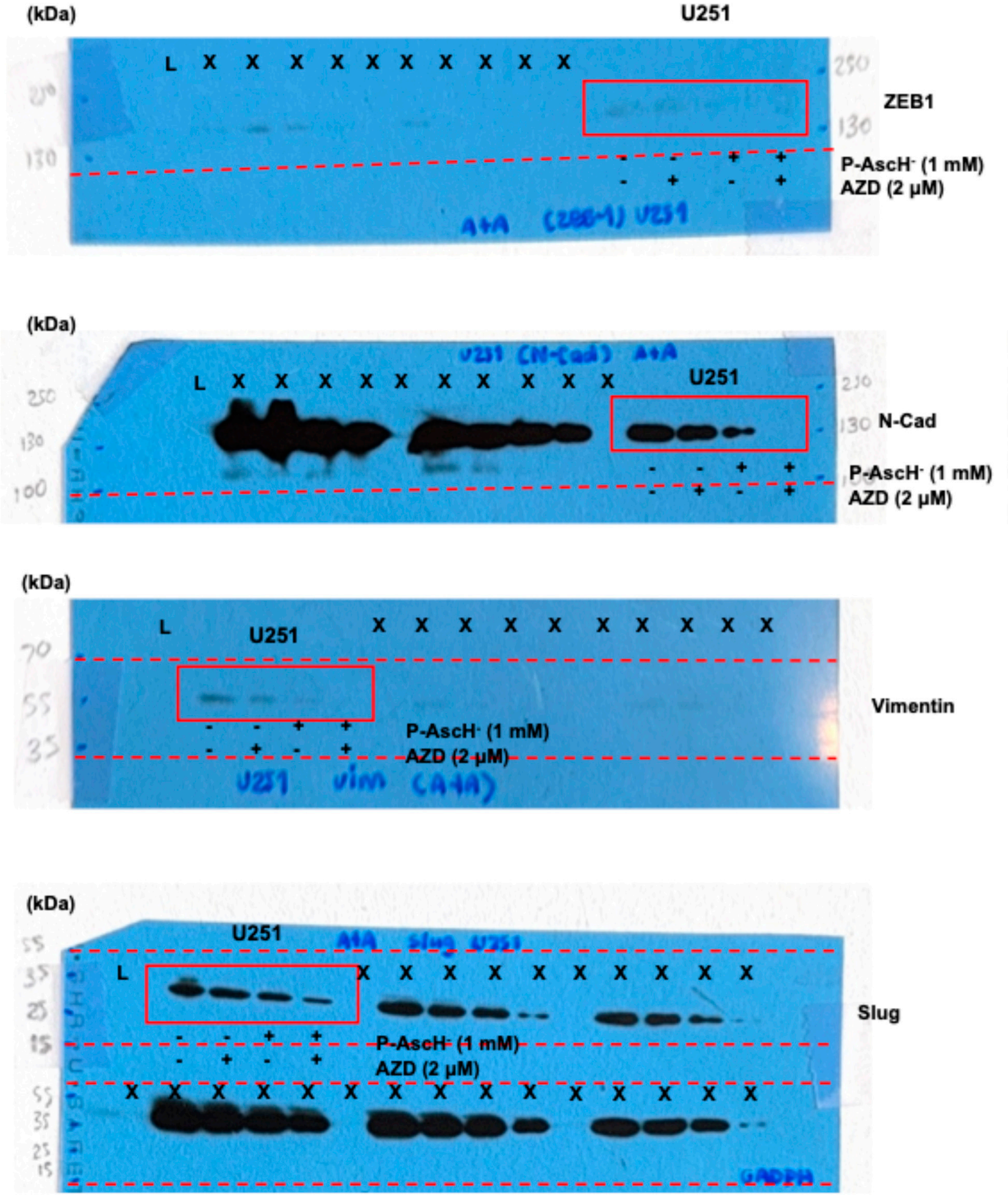
(kDa)
U251
ZEB1
P-AscH⁻ (1 mM)
AZD (2 μM)
(kDa)
U251
N-Cad
P-AscH⁻ (1 mM)
AZD (2 μM)
(kDa)
U251
Vimentin
P-AscH⁻ (1 mM)
AZD (2 μM)
(kDa)
U251
Slug
P-AscH⁻ (1 mM)
AZD (2 μM)

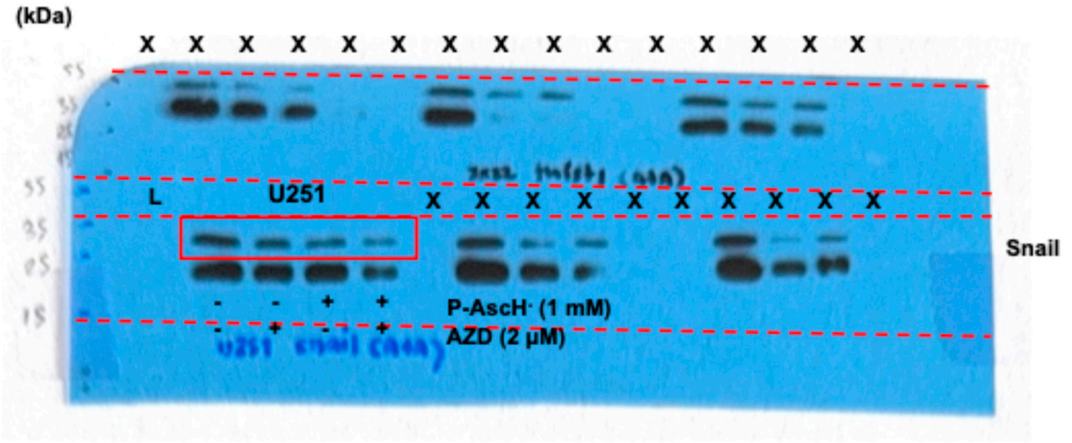
(kDa)
X X X X X X X X X X X X X X X
L
U251
X X X X X X X X X X
Snail
- - + +
- + - +
P-AscH- (1 mM)
AZD (2 µM)

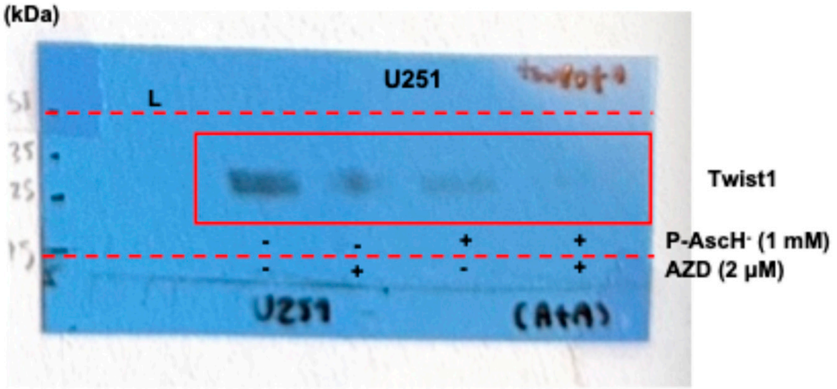
(kDa)
U251
L
Twist1
- - + +
- + - +
P-AscH- (1 mM)
AZD (2 µM)

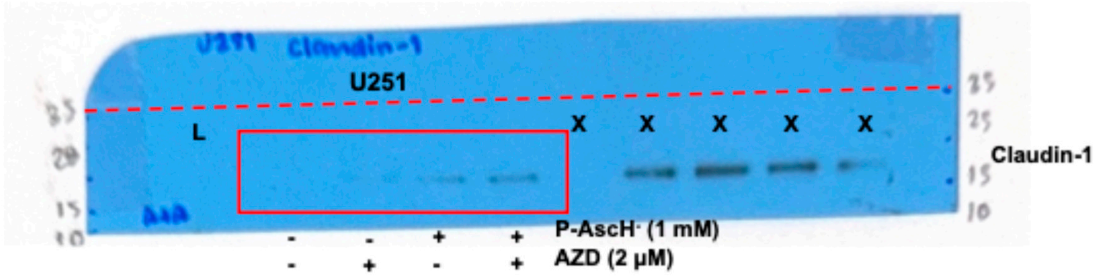
U251
L
X X X X X
Claudin-1
- - + +
- + - +
P-AscH- (1 mM)
AZD (2 µM)

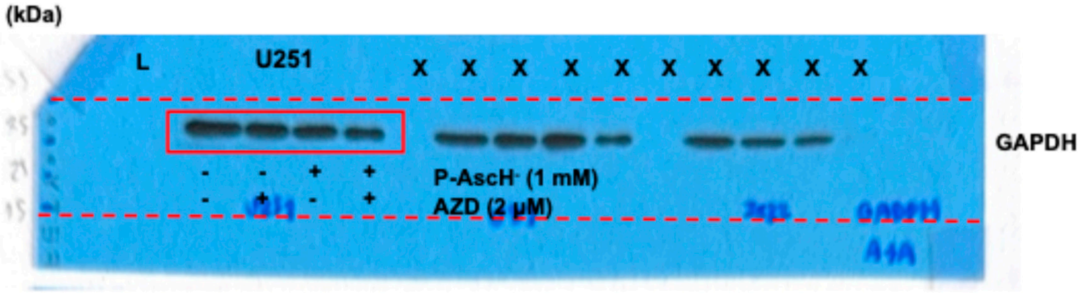
(kDa)
L
U251
X X X X X X X X X X
GAPDH
- - + +
- + - +
P-AscH- (1 mM)
AZD (2 µM)

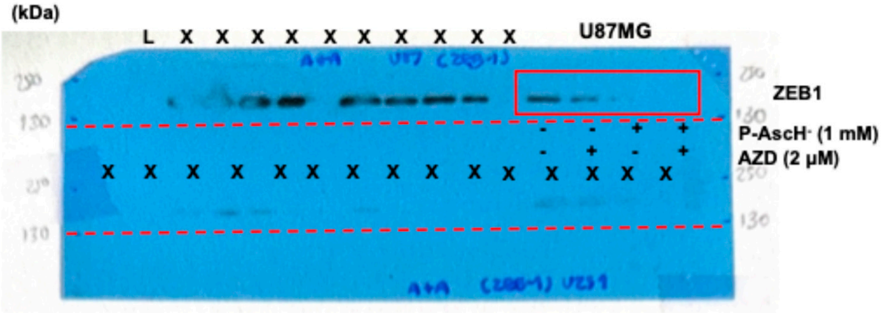
(kDa)
L X X X X X X X X X X
U87MG
ZEB1
P-AscH- (1 mM)
AZD (2 µM)
X X X X X X X X X X X X X X X

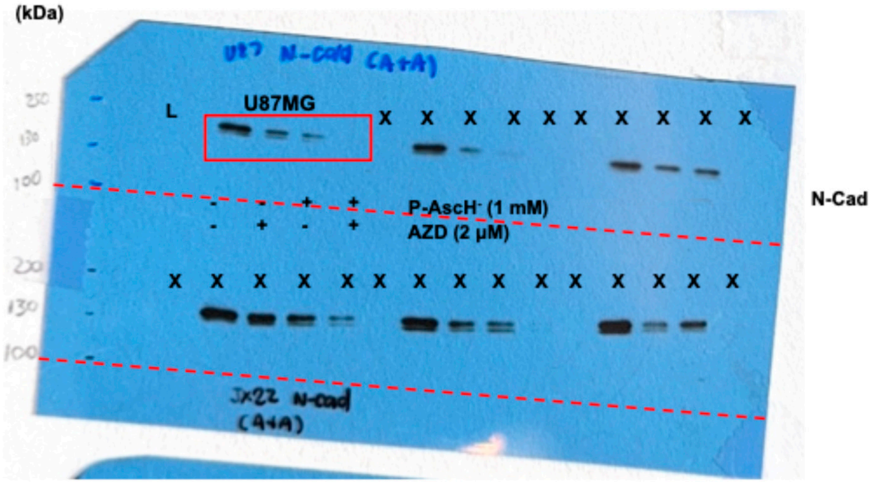
(kDa)
L
U87MG
X X X X X X X X X
N-Cad
P-AscH- (1 mM)
AZD (2 µM)
X X X X X X X X X X X X X X

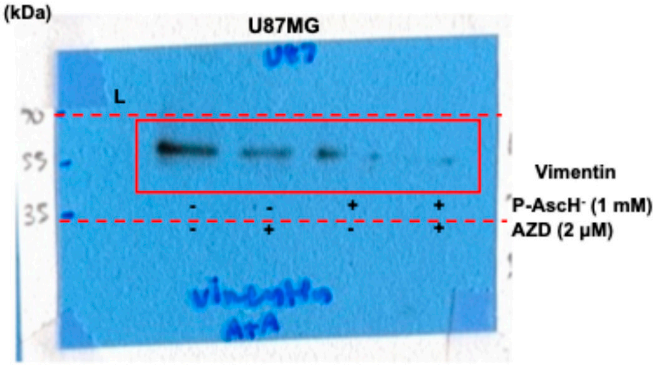
(kDa)
U87MG
L
Vimentin
P-AscH- (1 mM)
AZD (2 µM)

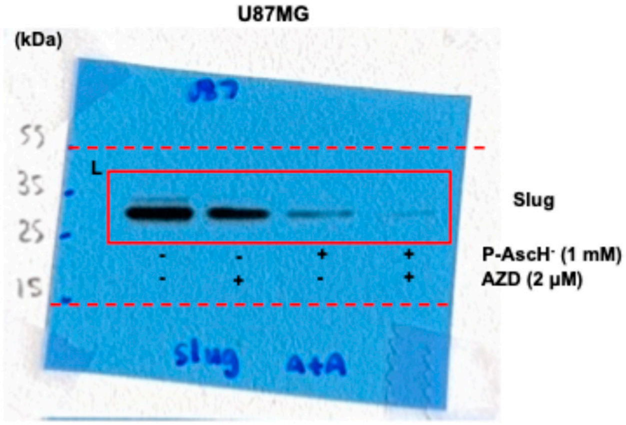
U87MG
(kDa)
L
Slug
P-AscH- (1 mM)
AZD (2 µM)

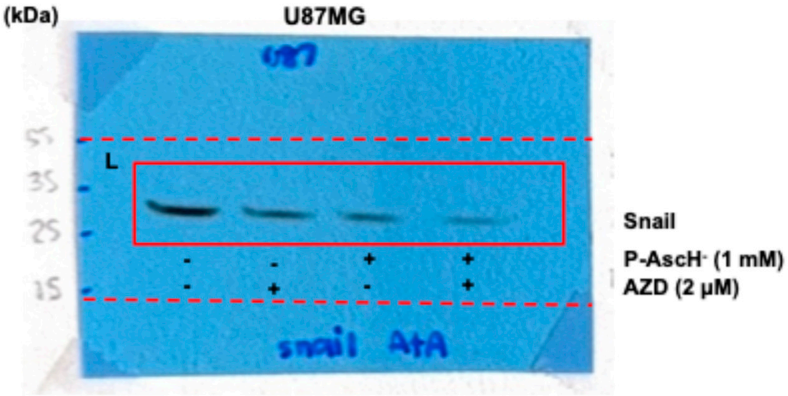
(kDa)
U87MG
L
Snail
P-AscH⁻ (1 mM)
AZD (2 μM)

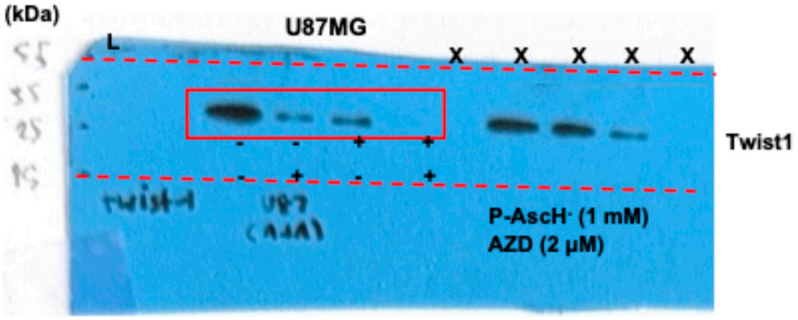
(kDa)
U87MG
L
X X X X X
Twist1
P-AscH⁻ (1 mM)
AZD (2 μM)

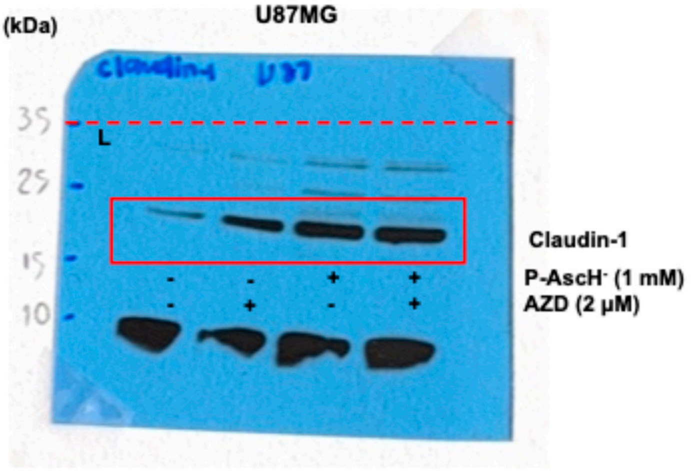
(kDa)
U87MG
L
Claudin-1
P-AscH⁻ (1 mM)
AZD (2 μM)

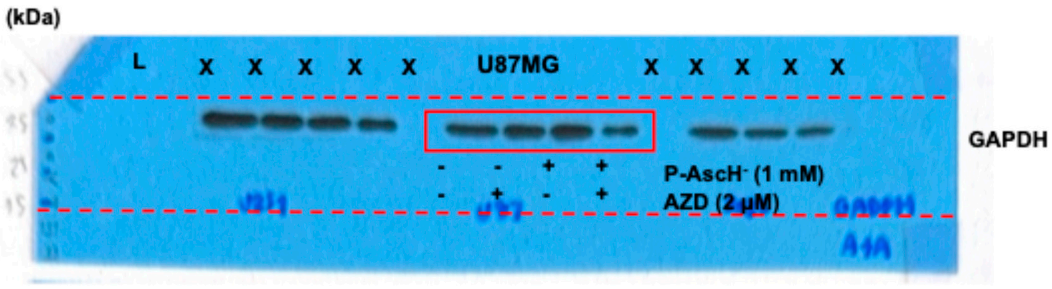
(kDa)
L
X X X X X
U87MG
X X X X X
GAPDH
P-AscH⁻ (1 mM)
AZD (2 μM)

Supplementary Figure S4. Unedited original western blot images corresponding to the data presented in Figure 10E

The detected proteins and their expected molecular weights are as follows: ZEB1 (200 kDa), N-cadherin (140 kDa), Vimentin (57 kDa), GAPDH (37 kDa), Slug (30 kDa), Snail (29 kDa), Twist1 (26 kDa), and Claudin-1 (20 kDa). Molecular weight markers were annotated according to the protein ladder reference. Dashed lines indicate regions where membranes were sectioned prior to incubation with the respective primary antibodies. Red boxes indicate the lanes included in the final assembled figure corresponding to the labeled proteins. "L" denotes the molecular weight ladder lane, whereas "X" indicates lanes that were excluded from the final figure.

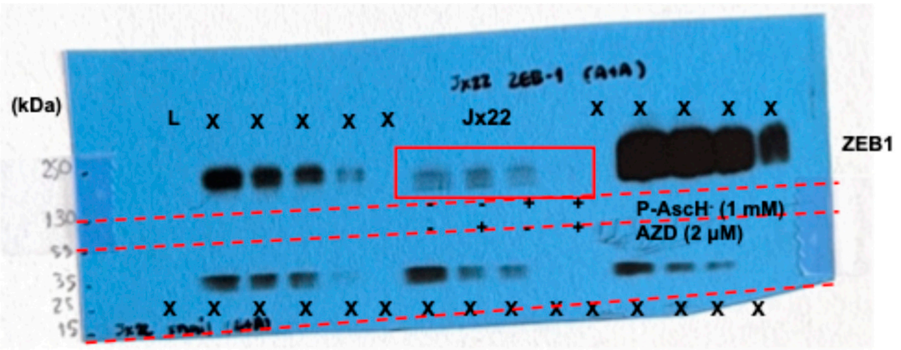
(kDa)
L
Jx22
ZEB1
P-AscH⁻ (1 mM)
AZD (2 µM)

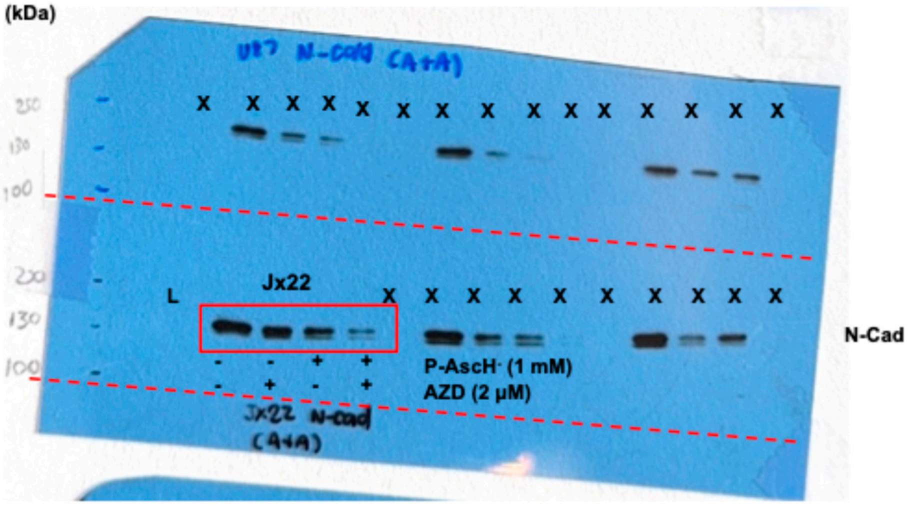
(kDa)
L
Jx22
N-Cad
P-AscH⁻ (1 mM)
AZD (2 µM)

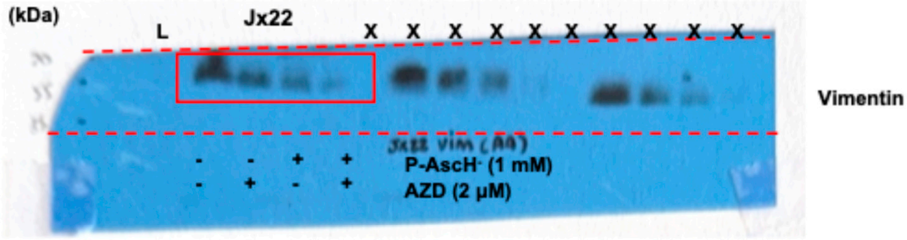
(kDa)
L
Jx22
Vimentin
P-AscH⁻ (1 mM)
AZD (2 µM)

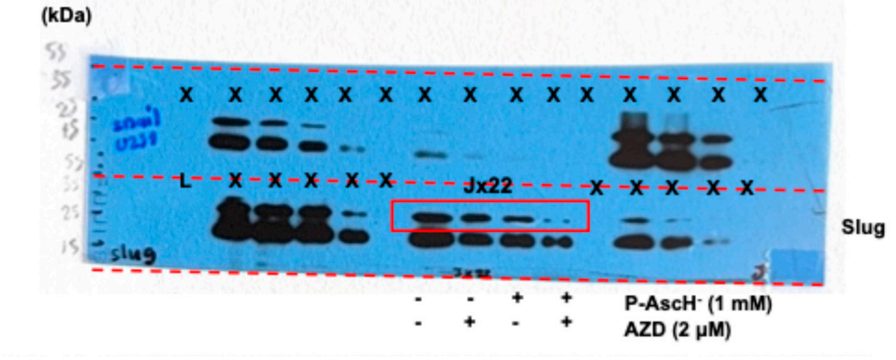
(kDa)
L
Jx22
Slug
P-AscH⁻ (1 mM)
AZD (2 µM)

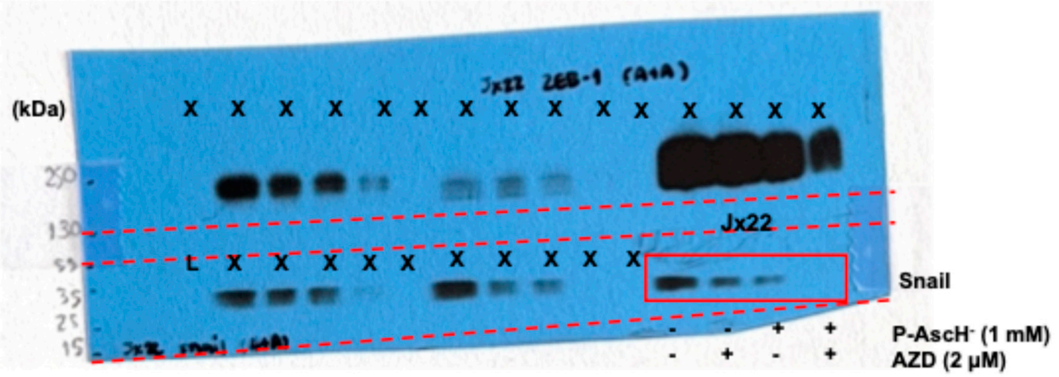
(kDa)
Jx22
Snail
P-AscH- (1 mM)
AZD (2 µM)

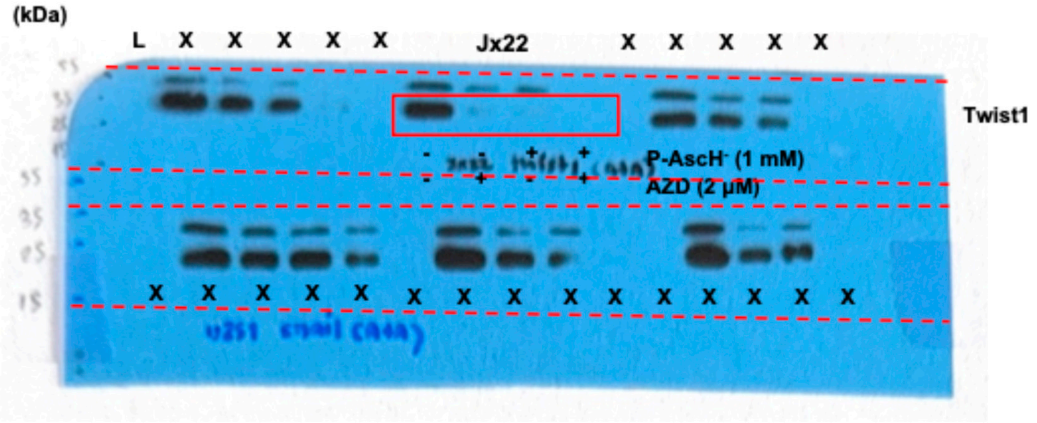
(kDa)
Jx22
Twist1
P-AscH- (1 mM)
AZD (2 µM)

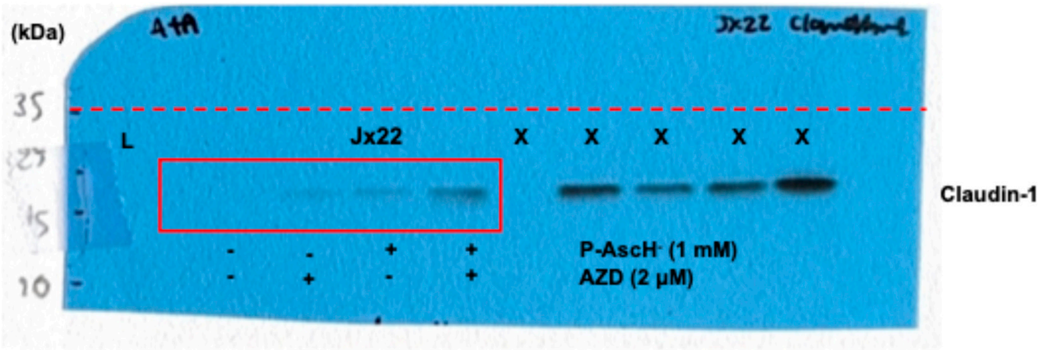
(kDa)
Jx22
Claudin-1
P-AscH- (1 mM)
AZD (2 µM)

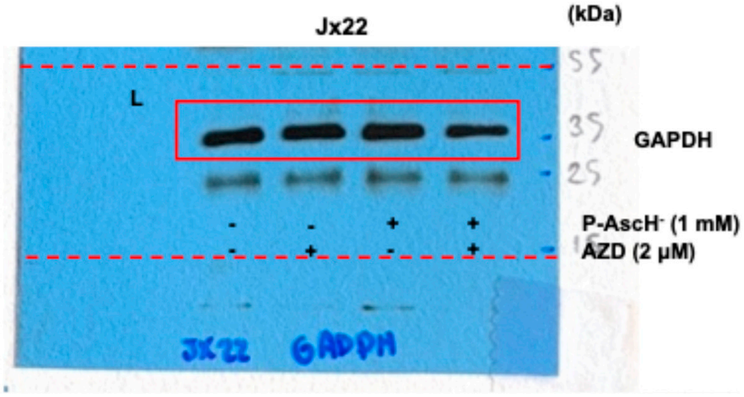
Jx22
(kDa)
GAPDH
P-AscH- (1 mM)
AZD (2 µM)

Supplementary Figure S5. Unedited original western blot images corresponding to the data presented in Figure 11D

The detected proteins and their expected molecular weights are as follows: ZEB1 (200 kDa), N-cadherin (140 kDa), Vimentin (57 kDa), GAPDH (37 kDa), Slug (30 kDa), Snail (29 kDa), Twist1 (26 kDa), and Claudin-1 (20 kDa). Molecular weight markers were annotated according to the protein ladder reference. Dashed lines indicate regions where membranes were sectioned prior to incubation with the respective primary antibodies. Red boxes indicate the lanes included in the final assembled figure corresponding to the labeled proteins. “L” denotes the molecular weight ladder lane, whereas “X” indicates lanes that were excluded from the final figure.

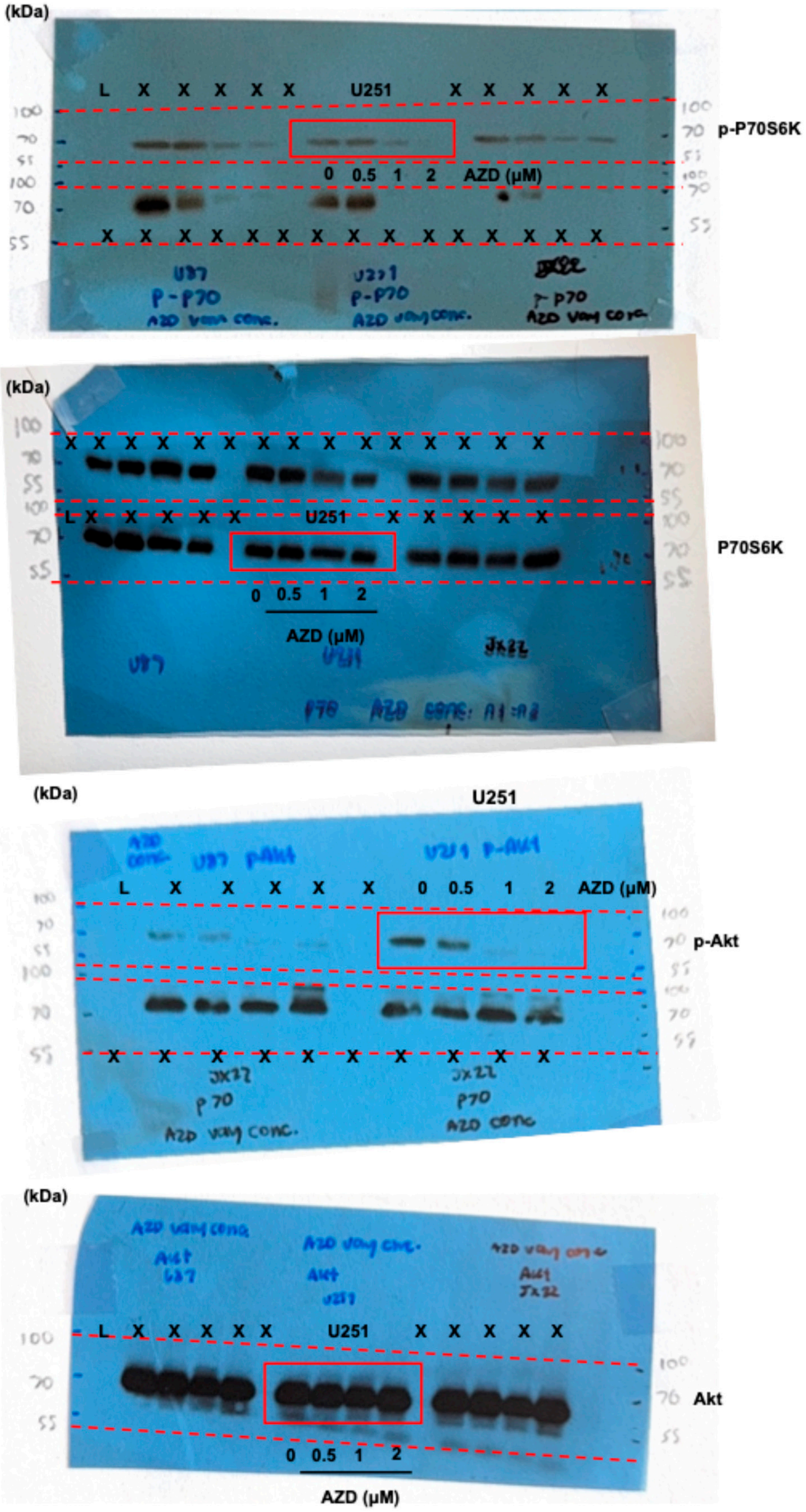
(kDa)
L X X X X X U251 X X X X X
p-P70S6K
0 0.5 1 2 AZD (µM)
X X X X X X X X X X X X X X X
(kDa)
X X X X X X X X X X X X X X X
L X X X X X U251 X X X X X
P70S6K
0 0.5 1 2
AZD (µM)
(kDa)
U251
L X X X X X 0 0.5 1 2 AZD (µM)
p-Akt
X X X X X X X X X X
(kDa)
L X X X X X U251 X X X X X
Akt
0 0.5 1 2
AZD (µM)

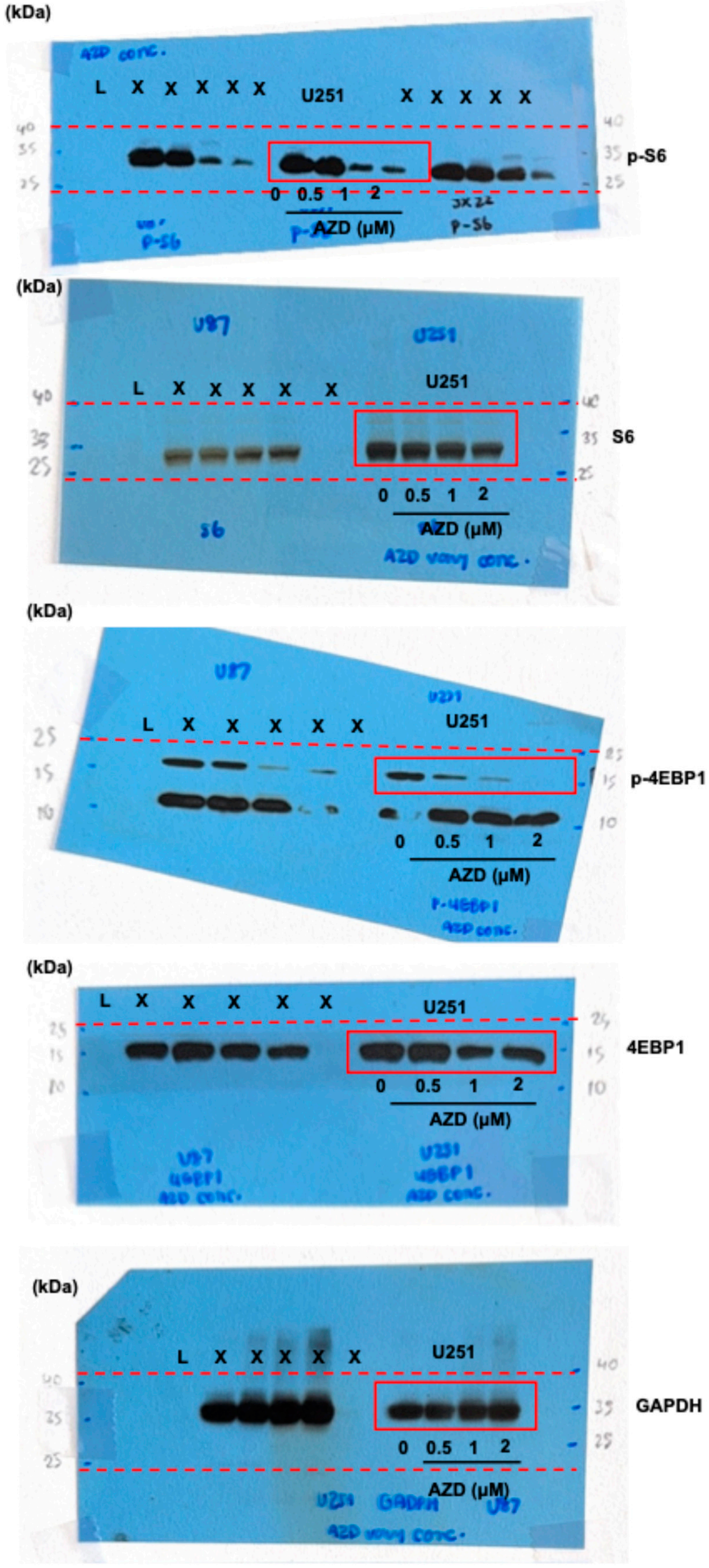
(kDa)
L X X X X X U251 X X X X X
p-S6
0 0.5 1 2
AZD (μM)
(kDa)
L X X X X X U251
S6
0 0.5 1 2
AZD (μM)
(kDa)
L X X X X X U251
p-4EBP1
0 0.5 1 2
AZD (μM)
(kDa)
L X X X X X U251
4EBP1
0 0.5 1 2
AZD (μM)
(kDa)
L X X X X X U251
GAPDH
0 0.5 1 2
AZD (μM)

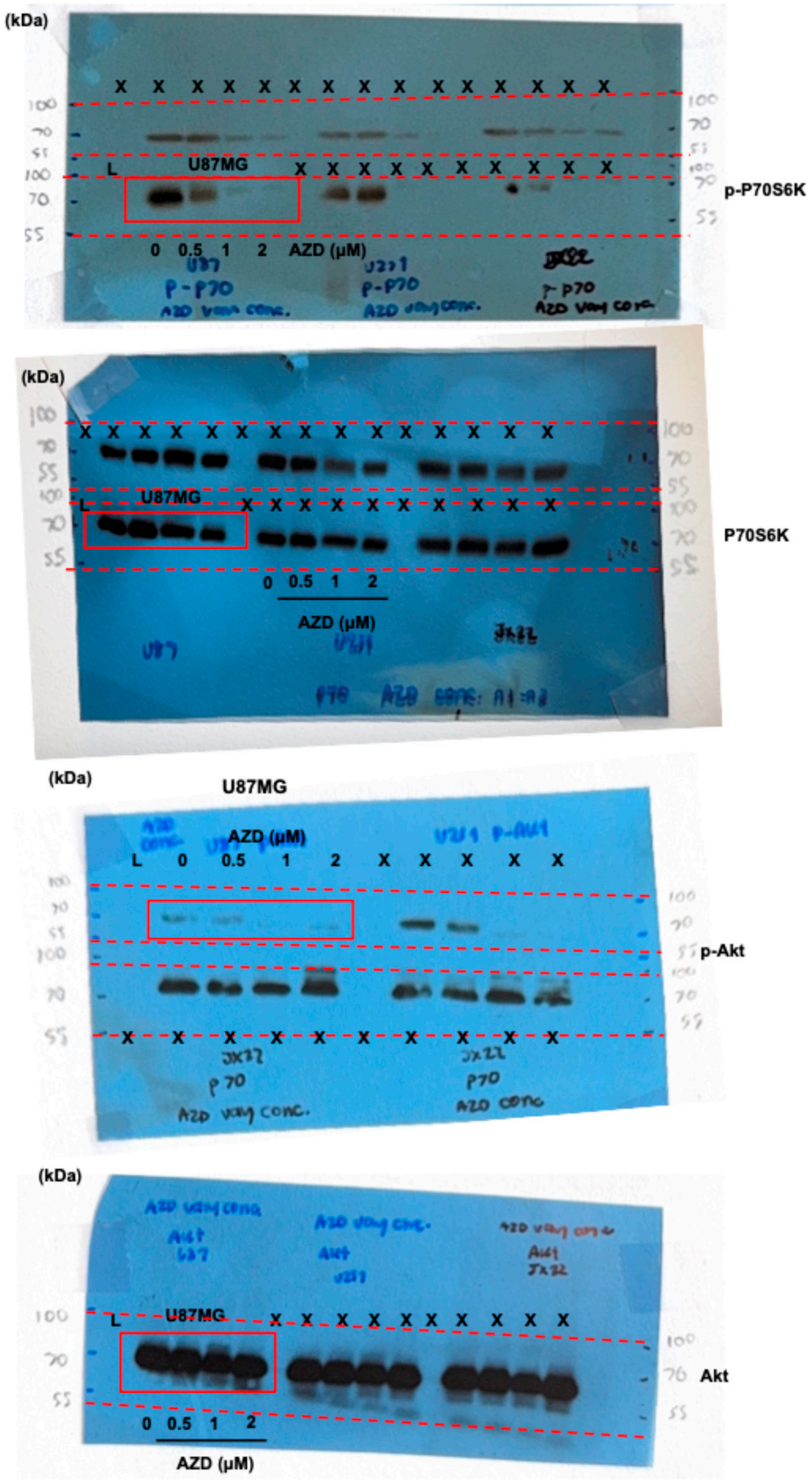

(kDa)
U87MG
0 0.5 1 2 AZD (μM)
p-P70S6K
(kDa)
U87MG
0 0.5 1 2
AZD (μM)
P70S6K
(kDa)
U87MG
AZD (μM)
L 0 0.5 1 2
p-Akt
(kDa)
U87MG
0 0.5 1 2
AZD (μM)
Akt

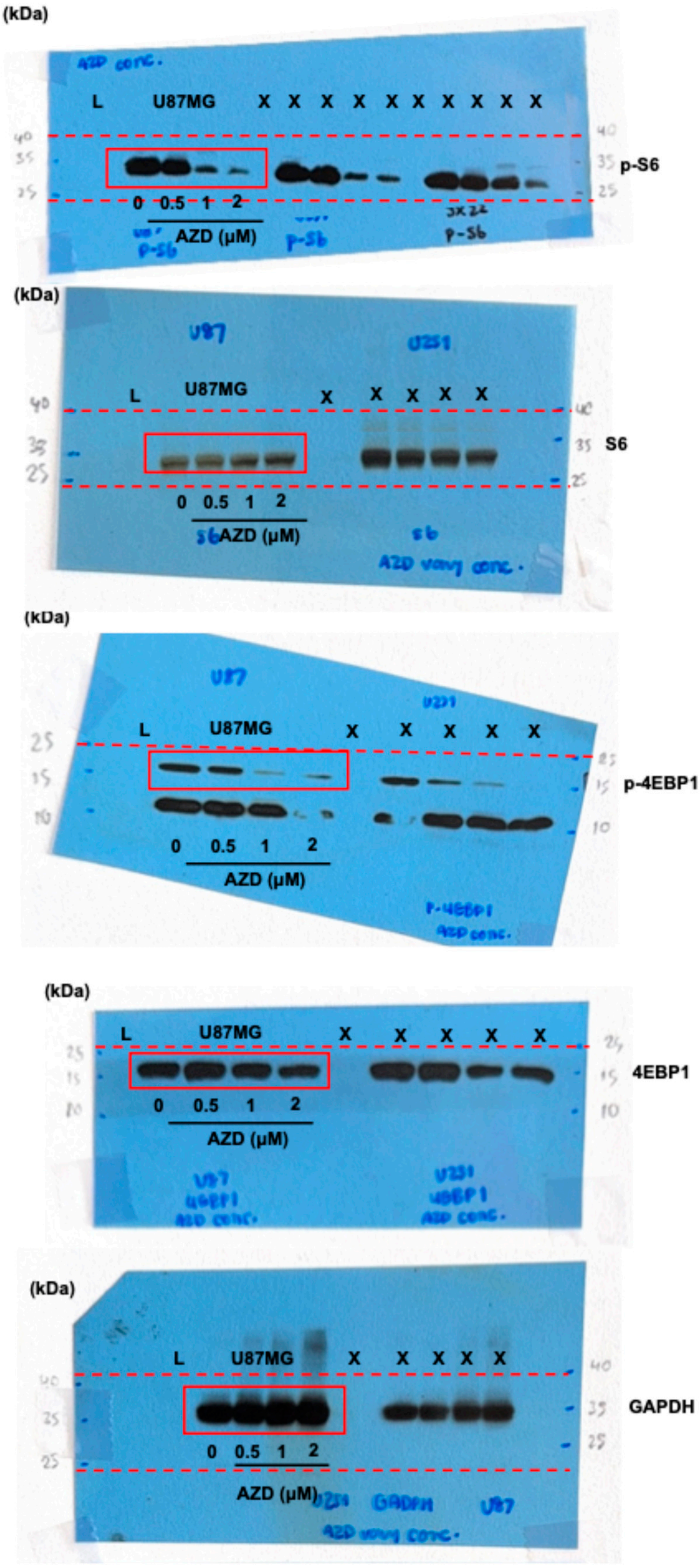
(kDa)
L U87MG X X X X X X X X X X
p-S6
0 0.5 1 2
AZD (μM)
(kDa)
L U87MG X X X X X
S6
0 0.5 1 2
AZD (μM)
(kDa)
L U87MG X X X X X
p-4EBP1
0 0.5 1 2
AZD (μM)
(kDa)
L U87MG X X X X X
4EBP1
0 0.5 1 2
AZD (μM)
(kDa)
L U87MG X X X X X
GAPDH
0 0.5 1 2
AZD (μM)

Supplementary Figure S6. Unedited original western blot images corresponding to the data presented in Supplementary Figure S10A-B

The detected proteins and their expected molecular weights are as follows: p-p70S6K (70 kDa), p70S6K (70 kDa), p-Akt (60 kDa), Akt (60 kDa), GAPDH (37 kDa), p-S6 (32 kDa), S6 (32 kDa), p-4EBP1 (15-20 kDa), and 4EBP1 (15-20 kDa). Molecular weight markers were annotated according to the protein ladder reference. Dashed lines indicate regions where membranes were sectioned prior to incubation with the respective primary antibodies. Red boxes indicate the lanes included in the final assembled figure corresponding to the labeled proteins. "L" denotes the molecular weight ladder lane, whereas "X" indicates lanes that were excluded from the final figure.

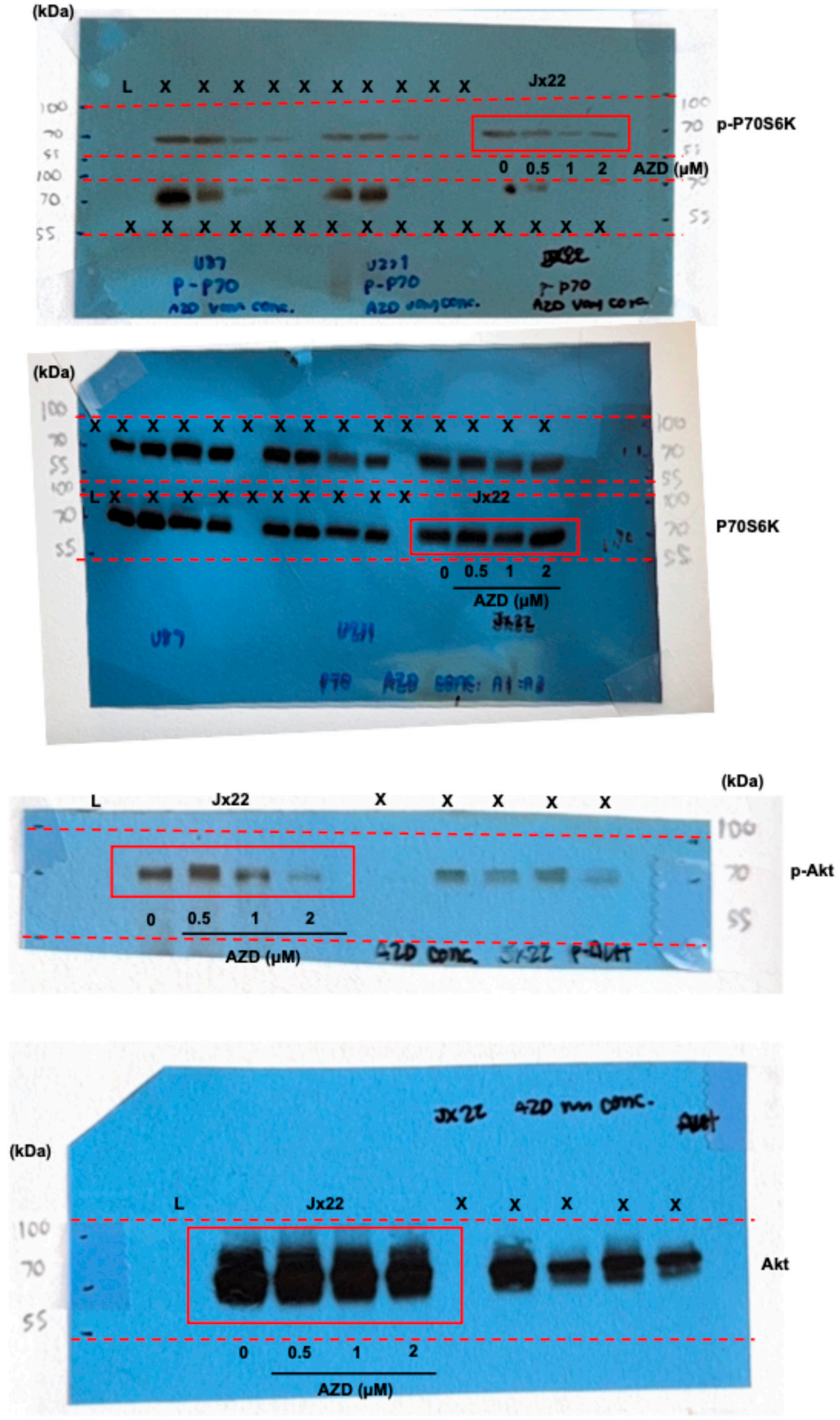
(kDa)
L X X X X X X X X X X Jx22
p-P70S6K
0 0.5 1 2 AZD (μM)
(kDa)
P70S6K
Jx22
0 0.5 1 2
AZD (μM)
(kDa)
L Jx22 X X X X X
p-Akt
0 0.5 1 2
AZD (μM)
(kDa)
L Jx22 X X X X X
Akt
0 0.5 1 2
AZD (μM)

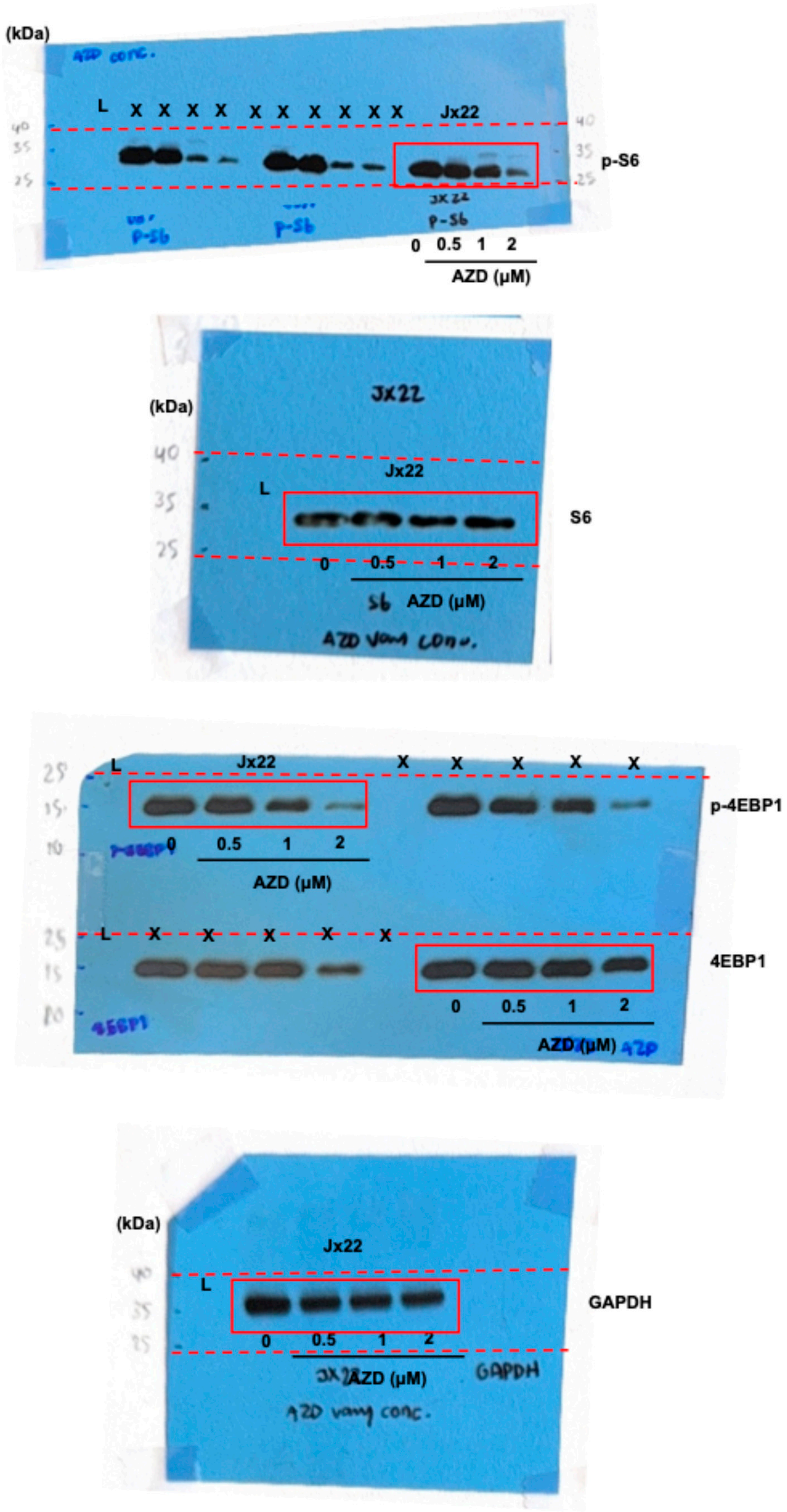
(kDa)
L
X X X X X X X X X X
Jx22
p-S6
0 0.5 1 2
AZD (µM)
(kDa)
Jx22
L
S6
0 0.5 1 2
AZD (µM)
L
Jx22
X X X X X
p-4EBP1
0 0.5 1 2
AZD (µM)
L X X X X X
4EBP1
0 0.5 1 2
AZD (µM)
(kDa)
Jx22
L
GAPDH
0 0.5 1 2
AZD (µM)

Supplementary Figure S7. Unedited original western blot images corresponding to the data presented in Supplementary Figure S10C

The detected proteins and their expected molecular weights are as follows: p-p70S6K (70 kDa), p70S6K (70 kDa), p-Akt (60 kDa), Akt (60 kDa), GAPDH (37 kDa), p-S6 (32 kDa), S6 (32 kDa), p-4EBP1 (15-20 kDa), and 4EBP1 (15-20 kDa). Molecular weight markers were annotated according to the protein ladder reference. Dashed lines indicate regions where membranes were sectioned prior to incubation with the respective primary antibodies. Red boxes indicate the lanes included in the final assembled figure corresponding to the labeled proteins. “L” denotes the molecular weight ladder lane, whereas “X” indicates lanes that were excluded from the final figure.

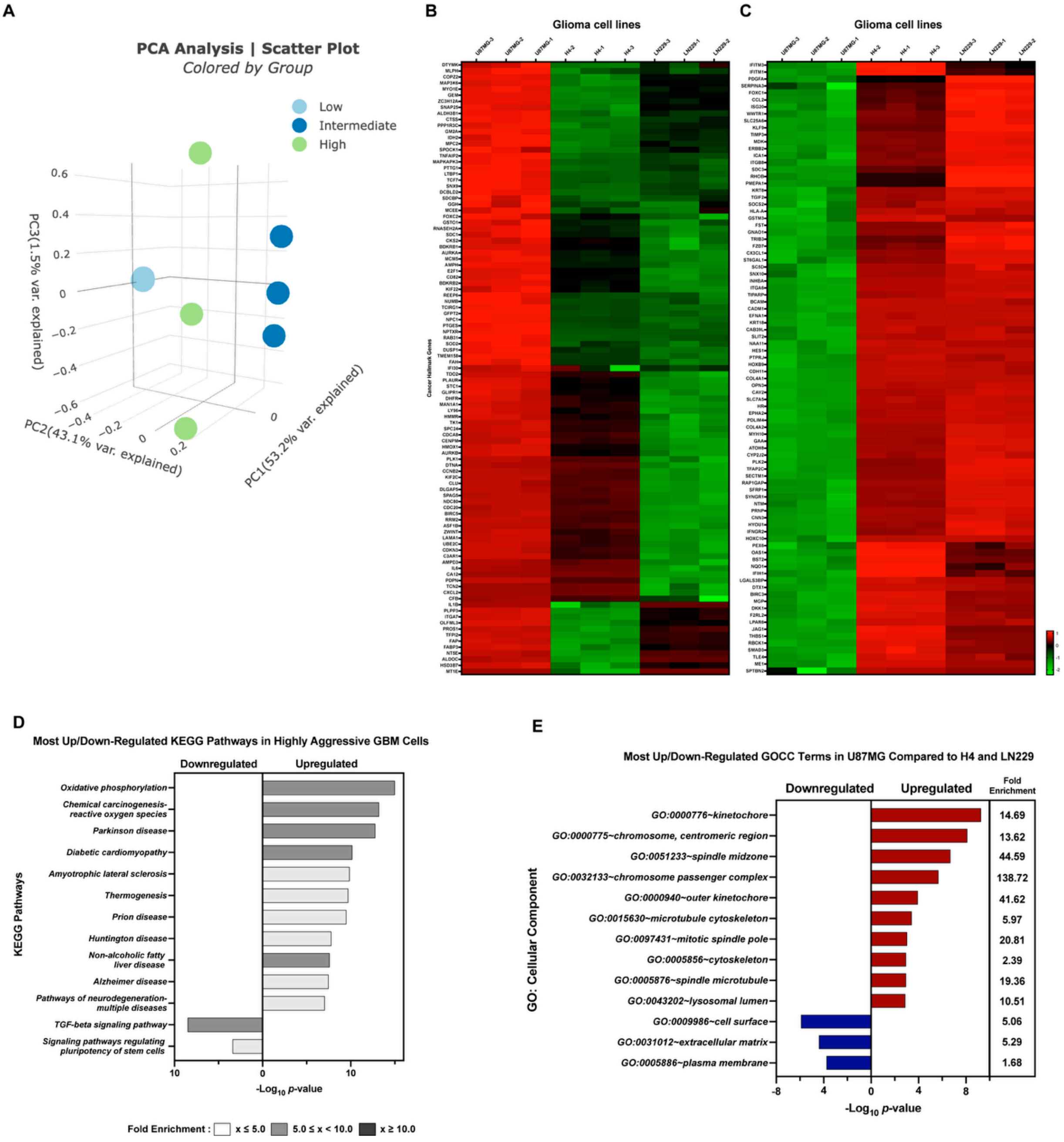

Supplementary Figure S8. Global transcriptomic features of glioma cell lines

(A) Principal component analysis of global gene expression profiles for H4, LN229, and U87MG generated using BioJupies.

(B, C) Hierarchically clustered heatmaps showing expression of cancer hallmark genes in the three cell lines. Genes upregulated and downregulated in U87MG relative to H4 and LN229 are shown in panels B and C, respectively.

(D) KEGG pathway enrichment analysis of genes differentially expressed in U87MG. Bars represent $-\log_{10}$(p-value), with colors indicating fold enrichment.
(E) GO cellular component (GOCC) enrichment analysis comparing U87MG with H4 and LN229. Bars represent $-\log_{10}$(p-value), and fold enrichment values are indicated.

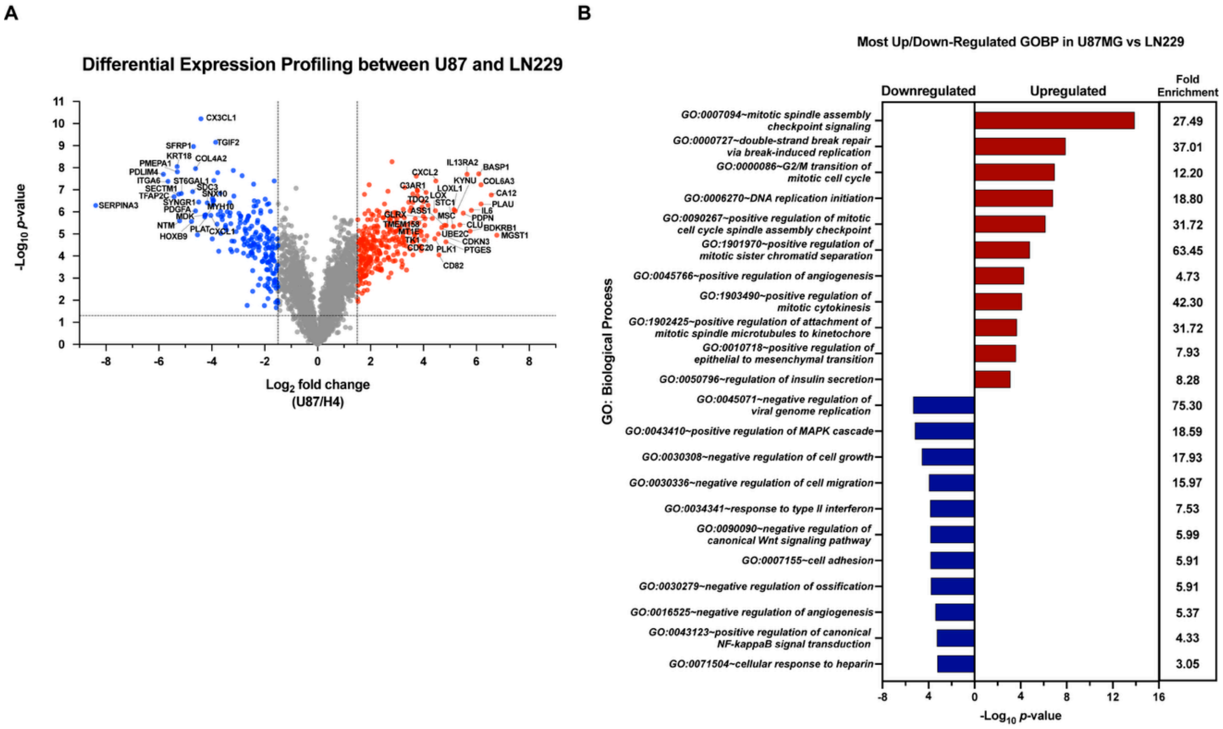

Supplementary Figure S9. Differential expression analysis of cancer hallmark genes between U87MG and LN229

(A) Volcano plot showing differential expression of cancer hallmark genes between U87MG and LN229. Significantly upregulated genes ($log_2FC > 1.5$, $p < 0.05$) are shown in red, and downregulated genes ($log_2FC < -1.5$, $p < 0.05$) in blue.
(B) GOBP enrichment analysis of differentially expressed hallmark genes between U87MG and LN229 ($|log_2FC| > 1.5$, $p < 0.05$). Top enriched terms passing FDR correction (FDR < 0.05) are shown, with bar length representing $-log_{10}$(p-value) and color indicating fold enrichment.

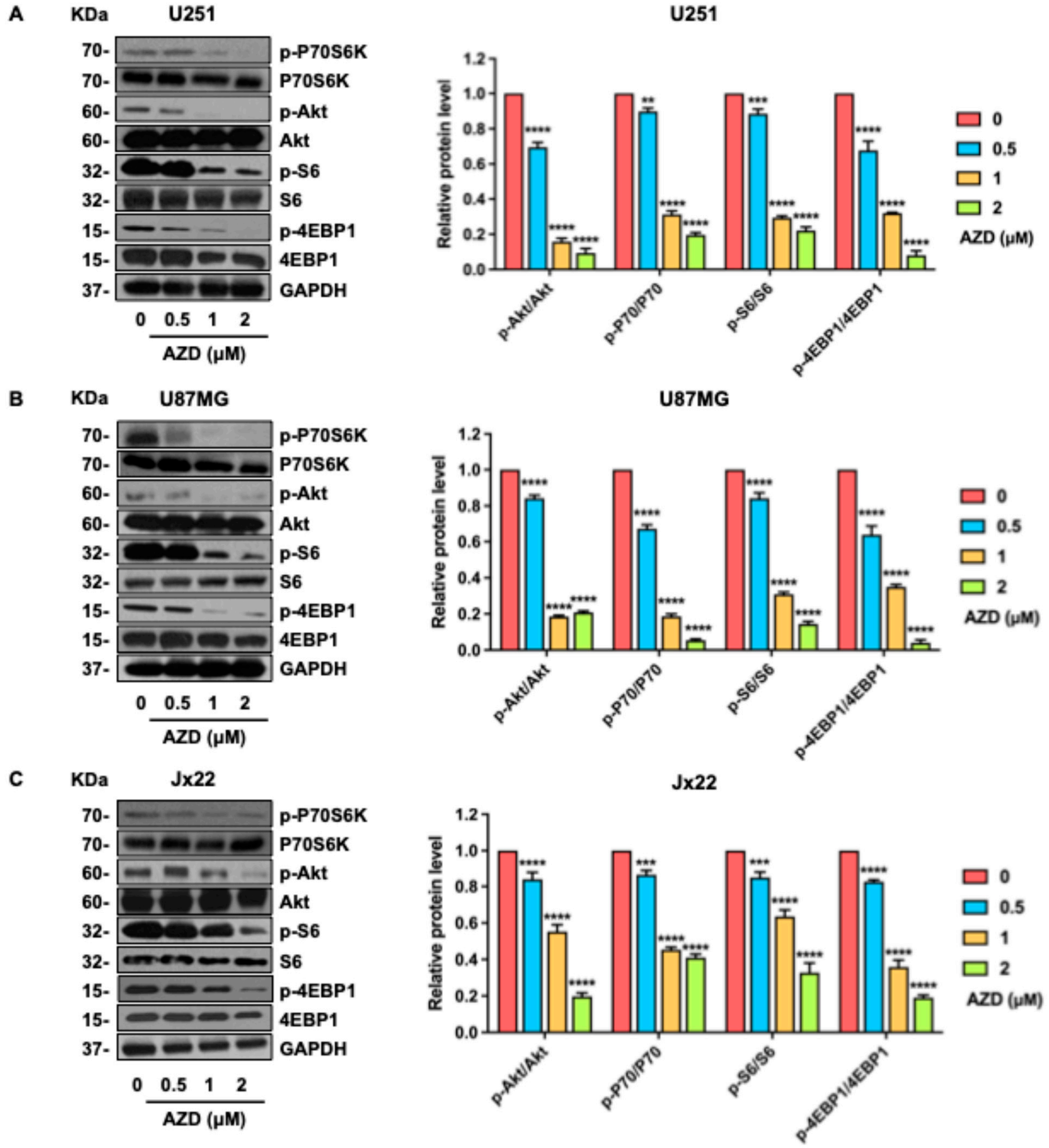

Supplementary Figure S10. AZD8055 inhibits mTORC1 and mTORC2 signaling in glioblastoma cells in a dose-dependent manner

Established glioblastoma cell lines ((A) U251 and (B) U87MG) and the patient-derived glioblastoma model ((C) Jx22) were treated with increasing concentrations of the dual mTORC1/2 inhibitor AZD8055 (0.5–2 μM) for 24 h. Inhibition of mTORC1 signaling was assessed by reduced phosphorylation of p70S6K (Thr389), S6 (Ser235/236), and 4EBP1 (Ser65), while suppression of mTORC2 activity was evaluated by decreased Akt phosphorylation at

Ser473 using western blot analysis. Representative immunoblots from three independent experiments are shown. Quantification of phosphorylated proteins was normalized to total protein levels and expressed relative to untreated controls. Data are presented as mean ± SEM ($n = 3$ independent biological experiments, each with three technical replicates). Statistical analysis was performed using one-way ANOVA followed by Tukey's post hoc test, with significance defined as **, $p < 0.01$; ***, $p < 0.001$; ****, $p < 0.0001$ *v.s.* Ctrl.

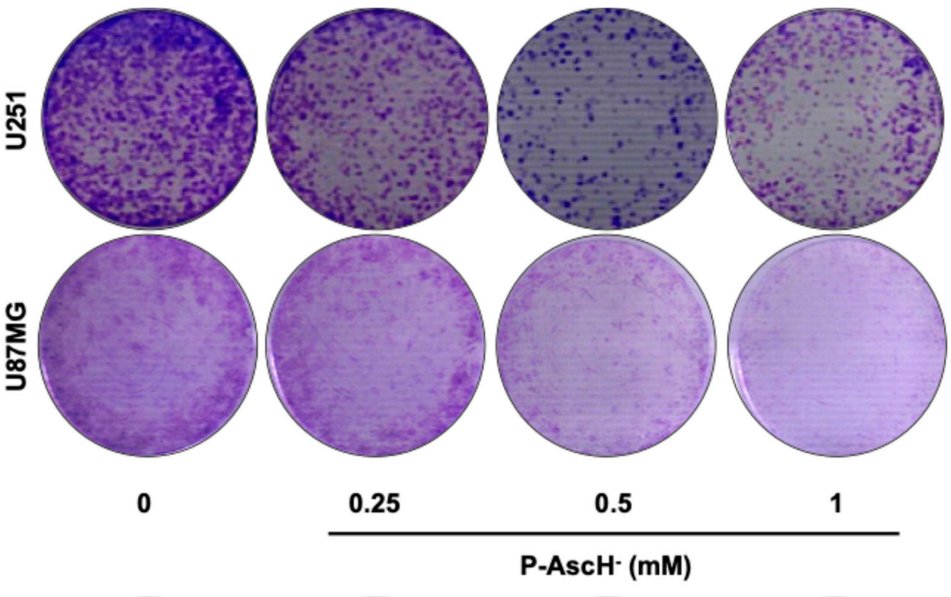

Supplementary Figure S11. Sub-cytotoxic pharmacological ascorbate impairs clonogenic survival of glioblastoma cells

U251 and U87MG cells were treated with sub-cytotoxic concentrations of P-AscH⁻ (0.25–1 mM), as defined by MTT viability and short-term proliferation assays, for 1 h. Following treatment, cells were replated and allowed to grow under drug-free conditions to assess long-term clonogenic survival. P-AscH⁻ exposure markedly reduced colony-forming capacity despite minimal effects on short-term viability. Data are representative of three independent experiments.
